# An evolution-inspired strategy to design disulfide-rich peptides tolerant to extensive sequence manipulation†

**DOI:** 10.1039/d1sc02952e

**Published:** 2021-07-22

**Authors:** Jun Zha, Jinjing Li, Shihui Fan, Zengping Duan, Yibing Zhao, Chuanliu Wu

**Affiliations:** Department of Chemistry, College of Chemistry and Chemical Engineering, The MOE Key Laboratory of Spectrochemical Analysis and Instrumentation, State Key Laboratory of Physical Chemistry of Solid Surfaces, Xiamen University Xiamen 361005 P.R. China chlwu@xmu.edu.cn; College of Continuing Education, Guizhou Minzu University Guiyang 550025 China

## Abstract

Natural disulfide-rich peptides (DRPs) are valuable scaffolds for the development of new bioactive molecules and therapeutics. However, there are only a limited number of topologically distinct DRP folds in nature, and most of them suffer from the problem of *in vitro* oxidative folding. Thus, strategies to design DRPs with new constrained topologies beyond the scope of natural folds are desired. Herein we report a general evolution-inspired strategy to design new DRPs with diverse disulfide frameworks, which relies on the incorporation of two cysteine residues and a random peptide sequence into a precursor disulfide-stabilized fold. These peptides can spontaneously fold in redox buffers to the expected tricyclic topologies with high yields. Moreover, we demonstrated that these DRPs can be used as templates for the construction of phage-displayed peptide libraries, enabling the discovery of new DRP ligands from fully randomized sequences. This study thus paves the way for the development of new DRP ligands and therapeutics with structures not derived from natural DRPs.

## Abstract 2

A general method was developed to design multicyclic peptides with diverse disulfide frameworks amenable to random peptide library design, enabling the development of new disulfide-rich peptide ligands and therapeutics with structures not derived from natural peptides.

## Graphical abstract

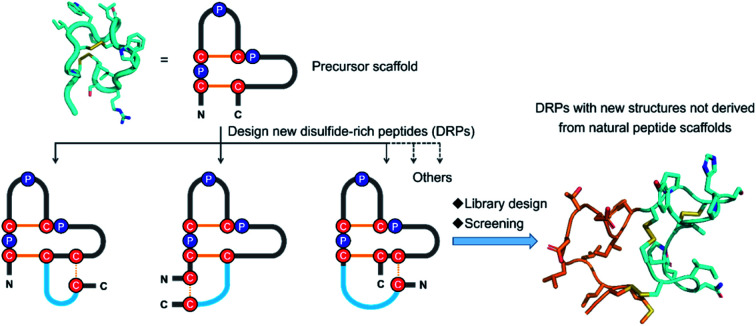

## Introduction

Disulfide-rich peptides (DRPs) represent a unique class of constrained peptides that are widely distributed in nature.^1,2^ These peptides, including conotoxins, cyclotides and knottins, have many unique structural and functional features, which can regulate cell signaling and immunity by inhibiting proteases, blocking channels and binding receptors.^3–6^ To generate new bioactive DRPs, these naturally occurring peptides can be re-engineered through epitope grafting or library screening.^7–10^ The newly designed DRPs usually possess the merits of their precursor scaffolds in terms of stability and bioavailability.^7,11–14^ However, there are only a limited variety of disulfide-stabilized peptide folds in nature, significantly limiting the development of potent DRPs for new targets. Recently, computation-based methods have also been developed for *de novo* design of DRPs with precisely controlled topologies, though the designed peptides consist of mainly regular α-helix and β-strand structures.^15,16^ Methods to design DRPs with new structures or constrained topologies beyond the scope of both naturally occurring and computationally designed disulfide-stabilized peptide folds are still desired.^17^

**Fig. 1:**
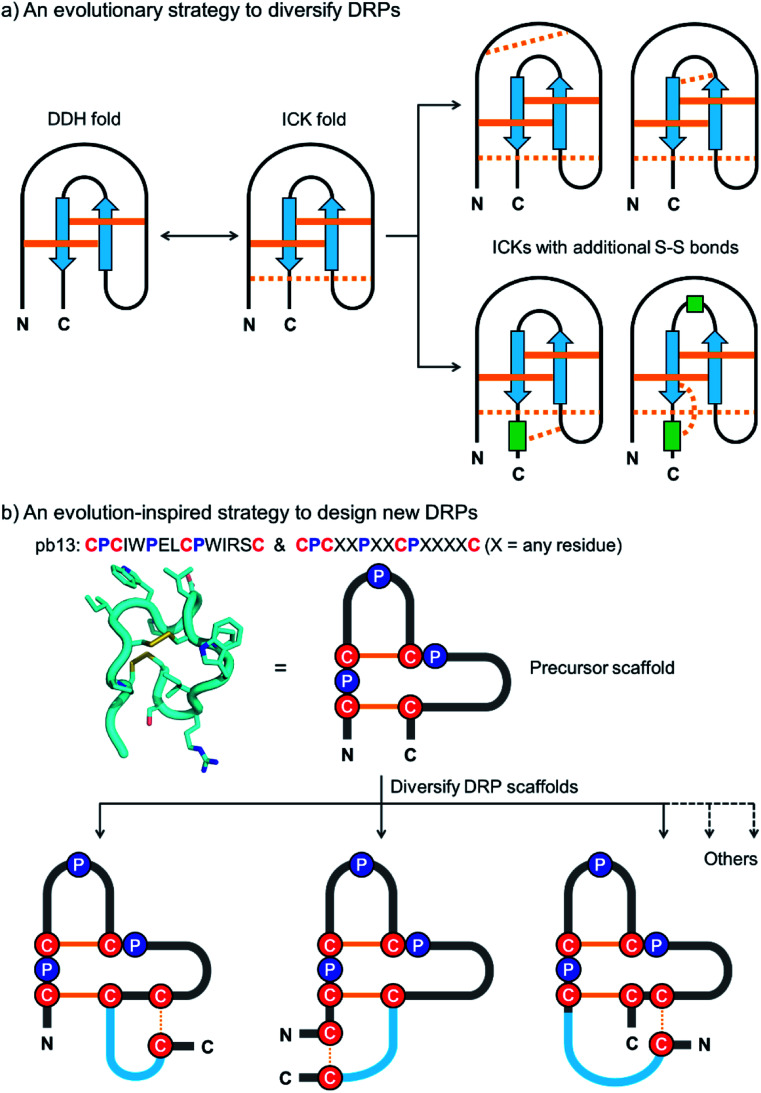
(a) Schematic view of the evolution strategy to diversify the naturally occurring DRPs. Conserved and non-conserved disulfide bonds were depicted with solid and dashed orange lines, respectively. Blue arrows and green rectangles denote peptide segments with β-strand and α-helix structures, respectively. DDH: disulfide-directed hairpin; ICK: inhibitor cystine knot. (b) Our strategy inspired by natural evolution to design new DRPs. The structure of pb13 (PDB ID: 6IMG) was illustrated using a simplified scheme with the conserved cysteine/proline framework highlighted. Conserved disulfide bonds and the additionally inserted disulfide bond were depicted with solid and dashed orange lines, respectively. Blue lines denote the grafted peptide segments.

The design and engineering of DRPs are challenging because of the difficulty of directing the precise pairing of cysteines into specific disulfide connectivities.^18–22^ Natural evolution to overcome this challenge is manifold.^23,24^ One strategy that is essential to the diversification of the DRPs in nature without complicating their disulfide pairings is to diversify a small number of primordial and elementary disulfide-stabilized folds with fewer disulfides by cysteine mutations to introduce additional disulfide bonds (Fig. 1a).^25,26^ This evolution process divides the disulfides in the natural DRPs into conserved ones essential to the integrity of the primary folds and less conserved ones contributing to the structural diversification, thus greatly favoring their oxidative folding into native structures.^27,28^ Some privileged DRPs with three or more disulfide bonds are thought to be generated through this way, including inhibitory cystine knots, three-finger toxin folds, and cystine-stabilized α/β folds.^25,29–31^ Interestingly, this evolution strategy has also been adopted by the bovine immune system to diversify disulfide frameworks of antigen-binding regions of bovine antibodies.^32,33^ Despite this, this straightforward, robust and intriguing strategy has never been applied artificially for the design and engineering of DRPs with new structures or constrained topologies.

In this work, we describe a general and robust method to design a class of new DRPs with diverse disulfide frameworks that are easy to fold and amenable to random peptide library design and screening. Inspired by the evolution strategy to diversify naturally occurring DRPs, we conceived that new DRPs can be designed by rationally grafting an additional disulfide along with a random peptide sequence into precursor peptide folds with fewer disulfide bonds but conserved tertiary structures (Fig. 1b). If the precursor disulfide-stabilized folds selected for structural diversifications are tolerant to sequence variations, the newly designed DRPs should inherit the easy-to-fold properties of the precursors and be amenable to an exceptionally high degree of sequence randomization. Existing disulfide-stabilized folds satisfying the demands as precursor scaffolds are rare, but fortunately a bicyclic peptide with a unique cysteine/proline framework recently discovered in our lab provides an ideal scaffold to our proposed design (Fig. 1b).^34^ With the success of the design of DRPs with different disulfide frameworks, libraries of DRPs were then designed and screened against model protein targets to create new DRPs with protein-binding activities. This work thus provides a class of new DRPs with all amino acid residues within the scaffolds, except the conserved cysteines and prolines, being able to be randomly mutated for functional evolution, a unique feature that is not possessed by the naturally occurring DRPs.

## Results and discussion

**Fig. 2:**
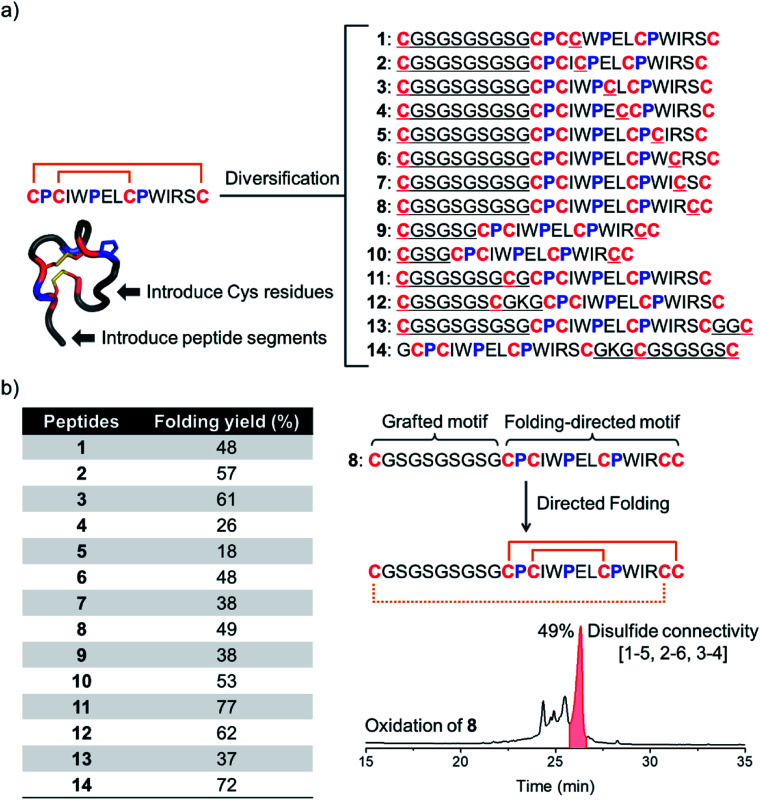
(a) Model peptides designed by introducing two additional cysteine residues and a new peptide segment into the pb13 core. (b) Folding yields of these peptides in redox buffers evaluated using HPLC by the peak area normalization method. HPLC chromatogram showing the oxidation of **8** (50 μM) in a GSH/GSSG redox buffer (pH 7.4).

We have recently discovered a unique bicyclic scaffold with a CPCX_2_PX_2_CPX_4_C (C, P and X are cysteine, proline and any residue respectively; the digits are numbers of the residue X) framework from a peptide library screening, which consists of four irregular turns and a short 3_10_ helix.^34^ It was found that peptides with the above cysteine/proline framework can fold into the native bicyclic topology (*i.e.*, **1–4** and **2–3** disulfide connectivity) with >95% yields in redox buffers.^34^ Thus, we strategically diversified this bicyclic peptide scaffold through the incorporation of two additional cysteines and a new peptide segment (Fig. 1b and 2a). Firstly, by taking the model peptide pb13 as a precursor, one of the eight non-conserved residues (X) was replaced with cysteine and at the N-terminus a flexible cysteine-bearing 10-mer peptide segment was grafted, generating eight peptides with six cysteine residues (**1–8**; Fig. 2a). These peptides were synthesized using solid-phase synthesis and purified to >95% using reverse-phase HPLC (Fig. S1†). We then examined the oxidative folding of these peptides in redox buffers of glutathione (GSH)/oxidized GSH (GSSG) using HPLC (Fig. S2†). To identify the folding products with the desired disulfide connectivity, we further synthesized peptides **S1–S8**, in which the two incorporated cysteines were orthogonally protected with acetamidomethyl (Acm). After the oxidative pairing of the two conserved disulfide bonds in the pb13 segment of **S1–S8**, the third disulfide bond can be selectively paired through the deprotection of Acm, leading to the formation of the expected tricyclic peptides (as standard products; Fig. S3 and S4†). Accordingly, by comparing the folding products of **1–8** and the standard products synthesized through the Acm-protecting group strategy in HPLC chromatograms (Fig. S5†), the folding yield to the expected tricyclic peptides from the oxidation of **1–8** can be obtained (Fig. 2b). We found that all of these peptides can efficiently fold into the expected tricyclic structures (yields: 18–61%), implying the strong driving force of the pb13 segment in directing the oxidative folding of these peptides. Interestingly, among the eight peptides, seven tricyclic peptides (except **5**) can be obtained as the major products after the oxidation. Considering that peptides with six cysteine residues can statistically form fifteen isomers with different disulfide connectivities, the folding yields of these peptides are relatively high. In addition, by taking **8** as an example, we demonstrated that the variation of the length of the grafted loop does not significantly affect the oxidative folding (**9** and **10**; Fig. S6†).

To design more tricyclic peptide scaffolds, we further altered the position of the incorporated cysteine residues. Peptides **11–14** were designed and synthesized (Fig. S7†), which either contain two additional cysteines at the N- or C-terminus or contain two distantly separated N- and C-terminal cysteines (Fig. 2a). These peptides can fold into the expected tricyclic structures as the major products (yields: 37–77%; Fig. S8–S10†), further verifying the strong folding-directing capability of the cysteine/proline-rich frameworks.

**Fig. 3:**
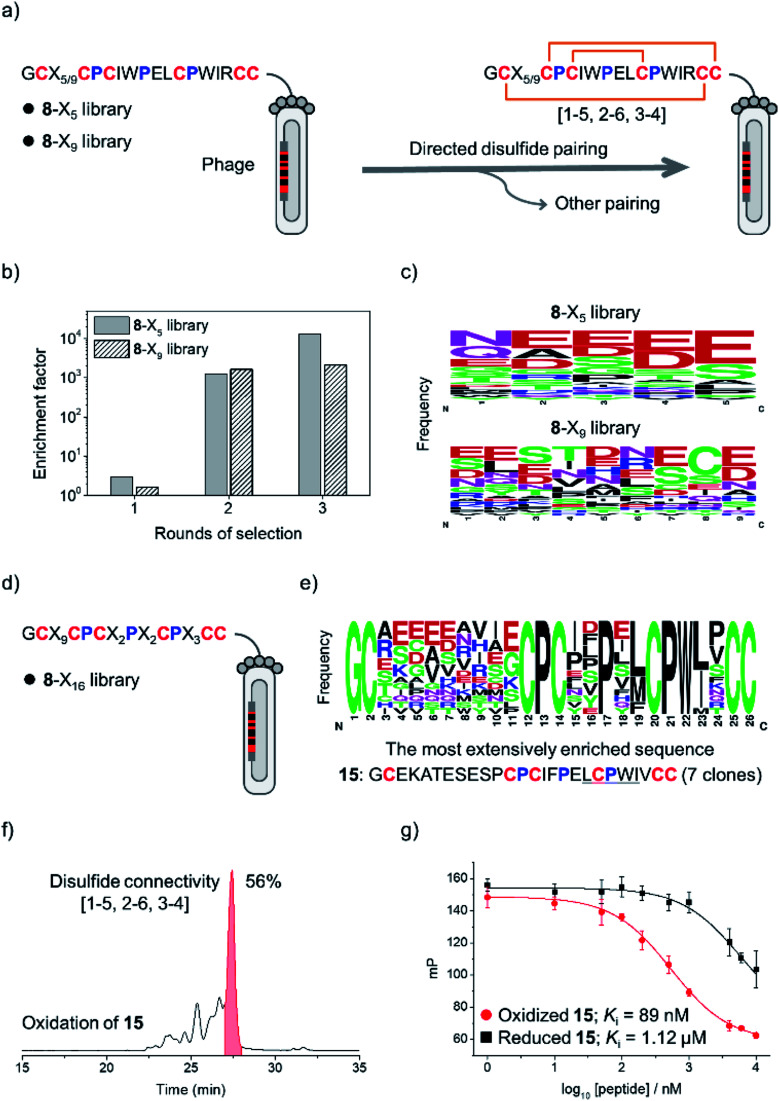
(a) Design of two phage-displayed peptide libraries using the cysteine/proline framework of the model peptide **8** (**8**-X_5_ and **8**-X_9_ library). (b) Phage titers enriched after iterative rounds of selection against MDM2. (c) WebLogo showing the distribution of amino acid residues in the random peptide segment after three rounds of selection. (d) Design and screening of the **8**-X_16_ library. (e) Consensus sequences shown in WebLogo were obtained after three rounds of selection of the **8**-X_16_ library against MDM2. (f) HPLC chromatogram showing the oxidation of **15** (50 μM) in a GSH/GSSG redox buffer (pH 7.4). Other peaks come from folding byproducts with alternative unidentified disulfide connectivities. (g) A fluorescence polarization competition assay showing the binding of reduced and oxidized **15** to MDM2. All peptides used for the binding affinity characterization were purified using HPLC to a purity of >95%.

The above results clearly demonstrated the feasibility of designing new DRPs with diverse disulfide frameworks by incorporating new peptide segments and additional cysteine residues into a precursor disulfide-stabilized fold. To further prove that the designed DRPs are tolerant to sequence randomization, we selected **8** as a model scaffold to construct phage-displayed DRP libraries. Firstly, the grafted loop of **8** was randomized on the surface of phages using NNK codon degeneracy to encode random residues as we described previously (Fig. S11†).^17^ Two libraries with different lengths of the randomized loop were designed and prepared (**8**-X_5_ and **8**-X_9_; Fig. 3a). The transformation of phagemid vectors into *E. coli* yielded a library size of 4.8 × 10^8^ (*i.e.*, saturation coverage) and 1.4 × 10^9^ for **8**-X_5_ and **8**-X_9_, respectively. As **8** preserves binding capability to the protein target of pb13 (*i.e.*, MDM2; Fig. S12†),^34^ we speculated that properly folded clones can be identified through screening based on the binding ability to MDM2. Thus, the two libraries were applied to screening against MDM2, and we observed extensive enrichment of phages relative to the control group after the first round of selection (Fig. 3b). After three rounds of selection, 20–30 clones were randomly picked and sequenced. Interestingly, we observed no enrichment of a specific sequence, instead the enriched sequences display a high diversity (Fig. 3c and S13†), suggesting the tolerance of the newly designed DRP scaffolds (*e.g.*, **8**) to a variety of random sequences in the grafted loops.

Then, a new library based on the scaffold **8** with all residues randomized except cysteines and prolines was designed and prepared (**8**-X_16_; library size: 1.1 × 10^9^; Fig. 3d). The library was also applied to screening against MDM2, and we observed extensive enrichment of phages after three rounds of selection. The analysis of sequences from randomly picked clones indicates an abundant enrichment of specific clones featuring a consensus sequence of L/M-C-P-W-I/L (Fig. 3e and S14†), which is a typical motif with MDM2 binding capability.^34^ The most abundantly enriched sequence was then synthesized (peptide **15**). Interestingly, the oxidation of **15** produces a major product with a yield of 56% (Fig. 3f), which exhibits a nanomolar affinity to MDM2 (*K*_i_ = 89 nM; >10-fold higher than the affinity of the reduced linear **15**), as determined by a reported fluorescence polarization competition assay (Fig. 3g).^17^ Furthermore, two-dimensional (2D) NMR characterization verified that the oxidized **15** has the expected disulfide connectivity (**1–6**, **2–5**, and **3–4**; Fig. S15†). These results thus demonstrated the feasibility of selecting peptide ligands to proteins of interest from DRP libraries with three disulfide bonds and an unprecedentedly high degree of sequence randomization.

**Fig. 4:**
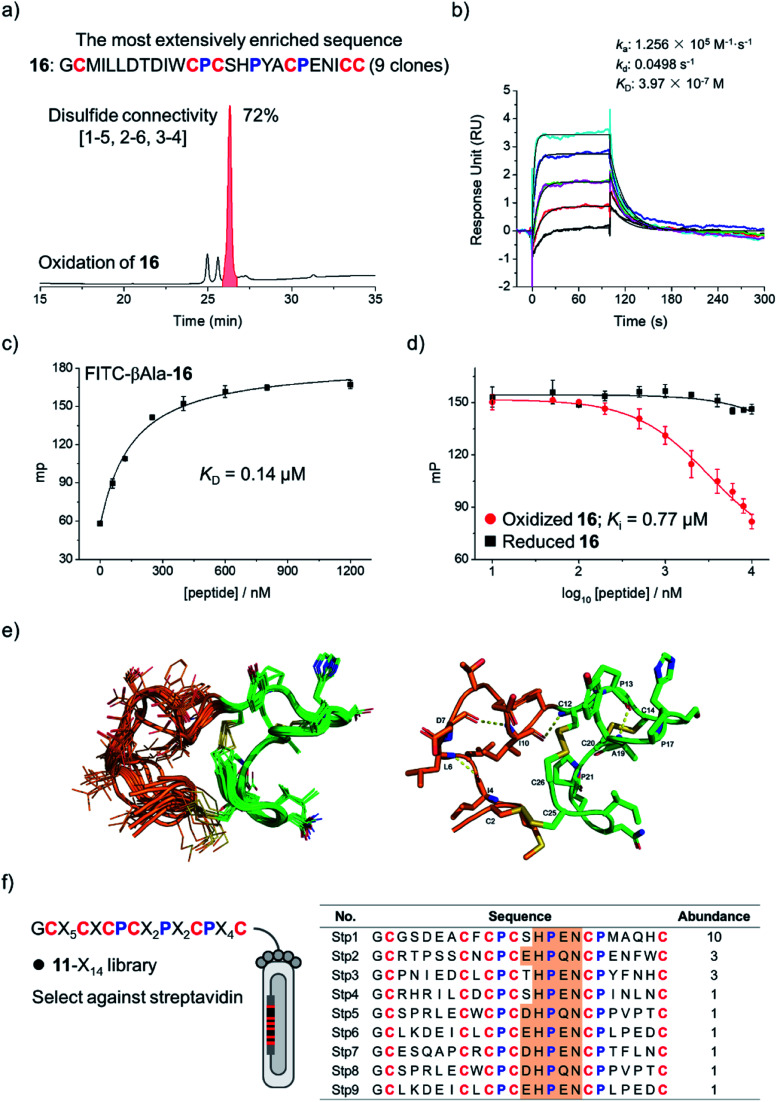
(a) Sequence of **16** most extensively enriched after the selection of the **8**-X_16_ library against Bcl-2, and HPLC chromatogram showing the oxidation of **16** (50 μM) in a GSH/GSSG redox buffer (pH 7.4). (b) Sensorgrams recorded by surface plasmon resonance (SPR) showing the direct binding kinetics of oxidized **16** (0.16–2.56 μM) with Bcl-2. (c) Binding of oxidized **16** labelled with a fluorophore (FITC-βAla-**16**) to Bcl-2 recorded using a fluorescence polarization assay. (d) Binding of oxidized and reduced **16** to Bcl-2 by competition with FITC-βAla-**16** in a fluorescence polarization assay. (e) Solution structures of **16**. Left: 10 structures superimposed by all heavy atoms; the grafted N-terminal loop and the cysteine/proline-rich domain are highlighted in orange and green, respectively. Right: the lowest-energy NMR structure of **16**. (f) Design of a new library (**11**-X_14_). Sequences of peptides isolated from the selection of the **11**-X_14_ library against streptavidin.

To examine if the library **8**-X_16_ can be applied to select new DRP ligands to other targets, Bcl-2 was then selected as a new model protein, which is an antiapoptotic protein closely associated with cancer.^17,35,36^ After four rounds of selection, 20 phage clones were randomly picked and sequenced, giving 12 unique amino acid sequences, in which one sequence was extensively enriched (9 clones; Fig. S16†). This enriched sequence was then synthesized (peptide **16**), and its oxidative folding was analyzed using HPLC. Peptide **16** was oxidized to yield the expected tricyclic structure as a major product (72% yield; Fig. 4a). The binding kinetics and affinity of the oxidized **16** (purified by HPLC) with Bcl-2 were then determined using surface plasmon resonance (SPR). The peptide can bind to Bcl-2 with a *K*_D_ of 397 nM (Fig. 4b), whereas its linear counterpart (*i.e.*, Cys-to-Ala mutant) exhibits negligible affinity to the target (Fig. S17†). This result was further confirmed using fluorescence polarization assays (Fig. 4c and d), indicating that the tricyclic structure is crucial for the binding of the peptide to Bcl-2.

The NMR structure of the tricyclic **16** was then determined using ^1^H, ^1^H distance constrains derived from 2D ^1^H, ^1^H NOESY experiments (Fig. 4e; PDB ID: 7ELY). The tricyclic **16** consists of a rigid C-terminal cysteine/proline-rich domain (residues **12–26**; RMSD 0.15) and a relatively flexible N-terminal loop (residues **2–11**; RMSD 1.21). The backbone RMSD of the whole tricyclic structure is 0.43 ± 0.06. The N-terminal loop is stabilized by three turn-like structures with hydrogen bonds and a disulfide bond formed between Cys2 and Cys25, in which residues **7–12** form two continuous turn-like structures stabilized by *i*, *i* + 3 *and i*, *i* + 2 backbone hydrogen bonds (Asp7⋯Ile10 and Ile10⋯Cys12), respectively. The C-terminal framework is mainly stabilized by the two disulfide bonds and the rigid prolines, in which Pro13 and Pro17 mediate the formation of the Pro13⋯Tyr18 hydrogen bond, and Pro13 and Pro21 stabilize the disulfide bond of Cys14–Cys20. Therefore, the overall structure of the tricyclic **16** was stabilized together by three disulfide bonds, four backbone hydrogen bonds, and the rigid prolines (Fig. 4e). To our knowledge, this is the first structure of a peptide with up to three disulfide bonds directly selected from fully randomized sequences. This structure may further serve as a template for the design of new DRP libraries. In addition, we found that the structure of the C-terminal cysteine/proline-rich domain of the tricyclic **16** is substantially different from that of the precursor scaffold pb13, suggesting that the precursor scaffold is amenable to a relatively broad range of structural adaptations in the designed DRPs with three disulfides to generate new structures without compromising the oxidative folding. This finding thus reveals the potential of our evolution-inspired strategy to create DRPs with completely new three-dimensional structures.

To examine the generality of the concept for the development of new DRP libraries, another library was designed and prepared using the cysteine/proline-framework of **11** as a scaffold (**11**-X_14_; library size: 6.3 × 10^8^; Fig. 4f). This library was then applied to select ligands to a model target streptavidin. After three rounds of selection, 30 clones were randomly picked and sequenced. Sequence analysis indicates the extensive enrichment of specific clones featuring a consensus sequence of D/E-H-P-Q/E-N (Fig. 4f). The most abundantly enriched sequence was then synthesized (**17**), which oxidized in GSH/GSSG redox buffers to the expected tricyclic structure in 60% conversion (Fig. S18†). SPR analysis demonstrated the binding of the peptide to streptavidin with a nanomolar affinity (*K*_D_ = 38 nM; Fig. S19†), whereas the original peptide template (**11**) shows no binding affinity (Fig. S20†), indicating the successful identification of peptide ligands from the new library.

Finally, we examined the binding selectivity of the selected peptides to the relevant protein targets. Tricyclic **15** obtained from selection against MDM2 displays no binding affinity to Bcl-2 and streptavidin (Fig. S21†). Similarly, tricyclic **16** and **17** (obtained from selection against Bcl-2 and streptavidin, respectively) show negligible binding affinity to the other two proteins (Fig. S22 and S23†). This result strongly suggests the potential of our disulfide-rich peptide libraries for discovering new peptide ligands with high binding affinity and selectivity to protein targets. It is worth noting that multicyclic peptides constrained through disulfide bonds are not stable for reduction, which limits their applicability in reducing environments such as in cytosol. However, these peptides may be used for developing *in vitro* binding assays that do not involve reducing reagents by taking advantage of the high proteolytic stability of multicyclic peptides. Indeed, we found that tricyclic peptides (**15** and **16**) are significantly more stable toward proteolysis by chymotrypsin compared to their linear analogs (Fig. S24 and S25†). In addition, the tricyclic peptides (**15** and **16**) are stable to heat treatment as no aggregation was observed even after raising the solution temperature to ∼95 °C (monitored using circular dichroism; Fig. S26†). More importantly, by using these cytosolic protein targets as models, we have demonstrated the feasibility of using our disulfide-rich peptide libraries for the discovery of new peptide ligands. It is expected that these libraries will be extensively used further for developing peptide ligands to protein targets located in extracellular environments, and then *in vivo* applications of these new disulfide-rich peptides are expectable.

## Conclusion

In summary, we reported a general method to design DRPs with diverse disulfide connectivities by mimicking the natural evolution strategy that diversifies the disulfide frameworks of natural DRPs. Eleven DRP scaffolds with different disulfide connectivities were designed, and almost all of them can spontaneously fold in redox buffers into the expected tricyclic structures as the major products. We demonstrated that the designed DRP scaffolds are amenable to sequence randomization and construction of phage-displayed peptide libraries. Unlike the naturally occurring DRPs, the folding of our DRPs was primarily directed by their unique cysteine/proline frameworks, exhibiting high tolerance to extensive sequence manipulations. By applying the libraries for screening against different protein targets, we identified several DRP ligands with high affinity from fully randomized sequences. Thus, this study provides a new way to generate novel DRP scaffolds without recourse to naturally occurring scaffolds and computational designs. As many new DRP libraries can be conveniently constructed by varying both the length of the grafted loop and the pattern of cysteine residues, this work would represent as an important step toward the *de novo* development of new DRP ligands or therapeutics with structures not derived from natural peptides. One limitation of our strategy to design new DRPs is the limited availability of the precursor disulfide-stabilized folds tolerant to sequence variations. A potential way to solve this problem is to select or design new cysteine/proline frameworks that can precisely direct the folding of peptides with two disulfide bonds. A recent advance in the computational design of structured peptides indicates the potential of manipulating proline-stabilized turns to direct peptide folding.^37^ In nature, multiple conserved prolines in minicollagen cysteine-rich domains have been found to be of particular importance due to their correct oxidative folding.^38^ These findings, together with our studies, reveal the feasibility of directing the oxidative folding of peptides through manipulating cysteine/proline frameworks. Thus, we believe that many new proline-nucleating folds with two disulfide bonds can be *de novo* designed or discovered in the near future, which can then be used as precursors to design DRPs with three disulfide bonds and more rigid and complexed structures.

## Data availability

All the necessary data were provided in ESI.†

## Author contributions

J. Z., J. L. S. F. and Z. D. designed and performed experiments. J. Z., J. L. S. F. and Z. D. analyzed data. C. W. designed experiments and analyzed data. All authors contributed to writing the manuscript.

## Conflicts of interest

The authors declare no competing interests.

## Supplementary Material

SC-012-D1SC02952E-s001
