# Supplementary material for: An evolution-inspired strategy to design disulfide-rich peptides tolerant to extensive sequence manipulation
Source: Chem Sci. 2021 Jul 22;12(34):11464–72. doi: 10.1039/d1sc02952e (PMC8409457; doi:10.1039/d1sc02952e)
Supplement: SC-012-D1SC02952E-s001 [file SC-012-D1SC02952E-s001.pdf]

Electronic Supplementary Material (ESI) for Chemical Science.
This journal is © The Royal Society of Chemistry 2021

An Evolution-Inspired Strategy to Design Disulfide-Rich Peptides Tolerant to Extensive Sequence Manipulation

Jun Zha[†,‡], Jinjing Li[†], Shihui Fan[†], Zengping Duan[†], Yibing Zhao[†], Chuanliu Wu[†,*]

[†]*Department of Chemistry, College of Chemistry and Chemical Engineering, The MOE Key Laboratory of Spectrochemical Analysis and Instrumentation, State Key Laboratory of Physical Chemistry of Solid Surfaces, Xiamen University, Xiamen, 361005, P.R. China.*

[‡]*College of Continuing Education, Guizhou Minzu University, Guiyang, 550025, China*

**Corresponding Author; Email: chlwu@xmu.edu.cn.*

Table of contents

1. Experimental Section

1.1 Materials and instruments

1.2 Synthesis of peptides

1.3 Oxidation of peptides

1.4 Construction of phage-displayed peptide libraries

1.5 Phage panning

1.6 Fluorescence polarization (FP) assays

1.7 Surface plasmon resonance (SPR) assays

1.8 NMR characterization

1.9 Proteolytic stability of peptides

1.10 CD characterization

2. Supplementary Data

2.1 Sequences and mass spectral characterizations of the synthesized peptides (Table S1–S4)

2.2 Chromatograms of peptides (Figure S1–S8)

2.3 Characterization of disulfide pairing of 12 and 14 using trypsin digestion (Figure S9–S10)

2.4 Construction of phage-displayed peptide libraries (Figure S11–S12, Table S5)

2.5 Sequences obtained from screening of peptide libraries and NMR characterization of the oxidized peptides (Figure S13–S16, Table S6–S7)

2.6 Binding affinity of the oxidized peptides to target proteins (Figure S17–S23)

2.7 Proteolytic stability of peptides (Figure S24–S25)

2.8 CD spectra of the oxidized **15** and oxidized **16** (Figure S26)

2.8 Mass spectra of peptides (Figure S27)

1. Experimental Section

1.1 Materials and instruments

All the Fmoc-protected amino acids and Rink amide MBHA resin used for peptide synthesis were supplied by GL Biochem (Shanghai, China). Glutathione oxidized (GSSG), glutathione reduced (GSH) and tris (2-carboxyethyl) phosphine hydrochloride (TCEP) were bought from Energy Chemical (Shanghai, China). $Na_2HPO_4 \cdot 12H_2O$, $NaH_2PO_4 \cdot 2H_2O$, meta-phosphoric acid (HPO_3) and trypsin were purchased from Sigma-Aldrich (Beijing, China). Acetonitrile (ACN), trifluoroacetic acid (TFA), sodium acetate trihydrate ($NaAc \cdot 3H_2O$), EDTA disodium salt dihydrate, Sodium dodecyl sulfate (SDS), and all the chemicals used to prepare 1×TAE (Tris-HCl, EDTA disodium salt dihydrate, acetic acid), 1×TE (Tris-HCl, EDTA disodium salt dihydrate), neutralization buffer (Tris-HCl), elution buffer (glycine), 2YT medium (tryptone, yeast extract, NaCl), PEG/NaCl (PEG-6000, NaCl), binding buffer (Tris-HCl, NaCl, $MgCl_2$, $CaCl_2$), washing buffer (Tris-HCl, NaCl, $MgCl_2$, $CaCl_2$, Tween-20) and blocking buffer (Tris-HCl, NaCl, $MgCl_2$, $CaCl_2$, Tween-20 and BSA) were purchased from Sigma-Aldrich (Beijing, China), Sangon Biotech (Shanghai, China) or Sinopharm Chemical Reagent (Beijing, China). Dimethyl sulfoxide (DMSO), acetic anhydride (Ac_2O), N,N-dimethylformamide (DMF), piperidine, and phenol were bought from Sinopharm Chemical Reagent (Beijing, China). Klenow fragment, *Sfi* I, *Not* I, T4 DNA Ligase, and 20 bp DNA Ladder were bought from Takara (Beijing, China). 20×PBS (Na_2HPO_4, KH_2PO_4, NaCl, KCl), DL5000 DNA Marker, ampicillin (Amp), agar, ammonium persulfate and 4S Red Plus were bought from Sangon Biotech (Shanghai, China). EZ-Link™ Sulfo-NHS-LC-Biotin, Dynabeads™ M-280 Tosylactivated, Dynabeads™ M-280 Streptavidin and NeutrAvidin protein were purchased from Thermo Fisher Scientific (Shanghai, China). Streptavidin was purchased from Biosynthesis Biotechnology (Beijing, China). Acetonitrile-D3 was purchased from Cambridge Isotope Laboratories (Beijing, China). Deuterium oxide (D_2O) was purchased from Energy Chemical (Shanghai, China). TG1 *E.coli* strain was purchased from Beyotime (Shanghai, China).

All peptides were synthesized using solid-phase peptide synthesis by a CEM Discover Liberty BLUE microwave-assisted peptide synthesizer. High performance liquid chromatography (HPLC, SHIMADZU) equipped with a quaternary solvent manager (QSM), an AQUITY® PDA detector and a column manager was used for analysis of peptides. Reaction progress was monitored by HPLC (a flow rate of 1.0 mL·min^{-1} flow rate of H_2O (+0.1% TFA) and ACN (+0.1% TFA); isocratic with 10% ACN (+0.1% TFA) for 5.0 min followed by a linear gradient of 10% to 85% ACN (+0.1% TFA) over 30 min). A HITACHI U-3900H UV/Vis spectrometer was used for the quantification of peptides. Surface Plasmon Resonance (SPR) assays were performed by Biacore T200. Fluorescence polarization was recorded by Tecan Infinite® 200 PRO Microplate Reader. Bruker impact II-TOF mass spectrometry, Bruker Esquire 3000 plus ion trap ESI mass spectrometry and Bruker autoflex max MALDI-TOF mass spectrometry were applied to identify the formed peptides. NMR experiments were recorded at 298 K on Bruker AVANCE III 600 MHz and 850 MHz equipped with a cryogenic triple-resonance probe. Fast Protein Liquid Chromatography (FPLC) equipped with a Hitrap™ Desalting column (GE Healthcare) was used to purify the biotinylated proteins.

1.2 Synthesis of peptides

All peptides are N-terminally acetylated and C-terminal amidated, which were synthesized at 0.05 mmol scale using the Fmoc solid-phase peptide synthesis (SPPS) on a CEM Liberty blue automated microwave peptide synthesizer (Table S1). Amino acids were coupled onto the MBHA resins using the standard coupling protocol. Peptides were cleaved from the resin and deprotected by treating with a TFA cleavage cocktail for 4 h at room temperature. Then, the cleaved peptides were precipitated in cold diethyl ether, and purified using a HPLC system. All peptides were purified to a purity of >95% before performing the oxidation (Figures S1 and S7).

1.3 Oxidation of peptides

Reduced peptides (>95% purity) isolated by HPLC were identified by mass spectrometry (Table S2). The HPLC-isolated peptides were lyophilized and re-dissolved in a mixture of water and acetonitrile (70%:30% vol/vol) as the stock solution for further use. Concentrations of the reduced

peptide stock solutions were determined by UV/Vis spectroscopy (ε_{Trp} = 5,502 cm^{-1} M^{-1} at 280 nm). In a typical experiment for peptide oxidation, the reduced peptide was reconstituted in an appropriate amount of phosphate buffer (100 mM, pH 7.4) containing 30% acetonitrile, 0.5 mM GSH and 0.5 mM GSSG to achieve a concentration of 50 μM. After about 4–6 h, the oxidation was complete, and the oxidized peptide was characterized by HPLC and mass spectrometry (Table S3). The Acm-protected peptides were oxidized in the redox buffer in the same way. After the HPLC-purification of the oxidized Acm-protected peptides, the peptide was lyophilized and reconstituted in pure water to achieve a definite concentration (determined by UV/Vis spectroscopy). Then, the peptide solution was diluted by addition of methanol with 0.1% TFA to a concentration of 50 μM, into which 10 eq. I dissolved in methanol was added dropwise. After ~1 h, the Acm was completely removed from the peptide, leading to the formation of the third disulfide bond in the peptide. The final Acm-deprotected product was analyzed using HPLC and mass spectrometry (Table S4). To characterize the disulfide pairing of oxidized **12** and **14**, the lyophilized powder (oxidized **12** and **14** isolated by HPLC) was dissolved in a phosphate buffer (100 mM, pH 6.0) containing 50 μg/mL trypsin for 4 h, and then the major digested peptide fragment isolated by HPLC (monitored at 280 nm) was identified using mass spectrometry (Figures S9 and S10).

1.4 Construction of phage-displayed peptide libraries

The peptide libraries were constructed based on a procedure reported previously (Table S5).[1] In brief, DNA libraries encoding random peptide sequences were digested by *Sfi* I (10 h, 50 °C) and *Not* I (10 h, 37 °C), which were recovered by gel purification. Then, the purified DNA fragments were

ligated with gel-purified *Sfi* I/*Not* I-digested vector pCantab 5E (ratio of insert and vector: 10/1). The ligation mixture was then transformed into *E. coli* TG1 competent cells. The cells were plated on 2 × YT/ampicillin agar plates and incubated at 37 °C for 12 h. The size of the phage libraries was determined by measuring the total number of colonies. The colonies on the plates were then scraped off the plates and propagated for phage production and purification. 20–30 phage clones were randomly picked up for sequencing to evaluate the quality of the phage libraries.

1.5 Phage panning

Proteins (MDM2 and Bcl-2; 5 μM) were biotinylated in phosphate buffer saline (PBS; pH 7.4) with the addition of Sulfo-NHS-LC-biotin (50 μM) for ~0.5 h at room temperature. After the reaction, the unreacted Sulfo-NHS-LC-biotin was removed using a desalt column on an AKTA pure system (running buffer: pH 7.4 PBS). The biotinylated proteins can then be immobilized on streptavidin-coated and neutravidin-coated magnetic beads for screening as described previously.[2,3] Briefly, 100 μL of streptavidin-coated magnetic beads was washed three times with binding buffer (150 mM NaCl, 1 mM $CaCl_2$, 10 mM Tris-HCl, 10 mM $MgCl_2$, pH 7.4) in a 1.5 mL microcentrifuge tube, which were then re-suspended with 100 μL binding buffer and distributed equally into two 1.5 mL microcentrifuge tubes. Then, the biotinylated protein (first round: 10 μg; second round: 5 μg; third round: 2 μg) was added into one of the two tubes; meanwhile, the same volume of 1 × PBS was added into the other one as a control. The two tubes were incubated on a slowly rotating vortex mixer for 15 min at room temperature. Then, the beads in the two tubes were washed three times with the binding buffer to remove the free proteins, which were then resuspended with 300 μL

binding buffer and 150 μL Blocking buffer (binding buffer containing 0.3% Tween-20 and 3% w/v BSA). The suspension of beads was incubated at room temperature for 2 h on a slowly rotating vortex mixer. Meanwhile, to a tube containing 3.0 mL of phages (>10^{12} t.u.) dissolved in binding buffer, 1.5 mL blocking buffer was added, and the solution was incubated at room temperature for 2 h on a slowly rotating vortex mixer. Then, the phages in solution were split equally into two tubes, into which the BSA-blocked beads with and without the immobilized proteins were added, respectively. After 30 min incubation at room temperature, the unbound phages in the supernatant were removed and the beads were washed for nine times with washing buffer (binding buffer containing 0.1% Tween-20) and twice with binding buffer. During the washing steps, the tubes were replaced at least three time with new ones to avoid the non-specific adsorption of phages. After the last washing step, the beads were resuspended with 200 μL elution buffer (50 mM glycine, pH 2.2) and incubated at room temperature for 5 min. Then, the beads were magnetically precipitated and the supernatant was transferred to a new microcentrifuge tube containing 50 μL neutralization buffer (1 M Tris-Cl, pH 8.9). The eluted phages from both the experimental and control group were diluted to infect exponentially growing TG1 cells to quantify the phage titer, and the eluted phages from the experimental group were propagated for the production of phages used for next-round panning. After three rounds of panning as described above, 20–30 phage clones were randomly picked for sequencing. Note that streptavidin-coated and neutravidin-coated magnetic beads were used to immobilize biotinylated proteins alternatively in each round of selection to prevent the enrichment of streptavidin and neutravidin binders during the panning.[2,4] For the library screening against streptavidin, streptavidin-coated magnetic beads were directly used for panning.

1.6 Fluorescence polarization (FP) assays

FP assays were performed on 96-well flat-bottom OptiPlate black plates using a Tecan Infinite® 200 PRO Microplate Reader. To examine the binding affinity of oxidized **16** to Bcl-2, a fluorescein-labelled **16** (FITC-βAla-**16**) was synthesized and purified to a purity of >95% using HPLC. Fluorescein-labelled **16** was first dissolved in DMSO and then diluted with PBS (pH 7.4) for further use. Sumo-Bcl-2 was obtained from an *E. coli* cell expression system described previously.[5] For the FP assay, 20 nM of FITC-βAla-**16** was incubated with Sumo-Bcl-2 (0–1.2 μM) in PBS (pH 7.4) on a 96-well plate at room temperature for 10 min, and fluorescence anisotropies were recorded. The K_D value of the binding between FITC-βAla-**16** and Bcl-2 can then be obtained by fitting the data with the single-site binding model. For the FP competition assay, 20 nM of FITC-βAla-**16** and 400 nM of Sumo-Bcl-2 were incubated with oxidized **16** (0–10 μM) under the same conditions as described above, and fluorescence anisotropies were recorded. The K_i value of the binding between oxidized **16** and Bcl-2 can then be obtained using a data-fitting procedure reported previously.[5] The binding of peptides to MDM2 was evaluated using the same procedure described above, which has been described in our previous reports.[1,6]

1.7 Surface plasmon resonance (SPR) assays

SPR assays were performed using a Biacore T200. Target proteins (Sumo-Bcl-2 and streptavidin) were immobilized on chip surface using a procedure described previously.[5] Briefly, Flow cell Fc4 was activated first with a mixture of NHS and EDC, and then Sumo-Bcl-2 (or streptavidin) dissolved in 10 mM acetate buffer (pH 5.0) flowed through the cell for protein coupling

to reach a target response of 1000 RU. Flow cell Fc3 that was not immobilized with proteins was used as the reference channel. Serially diluted samples were then passed over the flow cells at a flow rate of 30 μL/min (160, 320, 640, 1280, 2560, 640 nM for oxidized **15**, **16**, and **17**; 20, 40, 80, 160, 320, 80 nM for oxidized **11**, **15**, and **16**; 5, 10, 20, 40, 80 nM for oxidized **17**). Kinetics data were then analyzed using a 1:1 binding model and local fit to obtain kinetic rate constant and dissociation constant.

1.8 NMR characterization

NMR samples containing 0.5 mM of oxidized **15** and oxidized **16** were prepared in 50% perdeuterated acetonitrile and 50% H_2O. NMR experiments were recorded at 298 K on Bruker AVANCE III 600 MHz and 850 MHz equipped with a cryogenic triple-resonance probe for oxidized **15** and **16**, respectively. Two dimensional (2D) ^{1}H, ^{15}N/^{13}C HSQC spectra were recorded to obtain chemical shifts of ^{13}C/^{15}N in backbone and side-chains. 2D ^{1}H-^{1}H TOCSY (80 ms) and 2D ^{1}H-^{1}H COSY spectra were acquired to assign NMR signals of the peptide, and 2D ^{1}H-^{1}H NOESY spectra with a mixing time of 300 ms were measured to obtain ^{1}H-^{1}H distance constraints. The NMR data were processed using NMRPipe/NMRDraw and analyzed using NMRFAM-SPARKY.[7,8] About 95% of all NOE cross-peaks were assigned manually. Prediction of backbone dihedral angle restraints of the oxidized **16** was performed using TALOS-N based on chemical shifts of backbone resonances. Structural calculations were carried out with ARIA2.3.2 and CNS1.21.[9-11] An ensemble of 10 lowest-energy structures was generated from a total of 150 structures. The structures were visualized in PyMol and the quality of structures was analyzed using PROCHECK.[12]

1.9 Proteolytic stability of peptides

100 μM **15**-Ala, **16**-Ala, oxidized **15**, and oxidized **16** were respectively treated with phosphate buffer (pH = 7.4, 100 mM) containing chymotrypsin at 37 °C. The chymotrypsin concentration used for the digestion of **15**-Ala and oxidized **15** is 0.3 μg/mL. The chymotrypsin concentration used for the digestion of **16**-Ala and oxidized **16** is 5.0 μg/mL. At predefined times, 50 μL aliquots were taken with a pipette, quenched with 70 μL 10% HPO_3, and analyzed by HPLC.

1.10 CD characterization

Circular dichroism (CD) spectra were measured using a 1.0 mm path length cuvette at room temperature (25 °C). All measured peptides were dissolved in water to reach a concentration of 50 μM. The baseline signal (pure aqueous solution) were subtracted from each spectrum. CD spectra were recorded in a wavelength range of 190–260 nm with a bandwidth of 2.0 nm, a date pitch of 1.0 nm, a response time of 8.0 s, and a scanning speed of 50 nm/min. The thermal denaturation CD spectra were recorded in a temperature range of 25–95 °C with a heating rate of 10 °C/min. For temperature melts, the change of ellipticity at 220 nm was monitored as temperature increased from 25 to 95 °C in an increment of 10 °C. Other experimental conditions were consistent with those of the general method.

2. Supplementary Data

2.1 Sequences and mass spectral characterizations of the synthesized peptides

Table S1. Sequences of the synthesized peptides

Name	Sequence
**1**	Ac-CGSGSGSGSGCPCCWPELCPWIRSC-NH_2
**1-Acm**	Ac-C(Acm)GSGSGSGSGCPCC(Acm)WPELCPWIRSC-NH_2
**2**	Ac-CGSGSGSGSGCPCICPELCPWIRSC-NH_2
**2-Acm**	Ac-C(Acm)GSGSGSGSGCPCIC(Acm)PELCPWIRSC-NH_2
**3**	Ac-CGSGSGSGSGCPCIWPCLCPWIRSC-NH_2
**3-Acm**	Ac-C(Acm)GSGSGSGSGCPCIWPC(Acm)LCPWIRSC-NH_2
**4**	Ac-CGSGSGSGSGCPCIWPECCPWIRSC-NH_2
**4-Acm**	Ac-C(Acm)GSGSGSGSGCPCIWPEC(Acm)CPWIRSC-NH_2
**5**	Ac-CGSGSGSGSGCPCIWPELCPCIRSC-NH_2
**5-Acm**	Ac-C(Acm)GSGSGSGSGCPCIWPELCPC(Acm)IRSC-NH_2
**6**	Ac-CGSGSGSGSGCPCIWPELCPWCRSC-NH_2
**6-Acm**	Ac-C(Acm)GSGSGSGSGCPCIWPELCPWC(Acm)RSC-NH_2
**7**	Ac-CGSGSGSGSGCPCIWPELCPWICSC-NH_2
**7-Acm**	Ac-C(Acm)GSGSGSGSGCPCIWPELCPWIC(Acm)SC-NH_2
**8**	Ac-CGSGSGSGSGCPCIWPELCPWIRCC-NH_2
**8-Acm**	Ac-C(Acm)GSGSGSGSGCPCIWPELCPWIRC(Acm)C-NH_2
**9**	Ac-CGSGSGCPCIWPELCPWIRCC-NH_2
**9-Acm**	Ac-C(Acm)GSGSGCPCIWPELCPWIRC(Acm) C-NH_2
**10**	Ac-CGSGCPCIWPELCPWIRCC-NH_2
**10-Acm**	Ac-C(Acm)GSGCPCIWPELCPWIRC(Acm) C-NH_2
**11**	Ac-CGSGSGSGCGCPCIWPELCPWIRSC-NH_2

**11-Acm**	Ac-C(Acm)GSGSGSGC(Acm)GCPCIWPELCPWIRSC-NH_2
**12**	Ac-CGSGSGSCGKGCPCIWPELCPWIRSC-NH_2
**13**	Ac-CGSGSGSGSGCPCIWPELCPWIRSCGGC-NH_2
**13-Acm**	Ac-C(Acm)GSGSGSGSGCPCIWPELCPWIRSCGGC(Acm)-NH_2
**14**	Ac-GCPCIWPELCPWIRSCGKGCGSGSGSC-NH_2
**15**	Ac-GCEKATESESPCPCIFPELCPWIVCC-NH_2
**15-Ala**	Ac-GAEKATESESPAPAIFPELAPWIVAA-NH_2
**16**	Ac-GCMILLDTDIWCPCSHPYACPENICC-NH_2
**16-Ala**	Ac-GAMILLDTDIWAPASHPYAAPENIAA-NH_2
**FITC-βAla-16**	FITC-(β-Ala)-GCMILLDTDIWCPCSHPYACPENICC-NH_2
**17**	Ac-GCGSDEACFCPCSHPENCPMAQHC-NH_2
**17-Acm**	Ac-G C(Acm)GSDEAC(Acm)FCPCSHPENCPMAQHC-NH_2

Table S2. MS characterization of reduced peptides

Name	m/z expected	m/z found	Molecular formula
**1**	1287.5029 ([M+2H]$^{2+}$)	1287.4804	$C_{105}H_{157}N_{31}O_{33}S_6$
**1-Acm**	1358.5400 ([M+2H]$^{2+}$)	1358.5229	$C_{111}H_{167}N_{33}O_{35}S_6$
**2**	1251.0052 ([M+2H]$^{2+}$)	1250.9809	$C_{100}H_{158}N_{30}O_{33}S_6$
**2-Acm**	1322.0423 ([M+2H]$^{2+}$)	1322.0223	$C_{106}H_{168}N_{32}O_{35}S_6$
**3**	1279.5236 ([M+2H]$^{2+}$)	1279.4994	$C_{106}H_{161}N_{31}O_{31}S_6$
**3-Acm**	1350.5607 ([M+2H]$^{2+}$)	1350.5439	$C_{112}H_{171}N_{33}O_{33}S_6$
**4**	1287.5029 ([M+2H]$^{2+}$)	1287.4911	$C_{105}H_{157}N_{31}O_{33}S_6$
**4-Acm**	1358.5400 ([M+2H]$^{2+}$)	1358.5289	$C_{111}H_{167}N_{33}O_{35}S_6$
**5**	1251.0052 ([M+2H]$^{2+}$)	1250.9823	$C_{100}H_{158}N_{30}O_{33}S_6$
**5-Acm**	1322.0423 ([M+2H]$^{2+}$)	1322.0474	$C_{106}H_{168}N_{32}O_{35}S_6$
**6**	1287.5029 ([M+2H]$^{2+}$)	1287.4911	$C_{105}H_{157}N_{31}O_{33}S_6$
**6-Acm**	1358.5400 ([M+2H]$^{2+}$)	1358.5389	$C_{111}H_{167}N_{33}O_{35}S_6$
**7**	1265.9944 ([M+2H]$^{2+}$)	1265.9326	$C_{105}H_{156}N_{28}O_{33}S_6$
**7-Acm**	1337.0315 ([M+2H]$^{2+}$)	1336.9771	$C_{111}H_{166}N_{30}O_{35}S_6$
**8**	1300.5289 ([M+2H]$^{2+}$)	1300.6187	$C_{108}H_{163}N_{31}O_{32}S_6$
**8-Acm**	1371.5660 ([M+2H]$^{2+}$)	1371.5506	$C_{114}H_{173}N_{33}O_{34}S_6$
**9**	1156.4754 ([M+2H]$^{2+}$)	1156.4789	$C_{98}H_{147}N_{27}O_{26}S_6$
**9-Acm**	1227.5125 ([M+2H]$^{2+}$)	1227.4617	$C_{104}H_{157}N_{29}O_{28}S_6$
**10**	1084.4486 ([M+2H]$^{2+}$)	1084.4396	$C_{93}H_{139}N_{25}O_{23}S_6$
**10-Acm**	1155.4858 ([M+2H]$^{2+}$)	1155.4882	$C_{99}H_{149}N_{27}O_{25}S_6$
**11**	1300.5289 ([M+2H]$^{2+}$)	1300.6557	$C_{108}H_{163}N_{31}O_{32}S_6$
**11-Acm**	1371.5660 ([M+2H]$^{2+}$)	1371.6973	$C_{114}H_{173}N_{33}O_{34}S_6$
**12**	2728.1454 ([M+H]$^{+}$)	2728.169	$C_{114}H_{175}N_{33}O_{33}S_6$
**13**	1401.0664 ([M+2H]$^{2+}$)	1401.2576	$C_{115}H_{174}N_{34}O_{36}S_6$

**13-Acm**	1472.6039 ([M+2H]$^{2+}$)	1472.8245	$C_{121}H_{184}N_{36}O_{38}S_6$
**14**	2785.1669 ([M+H]$^{+}$)	2785.292	$C_{116}H_{178}N_{34}O_{34}S_6$
**15**	1444.1128 ([M+2H]$^{2+}$)	1444.0485	$C_{124}H_{189}N_{29}O_{38}S_6$
**15-Ala**	2716.3670 ([M+Na]$^{+}$)	2716.5180	$C_{124}H_{189}N_{29}O_{38}$
**16**	1472.1044 ([M+2H]$^{2+}$)	1472.0147	$C_{125}H_{189}N_{31}O_{37}S_7$
**16-Ala**	2750.3683 ([M+H]$^{+}$)	2750.139	$C_{125}H_{189}N_{31}O_{37}S$
**FITC-βAla-16**	3363.2316 ([M+Na]$^{+}$)	3362.659	$C_{147}H_{201}N_{33}O_{41}S_8$
**17**	2567.8820 ([M+H]$^{+}$)	2567.474	$C_{100}H_{147}N_{31}O_{35}S_7$
**17-Acm**	2708.9536 ([M+H]$^{+}$)	2709.111	$C_{106}H_{157}N_{33}O_{37}S_7$

Table S3. MS characterization of oxidized peptides

Name	m/z expected	m/z found	Molecular formula
**1**	1284.4794 ([M+2H]$^{2+}$)	1284.3791	$C_{105}H_{151}N_{31}O_{33}S_6$
**1-Acm**	1356.5243 ([M+2H]$^{2+}$)	1356.4338	$C_{111}H_{163}N_{33}O_{35}S_6$
**2**	1247.9817 ([M+2H]$^{2+}$)	1247.8865	$C_{100}H_{152}N_{30}O_{33}S_6$
**2-Acm**	1320.0267 ([M+2H]$^{2+}$)	1319.9352	$C_{106}H_{164}N_{32}O_{35}S_6$
**3**	1276.5001 ([M+2H]$^{2+}$)	1276.4056	$C_{106}H_{155}N_{31}O_{31}S_6$
**3-Acm**	1348.5451 ([M+2H]$^{2+}$)	1348.4583	$C_{112}H_{167}N_{33}O_{33}S_6$
**4**	1284.4794 ([M+2H]$^{2+}$)	1284.3811	$C_{105}H_{151}N_{31}O_{33}S_6$
**4-Acm**	1356.5243 ([M+2H]$^{2+}$)	1356.4200	$C_{111}H_{163}N_{33}O_{35}S_6$
**5**	1247.9817 ([M+2H]$^{2+}$)	1247.8832	$C_{100}H_{152}N_{30}O_{33}S_6$
**5-Acm**	1320.0267 ([M+2H]$^{2+}$)	1320.0356	$C_{106}H_{164}N_{32}O_{35}S_6$
**6**	1284.4794 ([M+2H]$^{2+}$)	1284.3877	$C_{105}H_{151}N_{31}O_{33}S_6$
**6-Acm**	1356.5243 ([M+2H]$^{2+}$)	1356.5320	$C_{111}H_{163}N_{33}O_{35}S_6$
**7**	1262.9709 ([M+2H]$^{2+}$)	1262.9076	$C_{105}H_{150}N_{28}O_{33}S_6$
**7-Acm**	1335.0158 ([M+2H]$^{2+}$)	1334.9560	$C_{111}H_{162}N_{30}O_{35}S_6$
**8**	1297.5054 ([M+2H]$^{2+}$)	1297.7093	$C_{108}H_{157}N_{31}O_{32}S_6$
**8-Acm**	1369.5503 ([M+2H]$^{2+}$)	1369.5190	$C_{114}H_{169}N_{33}O_{34}S_6$
**9**	1153.4519 ([M+2H]$^{2+}$)	1153.4468	$C_{98}H_{141}N_{27}O_{26}S_6$
**9-Acm**	1225.4969 ([M+2H]$^{2+}$)	1225.4573	$C_{104}H_{153}N_{29}O_{28}S_6$
**10**	1081.4252 ([M+2H]$^{2+}$)	1081.4202	$C_{93}H_{133}N_{25}O_{23}S_6$
**10-Acm**	1153.4701 ([M+2H]$^{2+}$)	1153.4712	$C_{99}H_{145}N_{27}O_{25}S_6$
**11**	1297.5054 ([M+2H]$^{2+}$)	1297.7083	$C_{108}H_{157}N_{31}O_{32}S_6$
**11-Acm**	1370.0507 ([M+2H]$^{2+}$)	1370.2456	$C_{114}H_{169}N_{33}O_{34}S_6$
**12**	2722.0985 ([M+H]$^{+}$)	2722.203	$C_{114}H_{169}N_{33}O_{33}S_6$
**13**	1398.0429 ([M+2H]$^{2+}$)	1398.2554	$C_{115}H_{168}N_{34}O_{36}S_6$

**13-Acm**	1470.5883 ($[M+2H]^{2+}$)	1470.8027	$C_{121}H_{180}N_{36}O_{38}S_6$
**14**	2779.1200 ($[M+H]^{+}$)	2779.889	$C_{116}H_{172}N_{34}O_{34}S_6$
**15**	1440.5889 ($[M+2H]^{2+}$)	1441.0	$C_{124}H_{183}N_{29}O_{38}S_6$
**16**	1468.5805 ($[M+2H]^{2+}$)	1468.5018	$C_{125}H_{183}N_{31}O_{37}S_7$
**FITC-βAla-16**	3357.1846 ($[M+Na]^{+}$)	3356.454	$C_{147}H_{195}N_{33}O_{41}S_8$
**17**	2561.8351 ($[M+H]^{+}$)	2562.378	$C_{100}H_{141}N_{31}O_{35}S_7$
**17-Acm**	2704.9223 ($[M+H]^{+}$)	2705.077	$C_{106}H_{153}N_{33}O_{37}S_7$

Table S4. MS characterization of Acm-deprotected peptides

Name	m/z expected	m/z found	Molecular formula
**1-Acm**	1284.4794 ([M+2H]$^{2+}$)	1284.3796	$C_{105}H_{151}N_{31}O_{33}S_6$
**2-Acm**	1247.9817 ([M+2H]$^{2+}$)	1247.8749	$C_{100}H_{152}N_{30}O_{33}S_6$
**3-Acm**	1276.5001 ([M+2H]$^{2+}$)	1276.4077	$C_{106}H_{155}N_{31}O_{31}S_6$
**4-Acm**	1284.4794 ([M+2H]$^{2+}$)	1284.3839	$C_{105}H_{151}N_{31}O_{33}S_6$
**5-Acm**	1247.9817 ([M+2H]$^{2+}$)	1247.9731	$C_{100}H_{152}N_{30}O_{33}S_6$
**6-Acm**	1284.4794 ([M+2H]$^{2+}$)	1284.4726	$C_{105}H_{151}N_{31}O_{33}S_6$
**7-Acm**	1262.9709 ([M+2H]$^{2+}$)	1262.9082	$C_{105}H_{150}N_{28}O_{33}S_6$
**8-Acm**	1297.5054 ([M+2H]$^{2+}$)	1297.5357	$C_{108}H_{157}N_{31}O_{32}S_6$
**9-Acm**	1153.4519 ([M+2H]$^{2+}$)	1153.4463	$C_{98}H_{141}N_{27}O_{26}S_6$
**10-Acm**	1081.4252 ([M+2H]$^{2+}$)	1081.3791	$C_{93}H_{133}N_{25}O_{23}S_6$
**11-Acm**	1297.5054 ([M+2H]$^{2+}$)	1297.7097	$C_{108}H_{157}N_{31}O_{32}S_6$
**13-Acm**	1398.0429 ([M+2H]$^{2+}$)	1398.2589	$C_{115}H_{168}N_{34}O_{36}S_6$
**17-Acm**	2561.8351 ([M+H]$^{+}$)	2562.348	$C_{100}H_{141}N_{31}O_{35}S_7$

2.2 Chromatograms of peptides

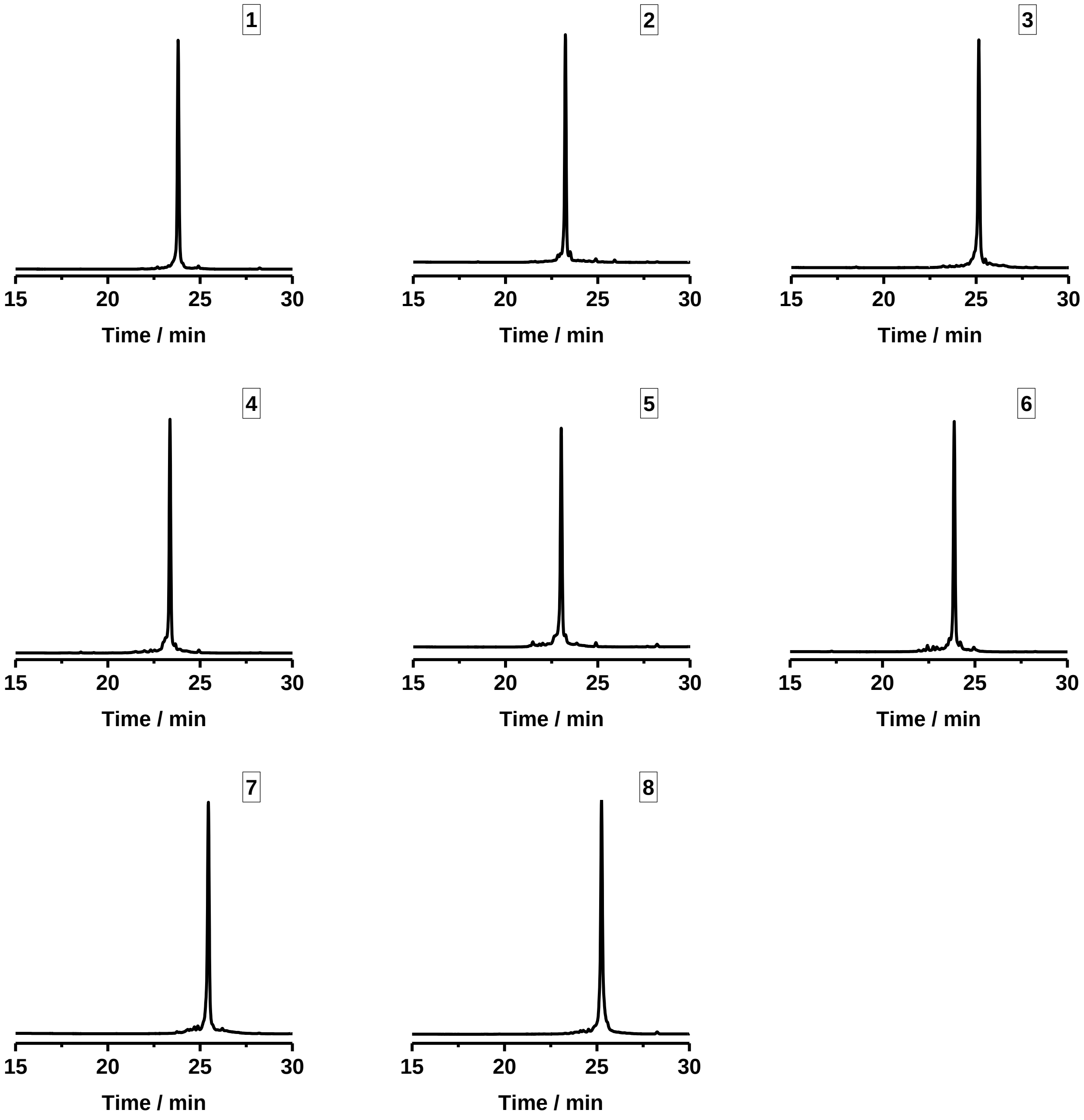

Figure S1. Chromatograms of peptides **1–8**.

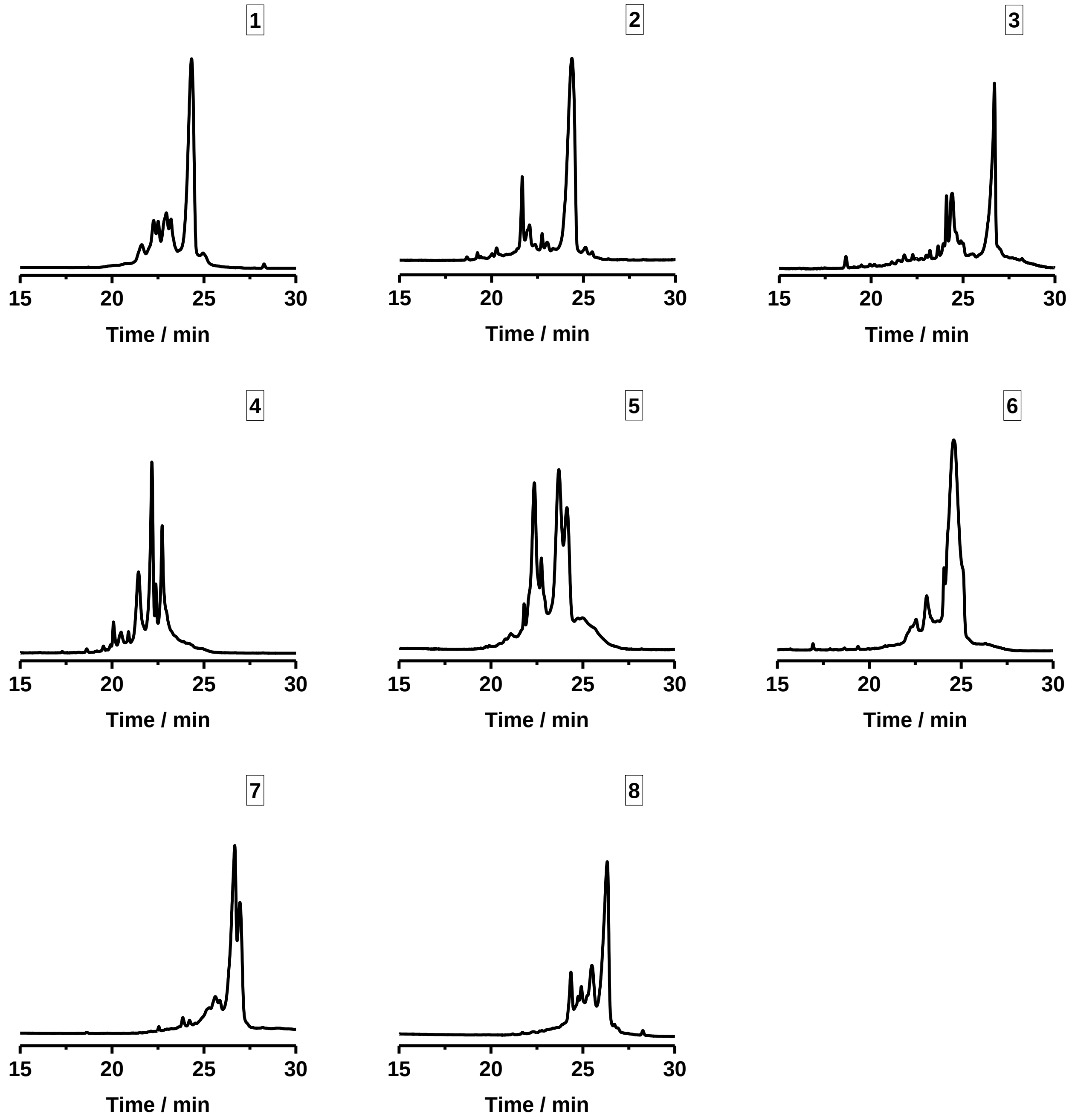

Figure S2. Chromatograms of the products formed after the oxidation of **1–8**.

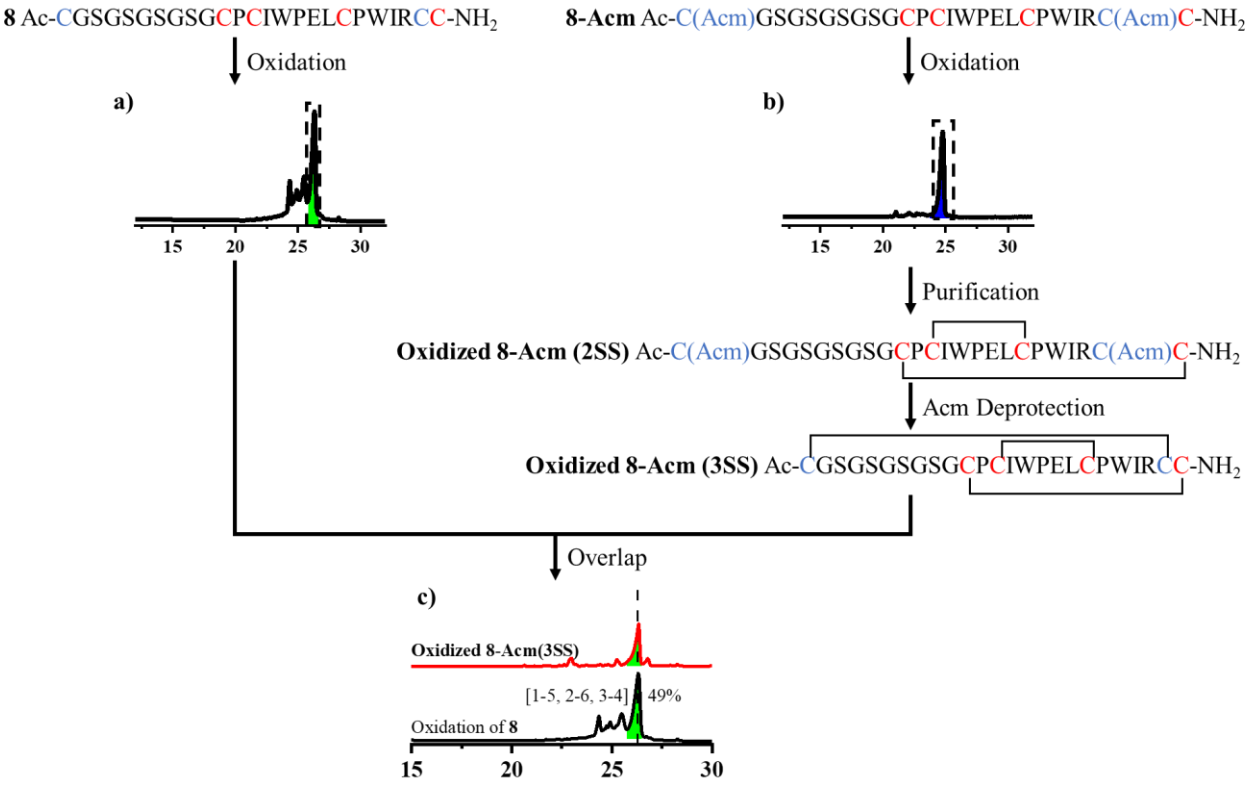

Figure S3. Analysis of disulfide pairing in peptide **8** through orthogonal protecting group strategies. a) Chromatogram of the products formed after the oxidation of **8**. b) Chromatograms of the products formed after the oxidation of **8-Acm**. c) Chromatogram of the products formed from the oxidative folding of **8** (black line) and chromatogram of the products formed from the Acm-deprotection of the oxidized **8-Acm** (red line).

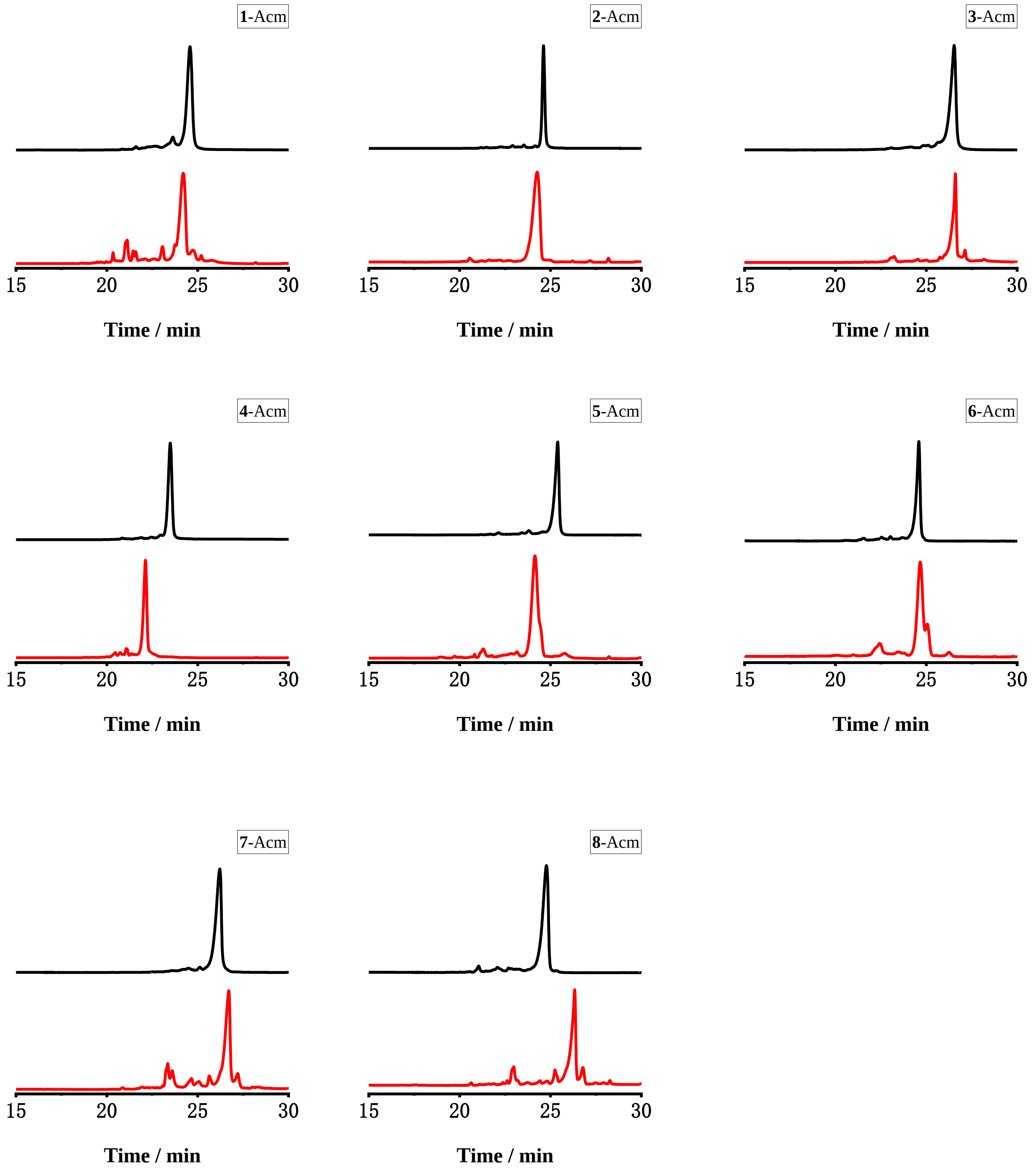

Figure S4. Chromatograms of products formed after the oxidation of **1-Acm~8-Acm** (black line) and chromatograms of the products formed after the Acm-deprotection of the oxidized **1-Acm~8-Acm** (red line).

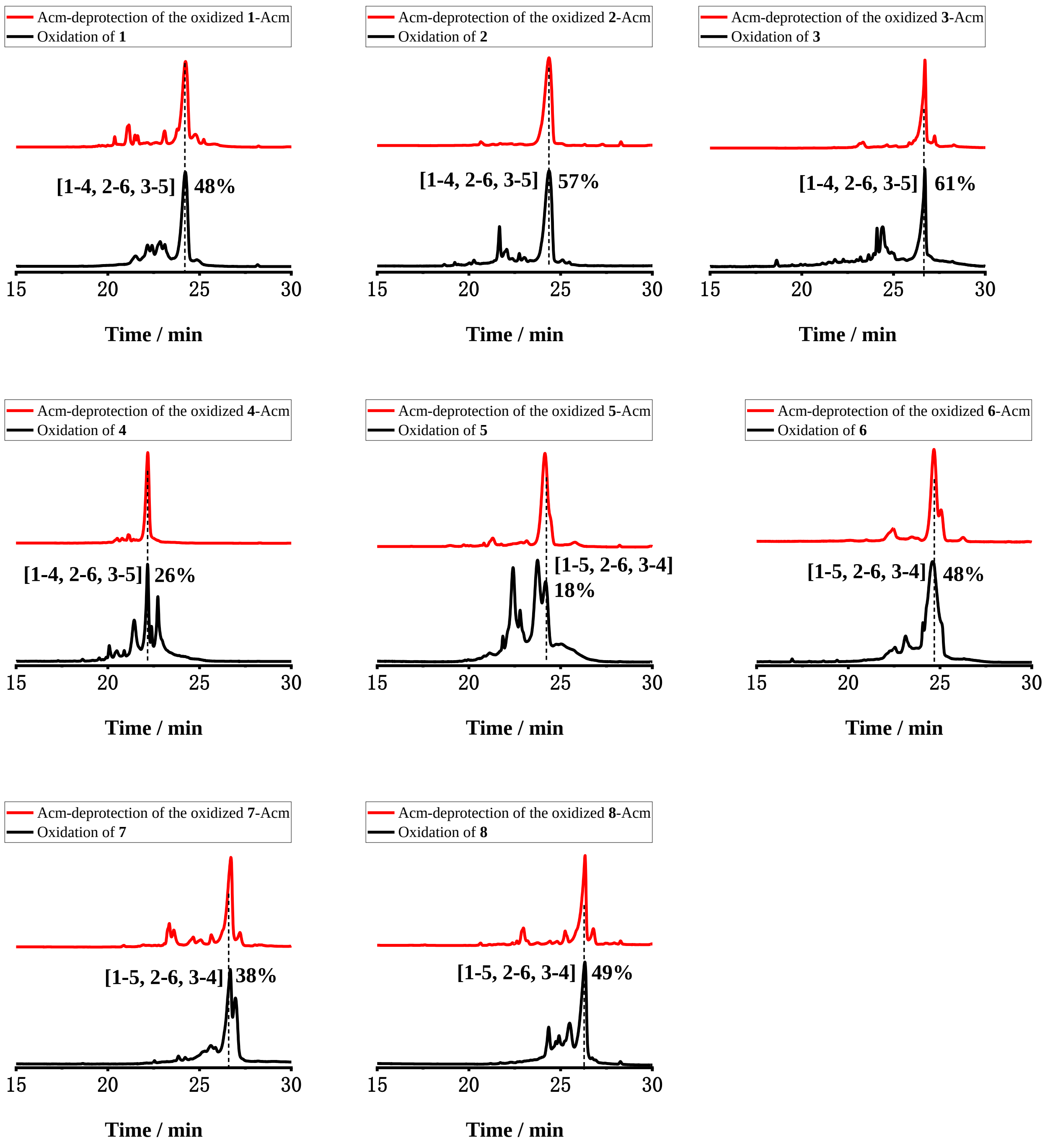

Figure S5. Chromatograms of products formed after the oxidation of **1–8** (black line) and chromatograms of the products formed after the Acm-deprotection of the oxidized **1-Acm~8-Acm** (red line).

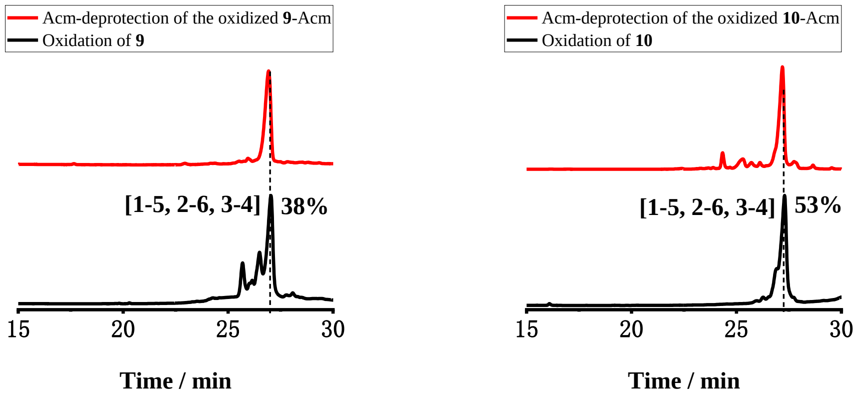

Figure S6. Chromatograms of the products formed after the oxidation of **9** and **10** (black line) and chromatograms of the products formed after the Acm-deprotection of the oxidized **9-Acm** and oxidized **10-Acm** (red line).

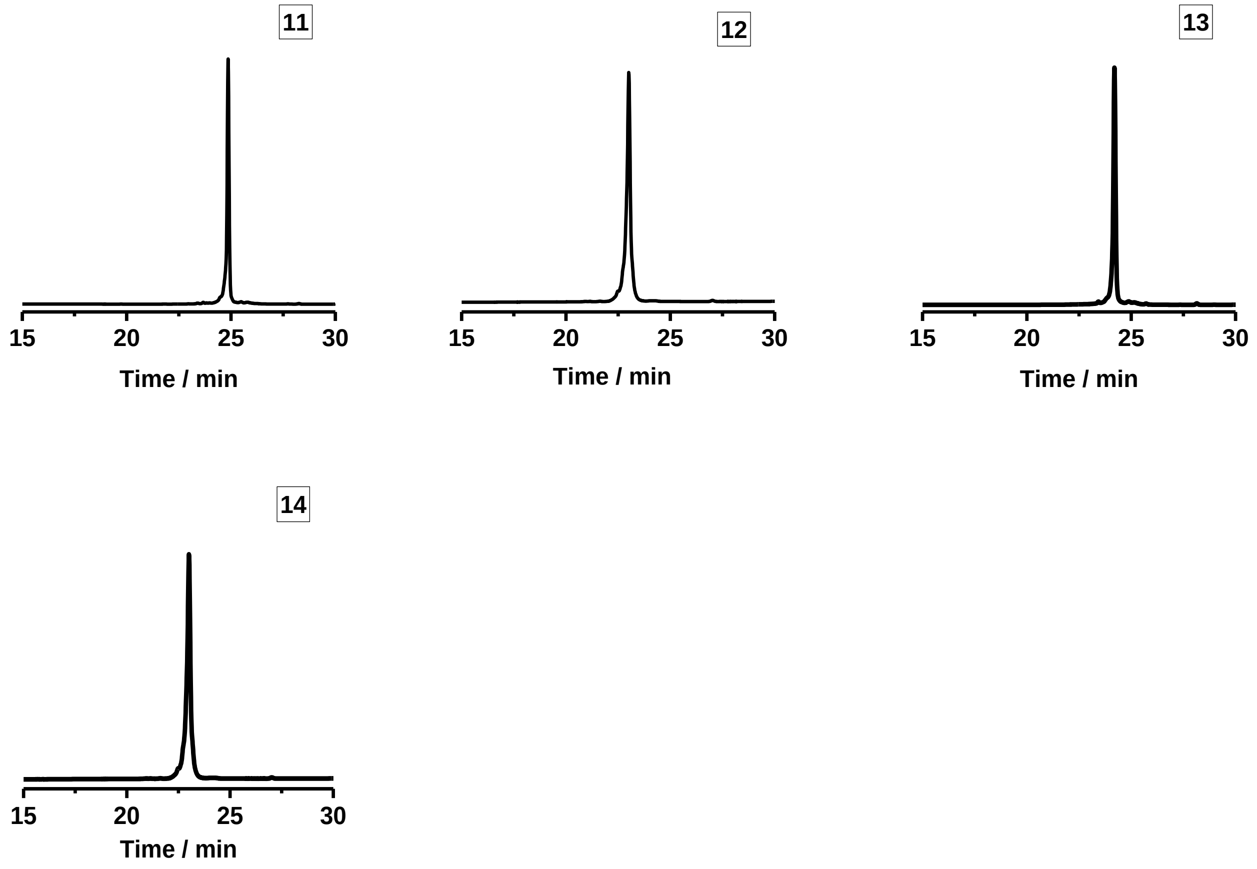

Figure S7. Chromatograms of peptides **11–14**.

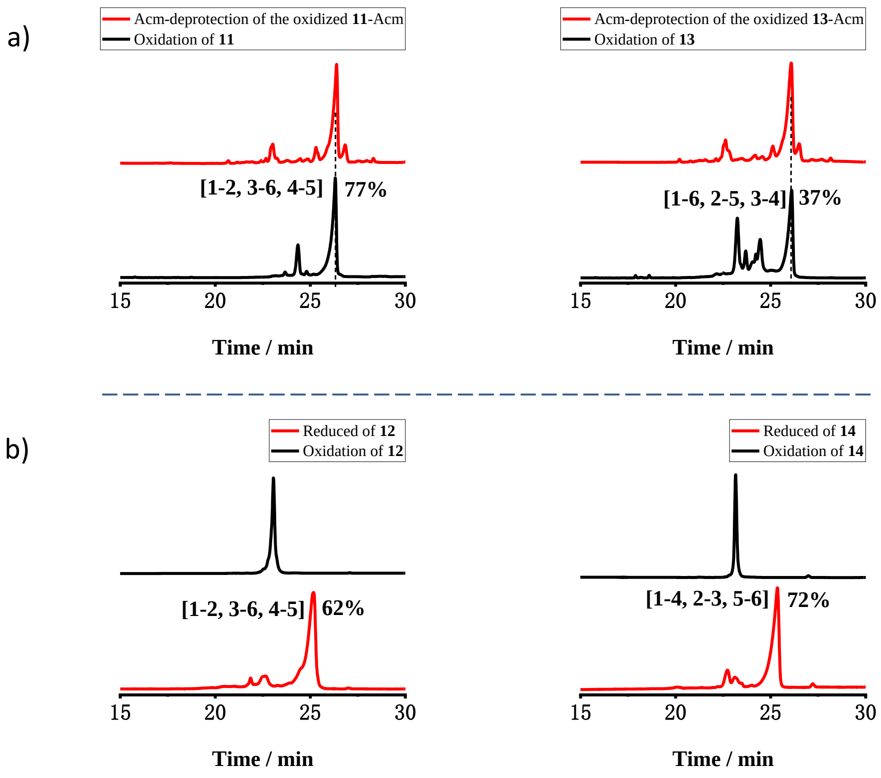

Figure S8. a) Chromatograms of the products formed after the oxidation of **11** and **13** (black line) and chromatograms of the products formed after the Acm-deprotection of oxidized **11-Acm** and oxidized **13-Acm** (red line). b) Chromatograms of **12** and **14** (black line) and chromatograms of the products formed after the oxidation of **12** and **14** (red line).

2.3 Characterization of disulfide pairing of 12 and 14 using trypsin digestion

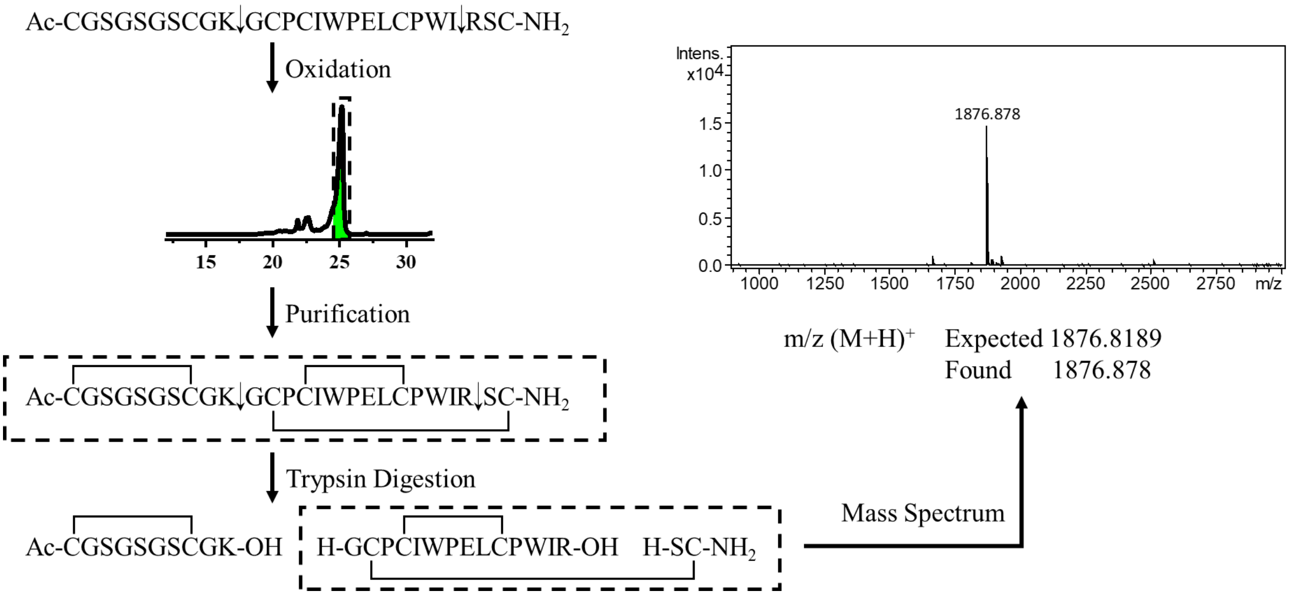

Figure S9. Tryptic digestion analysis of the oxidized **12**. ↓ denotes the cleavage site of trypsin.

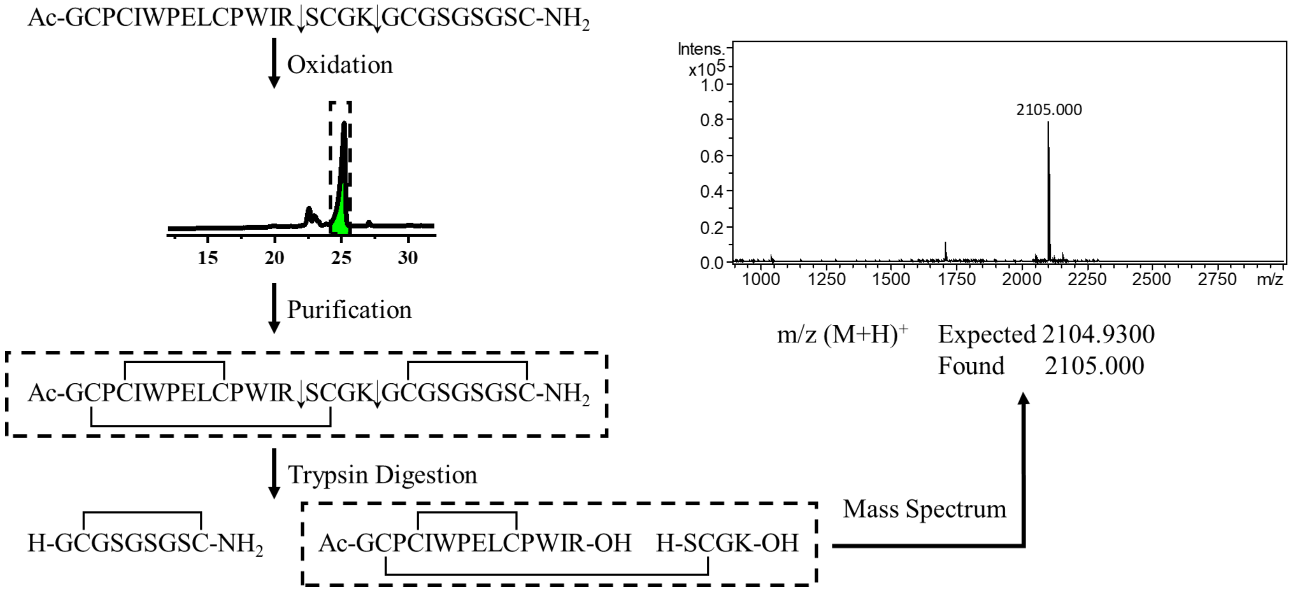

Figure S10. Tryptic digestion analysis of the oxidized **14**. ↓ denotes the cleavage site of trypsin.

2.4 Construction of phage-displayed peptide libraries

We selected **8** and **11** as model scaffolds to construct phage-displayed peptide libraries.

Extension primer 5'CACCGGCGCACCTTGCGGCCGC3'

Library oligonucleotide
5'TCGCggcccagccggccATGCAGGTTGCnnknnknnknnknnknnknnknnknnkTGCCCCTGCnnknnkCCCn
nknnkTGCCCCnnknnknnkTGCTGCgcggccgcAAGGTGCGCCGGTG3'

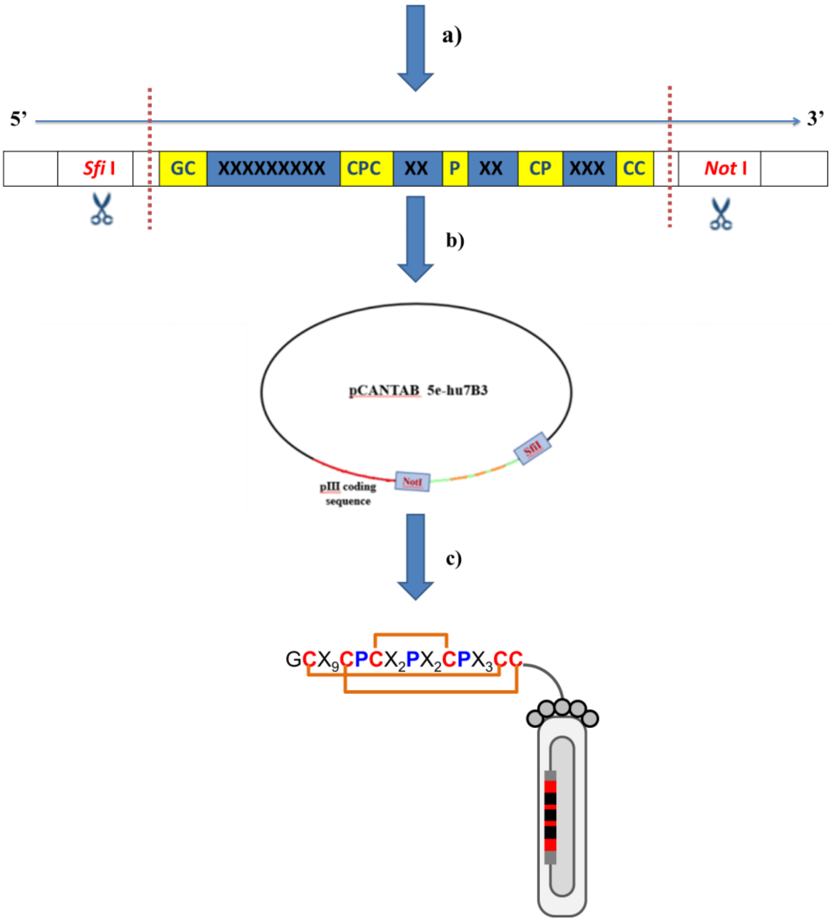

Figure S11. Design of phage-displayed disulfide-rich peptide library (taking **8**-X_{16} as an example).

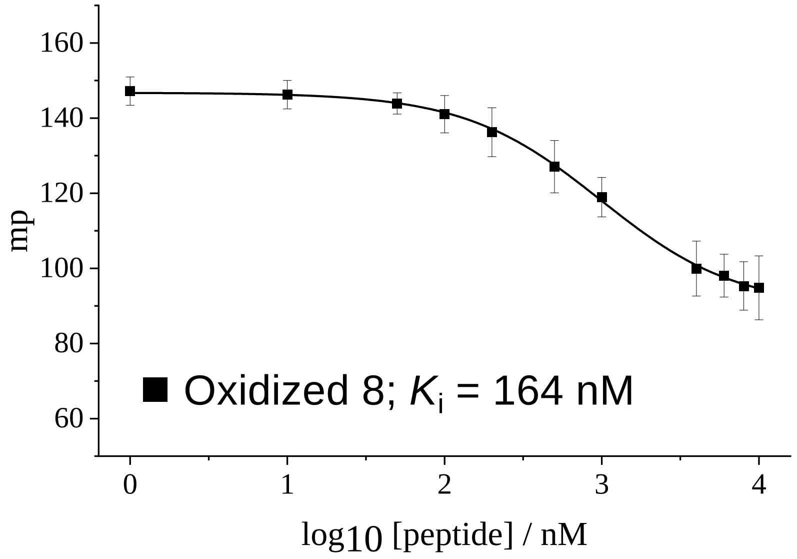

Figure S12. A fluorescence polarization competition assay showing the binding of oxidized **8** to MDM2.

Table S5. Four phage-displayed DRP libraries

Phage library	The sequence of phage library	Phage library capacity
**8**-X_5	$GCX_5CPCIWPELCPWIRCC$	4.75×10^8
**8**-X_9	$GCX_9CPCIWPELCPWIRCC$	1.4×10^9
**8**-X_{16}	$GCX_9CPCX_2P\ X_2CPX_3CC$	1.10×10^9
**11**-X_{14}	$GCX_5CXCPCX_2PX_2CPX_4C$	6.25×10^8

2.5 Sequences obtained from screening of peptide libraries and NMR characterization of the oxidized peptides

a)

Clones	Sequence
M-1	GC ERTDS CPCIWPELCPWIRCC
M-2	GC SDGES CPCIWPELCPWIRCC
M-3	GC EHGDS CPCIWPELCPWIRCC
M-4	GC NADES CPCIWPELCPWIRCC
M-5	GC NDECS CPCIWPELCPWIRCC
M-6	GC NESES CPCIWPELCPWIRCC
M-7	GC NTEEP CPCIWPELCPWIRCC
M-8	GC NADNL CPCIWPELCPWIRCC
M-9	GC NDIDE CPCIWPELCPWIRCC
M-10	GC QSEEL CPCIWPELCPWIRCC
M-11	GC QESDG CPCIWPELCPWIRCC
M-12	GC QEDEV CPCIWPELCPWIRCC
M-13	GC QERPA CPCIWPELCPWIRCC
M-14	GC QAPGE CPCIWPELCPWIRCC
M-15	GC TGSDE CPCIWPELCPWIRCC
M-16	GC TYGSE CPCIWPELCPWIRCC
M-17	GC PSKVE CPCIWPELCPWIRCC
M-18	GC LRAEE CPCIWPELCPWIRCC
M-19	GC WDERE CPCIWPELCPWIRCC
M-20	GC DGSKE CPCIWPELCPWIRCC
M-21	GC CQQAE CPCIWPELCPWIRCC
M-22	GC GAYTE CPCIWPELCPWIRCC
M-23	GC GEETE CPCIWPELCPWIRCC
M-24	GC SEEDP CPCIWPELCPWIRCC
M-25	GC MEPSE CPCIWPELCPWIRCC
M-26	GC ETPDE CPCIWPELCPWIRCC
M-27	GC YKDEA CPCIWPELCPWIRCC

b)

Clone	Sequence
M-1	GC NLKLPEDSE CPCIWPELCPWIRCC
M-2	GC SSYIMLCIH CPCIWPELCPWIRCC
M-3	GC RYDREHLSE CPCIWPELCPWIRCC
M-4	GC DNNLDSSLE CPCIWPELCPWIRCC
M-5	GC SESVAFSYH CPCIWPELCPWIRCC
M-6	GC DDLNVFECI CPCIWPELCPWIRCC
M-7	GC ELSSNRVCD CPCIWPELCPWIRCC
M-8	GC HMEGALTCE CPCIWPELCPWIRCC
M-9	GC GNSEDDSQR CPCIWPELCPWIRCC
M-10	GC SEFNHSSRS CPCIWPELCPWIRCC
M-11	GC RKSPENECA CPCIWPELCPWIRCC
M-12	GC PSDIENEES CPCIWPELCPWIRCC
M-13	GC EHVTFLDCD CPCIWPELCPWIRCC
M-14	GC NQETSEINQ CPCIWPELCPWIRCC
M-15	GC VEEVTRESD CPCIWPELCPWIRCC
M-16	GC ELNTDNHCN CPCIWPELCPWIRCC
M-17	GC EASPDREID CPCIWPELCPWIRCC
M-18	GC KYATIRECE CPCIWPELCPWIRCC
M-19	GC NEPHNANCA CPCIWPELCPWIRCC
M-20	GC APTSLSSCE CPCIWPELCPWIRCC
M-21	GC GQSTHNSCE CPCIWPELCPWIRCC
M-22	GC LEDVNYRTN CPCIWPELCPWIRCC
M-23	GC DLEIMICQN CPCIWPELCPWIRCC
M-24	GC SVTNHEEED CPCIWPELCPWIRCC

Figure S13. a) Sequences obtained from screening of **8**-X_5 against MDM2 after three rounds of panning. b) Sequences obtained from screening of **8**-X_9 against MDM2 after three rounds of panning.

Clones			Sequences						
M-1	GC	EKATESESP	CPC	IF	P	EL	CP	WIV	CC
M-2	GC	EKATESESP	CPC	IF	P	EL	CP	WIV	CC
M-3	GC	EKATESESP	CPC	IF	P	EL	CP	WIV	CC
M-4	GC	EKATESESP	CPC	IF	P	EL	CP	WIV	CC
M-5	GC	EKATESESP	CPC	IF	P	EL	CP	WIV	CC
M-6	GC	EKATESESP	CPC	IF	P	EL	CP	WIV	CC
M-7	GC	EKATESESP	CPC	IF	P	EL	CP	WIV	CC
M-8	GC	AEGVEKIIK	CPC	IS	P	VM	CP	WLP	CC
M-9	GC	AEGVEKIIK	CPC	IS	P	VM	CP	WLP	CC
M-10	GC	AEGVEKIIK	CPC	IS	P	VM	CP	WLP	CC
M-11	GC	EVEVRAVCL	CPC	FY	P	SF	CP	WIK	CC
M-12	GC	EVEVRAVCL	CPC	FY	P	SF	CP	WIK	CC
M-13	GC	SAVEHVHAS	CPC	IL	P	EL	CP	WLT	CC
M-14	GC	REDSDERNG	CPC	NP	P	FL	CP	WIN	CC
M-15	GC	RSDEQMMDE	CPC	PV	P	YL	CP	WLP	CC
M-16	GC	STLQSRKME	CPC	PV	P	IM	CP	WLR	CC
M-17	GC	ASCADAYIE	CPC	VD	P	HF	CP	WMS	CC
M-18	GC	TIREVDREG	CPC	PL	P	QL	CP	WIQ	CC
M-19	GC	ISPENRHAE	CPC	ID	P	LL	CP	WLV	CC
M-20	GC	REQAVWVVG	CPC	LF	P	EL	CP	WIV	CC
M-21	GC	AEGVENIIK	CPC	IS	P	VM	CP	WLP	CC
M-22	GC	TKCNKVTTG	CPC	YY	P	IM	CP	WLS	CC
M-23	GC	HQEGTIVES	CPC	EE	P	LL	CP	WIF	CC
M-24	GC	CGEASNSSE	CPC	SP	P	SL	CP	WLP	CC

Figure S14. Sequences obtained from screening of **8**-X_{16} against MDM2 after three rounds of panning.

Table S6. Chemical shifts of oxidized **15** (BMRB 50900)

Residue	N	H_N	Cα	Hα	Cβ	Hβ	Other
G1	112.7	7.927	42.85	3.838			
C2		8.133		4.584	38.33	3.178, 2.292	
E3	120.6	8.082			25.96	2.029, 1.906	30.1 (C γ), 2.375 (H γ 2)
K4	120.6	7.929		4.21	22.04	1.325	26.39 (C δ), 1.589 (H δ 2), 24.62 (C γ), 2.892 (H ε 2), 1.548 (H γ 2), 0.914 (H γ 3), 7.344 (H ζ 1)
A5	123.7	8.074		4.255	16.69	1.352	
T6	109.6			4.279	57.28	3.555	18.96 (C γ 2), 1.11 (H ζ 21), 7.641 (H ν)
E7	119.2	8.07		4.183	25.59	2.085, 1.95	2.389 (H γ 2)
S8	112.6	7.787	55.76	4.316	61.3	3.79, 3.724	
E9	119.8	7.708	52.94	4.299	26.43	2.027, 1.88	2.357 (H γ 2)
S10	115.8	7.971	52.85	4.675	61.34	3.703	
P11				4.306	29.29	2.168, 1.867	47.85 (C δ), 3.689 (H δ 2), 3.621 (H δ 3)
C12		7.99	49.79	4.763	39.42	3.027, 2.901	
P13				4.675			48.15 (C δ), 24.13 (C γ), 3.877 (H δ 2), 3.71 (H δ 3), 2.012 (H γ 2), 1.805 (H γ 3)
C14	121	7.08	52.59	4.598	41.14	2.979	
I15	122.8	7.921	60.92	3.57	35.24	1.431	10.47 (C δ 1), 25.17 (C γ 1), 14.27 (C γ 2), 0.7076 (H δ 11), 1.191 (H γ 11),
							0.9895 (H γ 12), 0.3744 (H γ 21)
F16		7.101	51.09	5.082	36.02	2.963	7.525 (H δ 2), 7.452 (H ε 2), 7.123 (H ζ)
P17			63.34	3.914	30.06	2.51, 1.931	48.2 (C δ), 3.815 (H δ 2), 3.792 (H δ 3)
E18	113.2	8.126	54.98	3.972	24.71	1.998, 1.934	30.47 (C γ), 2.426 (H γ 2)
L19	115.6	7.83	51.67	4.32	38.67	2.002, 1.767	22.85 (C δ 1), 19.62 (C δ 2), 24.64 (C γ), 0.9517 (H δ 11), 0.8025 (H δ 21), 1.597 (H γ)
C20	115.4	7.445	49.32	4.447	40.97	3.01	
P21			61.76	4.119	28.85	2.015, 1.519	47.58 (C δ), 24.3 (C γ), 3.265 (H δ 2), 1.763 (H γ 2), 1.407 (H γ 3)
W22	115	6.761	54.31	4.591	25.59	3.325, 3.173	7.054 (H δ 1), 10.04 (H ε 1), 7.255 (H ε 3), 7.191 (H η 2), 7.191 (H ζ 2), 7.243 (H ζ 3), 129.5 (N ε 1)
I23	127	8.281	62.74	3.766	18.85	1.107	10.38 (C δ 1), 30.42 (C γ 1), 18.26 (C γ 2), 0.8415 (H δ 11), 1.757 (H γ 12),
							1.615 (H γ 13), 0.944 (H γ 21)
V24	117.2	7.076		4.19	35.24	1.737	15.33 (C γ 1), 17.16 (C γ 2), 0.9086 (H δ 11), 1.116 (H γ 21)
C25		8.047	52.8	4.537	40.23	3.364	
C26		7.912	53.38	4.566	36.22	3.179, 2.919	

Table S7. Chemical shifts of oxidized **16** (BMRB 50871)

Redidue	N	H_N	Cα	Hα	Cβ	Hβ	Other
G1		7.833	42.85	3.791			
C2	116.7	8.007	52.87	4.478	38.03	3.051, 2.884	
M3	120.3	8.142	53.77	4.324	30.39	1.993, 1.956	29.59 (C γ), 2.526 (H γ 2), 2.435 (H γ 3)
I4	118.2	7.549	58.67	4.082	36.2	1.792	10.46 (C δ), 0.793 (H δ), 24.9 (C γ 1), 1.393 (H γ 12), 1.094 (H γ 13), 14.84 (C γ 2), 0.807 (H γ 22)
L5	122.6	7.724	52.23	4.335	39.74	1.568, 1.484	22.43 (C δ 1) , 0.8367 (H δ 12), 20.86 (C δ 2), 0.7793 (H δ 22), 24.42 (C γ)
L6	120.1	7.853		4.205	39.61	1.632, 1.516	22.49 (C δ 1), 20.77 (C δ 2), 0.8307 (H δ 12), 0.793 (H δ 22)
D7		8.005	50.83	4.581	35.15	2.894, 2.798	
T8	111.3	7.665		4.226	66.96	4.18	18.91 (C γ 2) , 1.113 (H γ 22)
D9	118.9	8.002	50.66	4.623	35.48	2.811, 2.763	
I10	117.6	7.516	59.05	3.989	36.14	1.624	10.57 (C δ 1), 0.6572 (H δ 12), 24.28 (C γ 1), 1.101 (H γ 12), 0.9157 (H γ 13), 14.63 (C γ 2), 0.5924 (H γ 22)
W11	121.1	7.655	54.06	4.62	27.27	3.253, 3.018	7.121 (H δ 1), 9.891 (H ε 1), 7.535 (H ε 3), 7.097 (H η 2), 7.356 (H ζ 2), 7.02 (H ζ 3),
C12	112.3	7.843	50.23	4.79	39.22	3.02, 2.952	
P13			61.04	4.26	29.06	1.996, 1.91	24.2 (C γ), 1.793 (H γ 3), 3.581 (H δ 2), 3.356 (H δ 3)
C14		7.374	52.04	4.662	37.68	3.187, 2.877	
S15	117.7	8.208	57.16	4.094	60.64	3.688, 3.664	
H16	116.8	7.335	49.68	4.913	26.37	2.842	7.271 (H δ 2), 8.498 (H ε 1)
P17			62.2	4.118	29.21	2.125, 1.758	48.1 (C δ), 3.714 (H δ 2), 3.606 (H δ 3), 24.79 (C γ), 1.956 (H γ 2), 1.911 (H γ 3)
Y18	118	8.07		4.122	33.85	3.102, 3.078	7.003 (H δ 2)
A19	121.3	7.696	49.95	4.28	17	1.313	
C20	112.3	7.857	50.25	4.934	39.84	3.213, 3.04	
P21			60.88	4.351	28.89	2.186	47.7 (C δ), 3.559 (H δ 2), 3.52 (H δ 3), 24.75 (C γ), 1.926 (H γ 2)
E22	117	7.965		4.218	25.83	2.071, 1.962	29.94 (C γ), 2.358 (H γ 2)
N23		7.925	50.7	4.598	36.42	2.769, 2.718	7.333 (H δ 21), 6.543 (H δ 22), 110.9 (N δ 2)
I24	121.7	7.928	59.62	4.151	36	1.841	10.87 (C δ), 0.8257 (H δ), 24.93 (C γ 1), 1.428 (H γ 12), 1.164 (H γ 13), 15.14 (C γ 2), 0.8938 (H γ 22)
C25	118.4	8.425	52.32	4.611	39.8	3.204, 2.857	
C26	118	8.039	53.06	4.465	39.72	3.216, 3.023	

a) **b)**

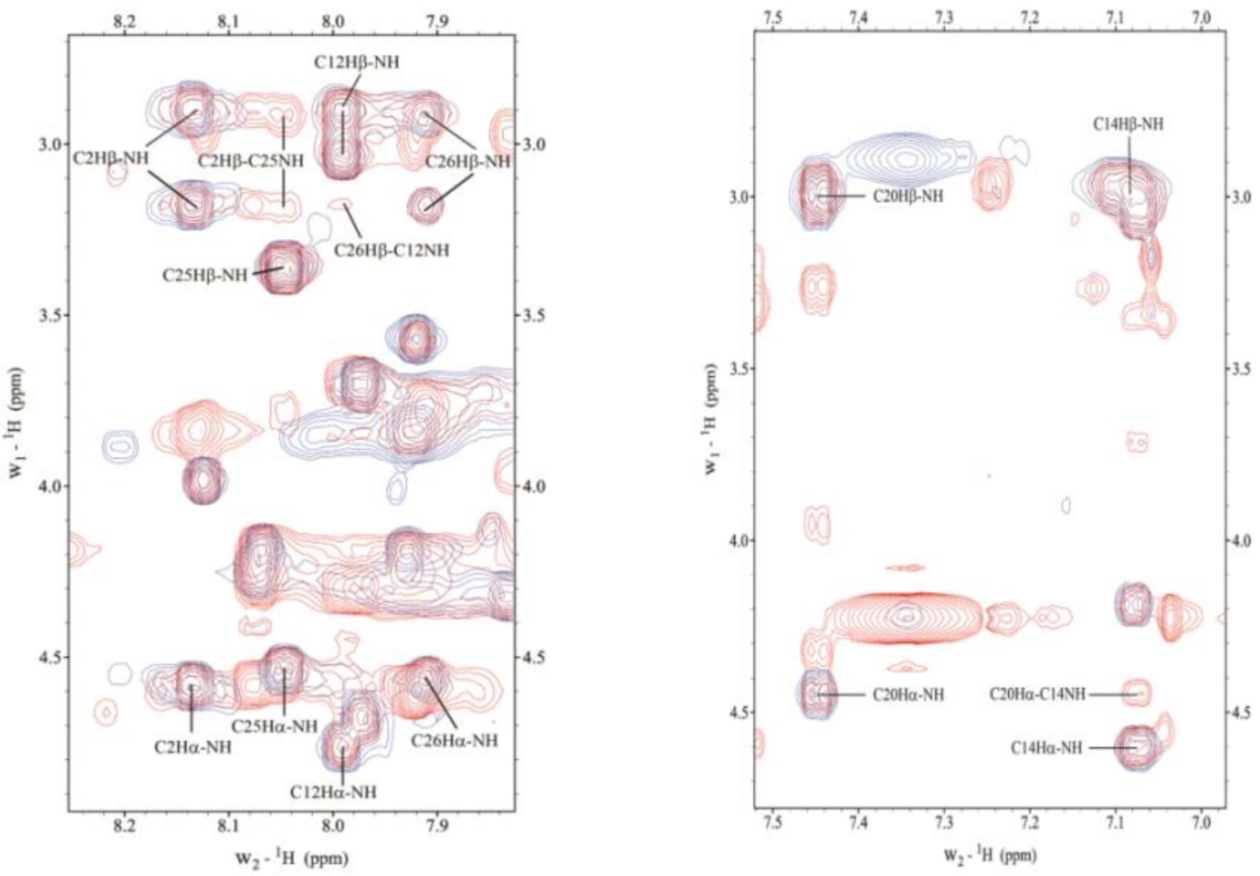

Figure S15. NMR characterization of oxidized **15**. a) and b) 2D 1H-1H NOESY (red) and TOCSY (blue) spectra of oxidized **15** in ACN/H_2O (50%/50%, v/v). Only local regions were plotted to show cross peaks of cysteine residues (disulfide connectivity: Cys 2-Cys 25, Cys 12-Cys 16, Cys 14-Cys 20) and cysteine-related assignments were labeled (data deposited in BMRB, accession number 50900).

Clones	Sequences								
B-1	GC	MILLDTDIW	CPC	SH	P	YA	CP	ENI	CC
B-2	GC	MILLDTDIW	CPC	SH	P	YA	CP	ENI	CC
B-3	GC	MILLDTDIW	CPC	SH	P	YA	CP	ENI	CC
B-4	GC	MILLDTDIW	CPC	SH	P	YA	CP	ENI	CC
B-5	GC	MILLDTDIW	CPC	SH	P	YA	CP	ENI	CC
B-6	GC	MILLDTDIW	CPC	SH	P	YA	CP	ENI	CC
B-7	GC	MILLDTDIW	CPC	SH	P	YA	CP	ENI	CC
B-8	GC	MILLDTDIW	CPC	SH	P	YA	CP	ENI	CC
B-9	GC	MILLDTDIW	CPC	SH	P	YA	CP	ENI	CC
B-10	GC	MILLDTDIW	CPC	SH	P	YA	CP	YNI	CC
B-11	GC	WTAMTLSAR	CPC	ER	P	HG	CP	RGC	CC
B-12	GC	FRKSPRLEV	CPC	AE	P	RA	CP	RGC	CC
B-13	GC	SQTNCYRLS	CPC	KT	P	TQ	CP	VVI	CC
B-14	GC	SIEFLSFLG	CPC	LS	P	AF	CP	FLY	CC
B-15	GC	PEPLTWLTE	CPC	VE	P	IP	CP	DWM	CC
B-16	GC	LIMILFGEW	CPC	VM	P	LE	CP	PTR	CC
B-17	GC	LEFGCGVSS	CPC	EN	P	TH	CP	WAH	CC
B-18	GC	ARESGFMIL	CPC	QS	P	RA	CP	ESS	CC
B-19	GC	FLFFSRLPE	CPC	SS	P	CF	CP	PDR	CC
B-20	GC	YAVVCLHHE	CPC	LY	P	LL	CP	CLW	CC

Figure S16. Sequences obtained from screening of **8**-X_{16} against Bcl-2 after four rounds of panning.

2.6 Binding affinity of the oxidized peptides to target proteins

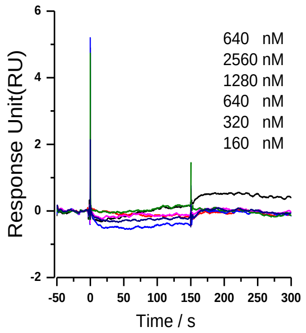

Figure S17. Sensorgrams recorded by surface plasmon resonance (SPR) showing the binding of **16**-Ala with Bcl-2.

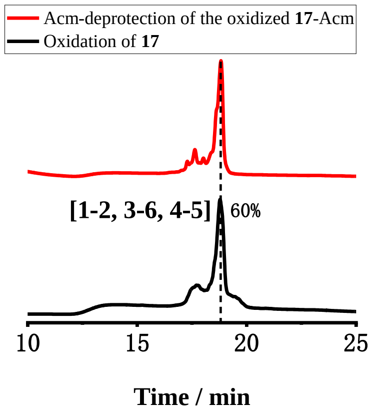

Figure S18. Chromatogram of products formed after the oxidation of **17** (black line) and the Acm-deprotection of the oxidized **17-Acm** (red line).

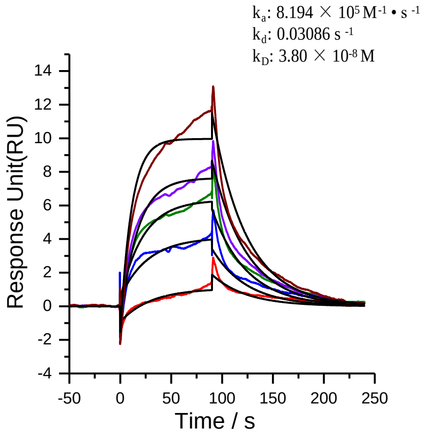

Figure S19. Sensorgrams recorded by surface plasmon resonance (SPR) showing the binding of the oxidized **17** with streptavidin.

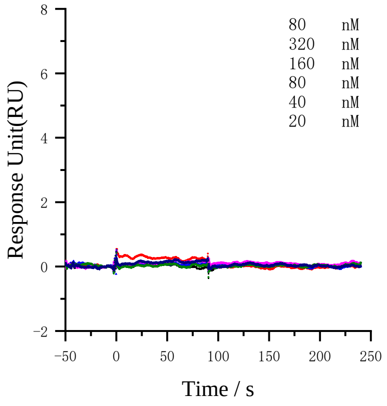

Figure S20. Sensorgrams recorded by surface plasmon resonance (SPR) showing the binding of the oxidized **11** with streptavidin.

a)

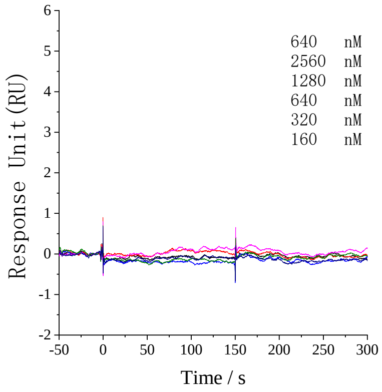

b)

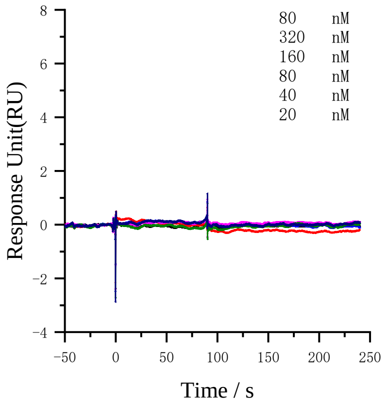

Figure S21. a) Sensorgrams recorded by surface plasmon resonance (SPR) showing the binding of oxidized **15** with Bcl-2. b) Sensorgrams recorded by surface plasmon resonance (SPR) showing the binding of the oxidized **15** with streptavidin.

a) b)

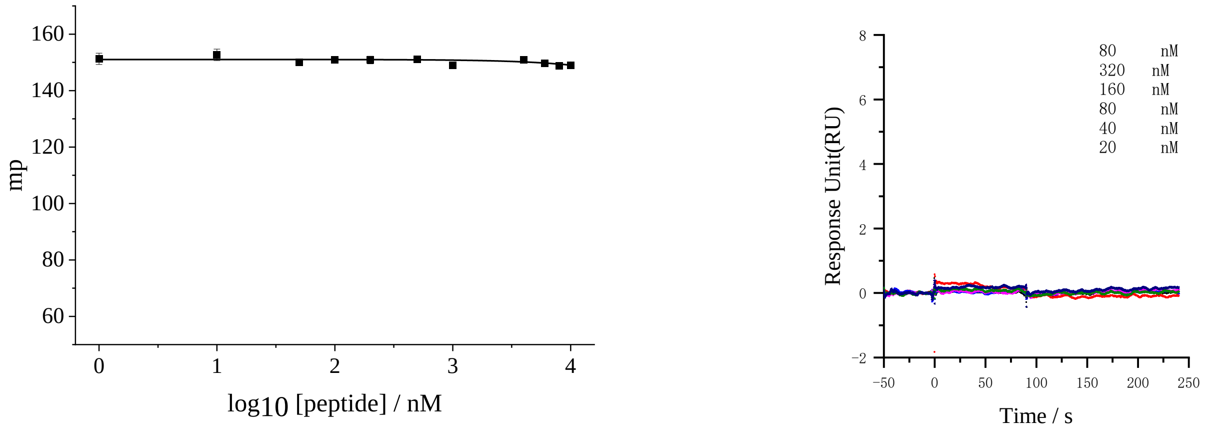

Figure S22. a) A fluorescence polarization competition assay showing the binding of oxidized **16** to MDM2. b) Sensorgrams recorded by surface plasmon resonance (SPR) showing the binding of the oxidized **16** with streptavidin.

a) b)

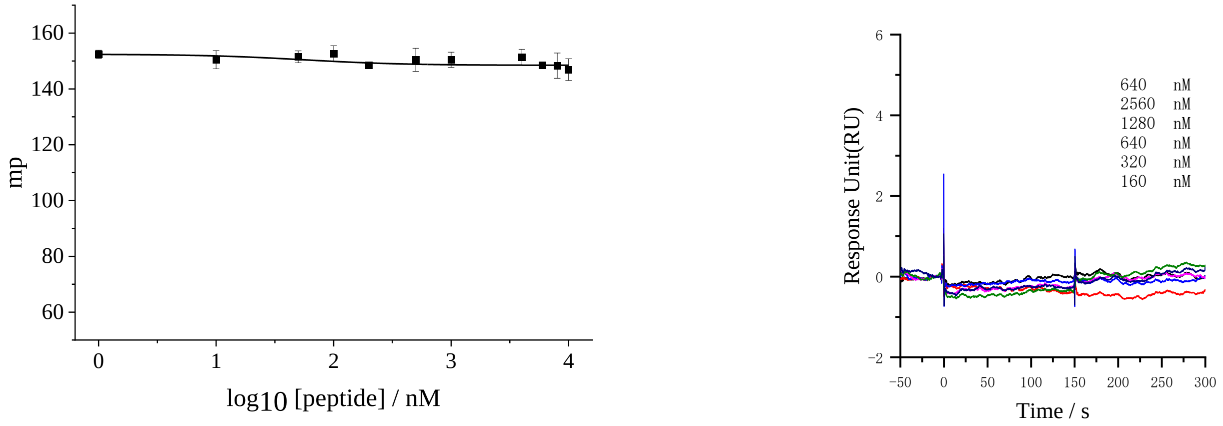

Figure S23. a) A fluorescence polarization competition assay showing the binding of oxidized **17** to MDM2. b) Sensorgrams recorded by surface plasmon resonance (SPR) showing the binding of the oxidized **17** with Bcl-2.

2.7 Proteolytic stability of peptides

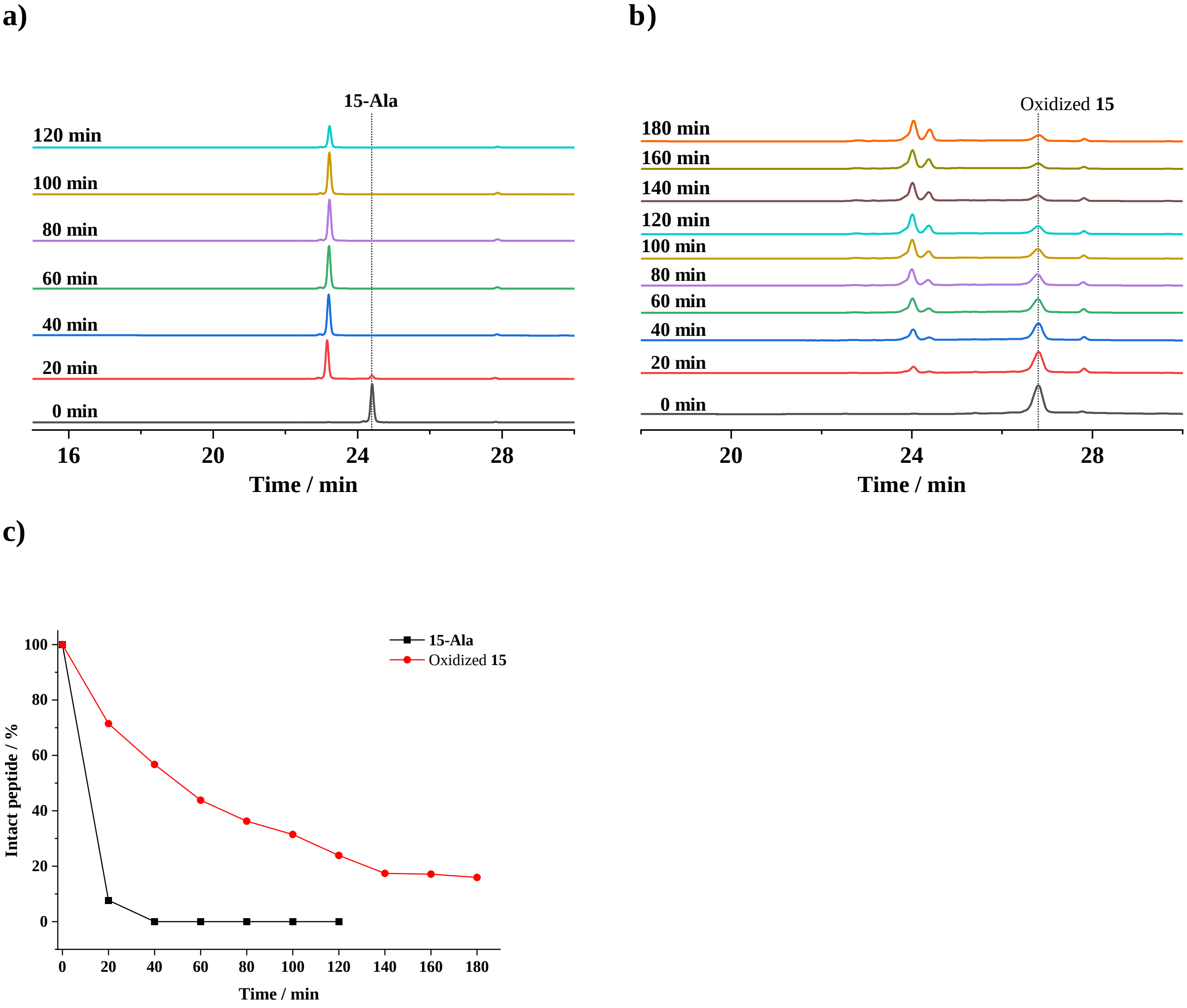

Figure S24. a) Chromatograms of **15**–Ala digested by chymotrypsin. b) Chromatograms of oxidized **15** digested by chymotrypsin. c) Kinetics of peptide degradation by chymotrypsin in phosphate buffer at pH 7.4. Concentrations of peptides and chymotrypsin were 100 μM and 300 ng·mL^{-1}, respectively.

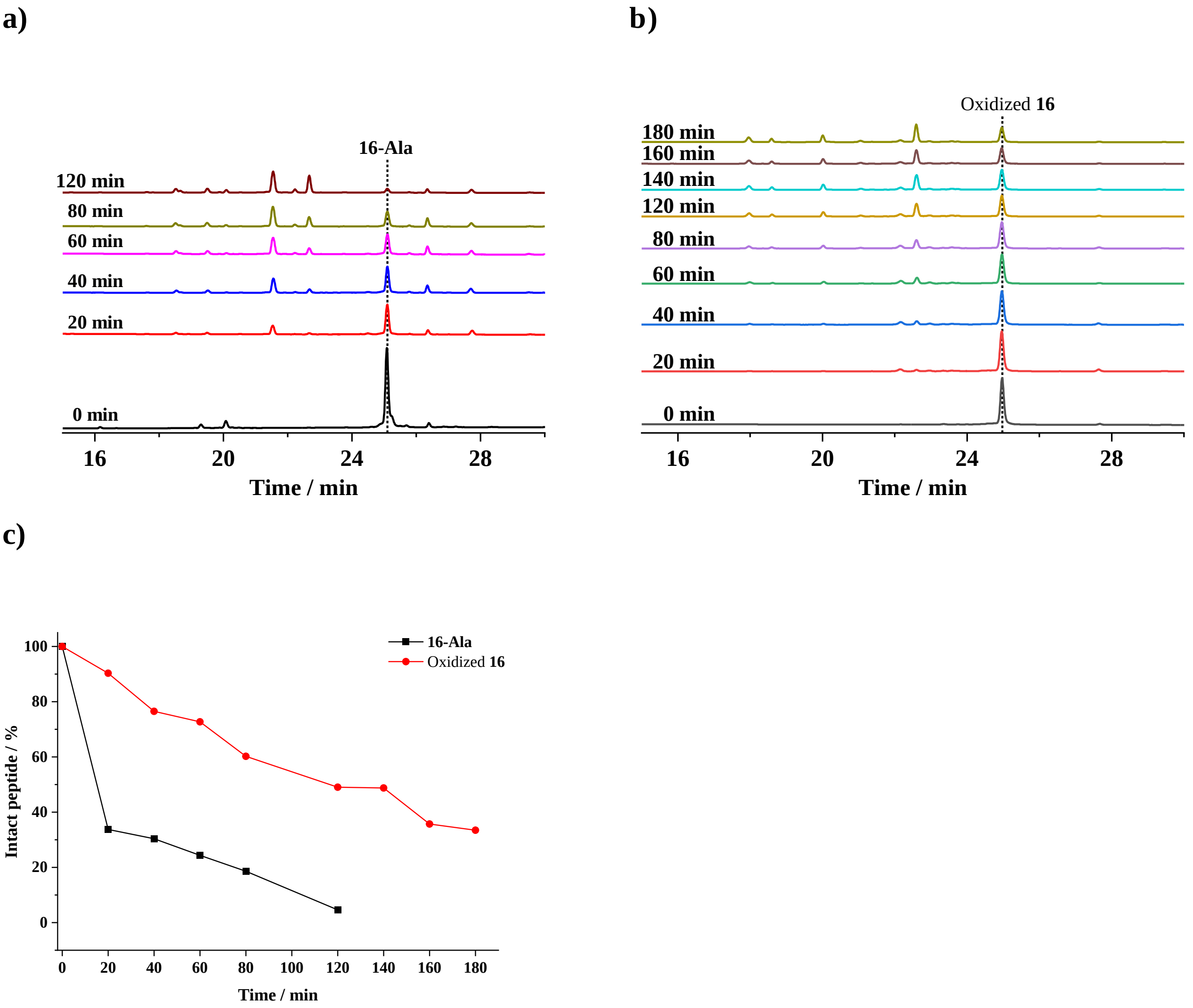

Figure S25. a) Chromatograms of **16**–Ala digested by chymotrypsin. b) Chromatograms of oxidized **16** digested by chymotrypsin. c) Kinetics of peptide degradation by chymotrypsin in phosphate buffer at pH 7.4. Concentrations of peptides and chymotrypsin were 100 µM and 5 µg·mL^{-1}, respectively.

2.8 CD spectra of the oxidized **15** and oxidized **16**

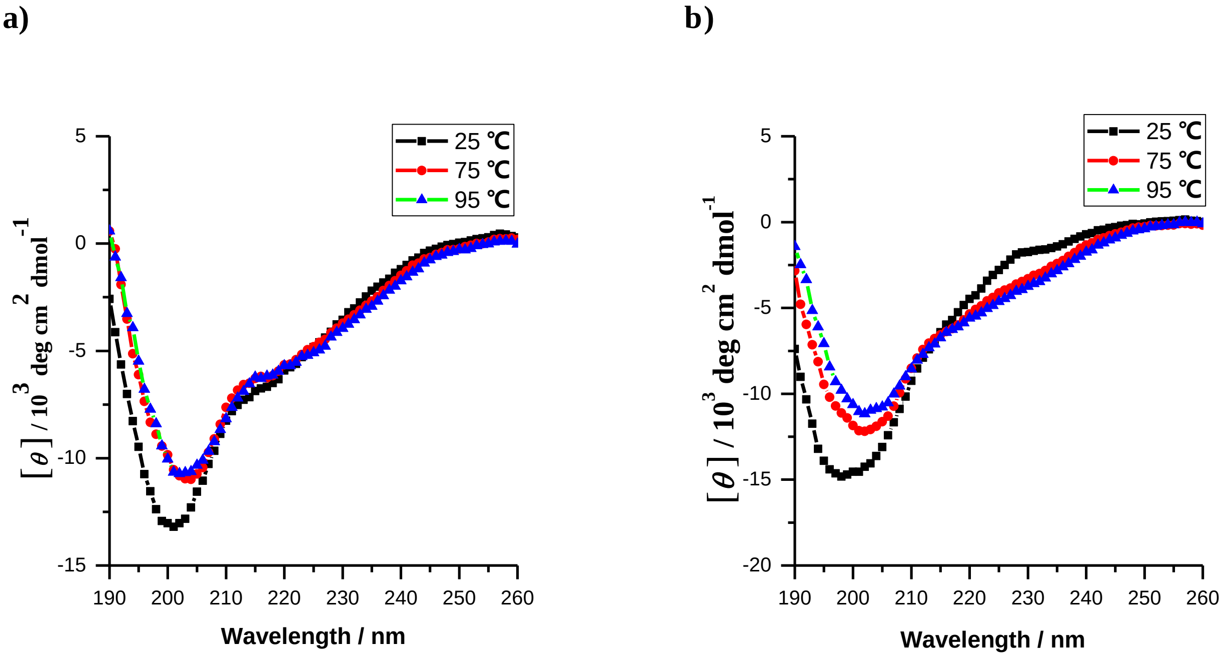

Figure S26. a) CD spectra of the oxidized **15** (50 μM) in water. b) CD spectra of the oxidized **16** (50 μM) in water.

2.9 Mass spectra of peptides

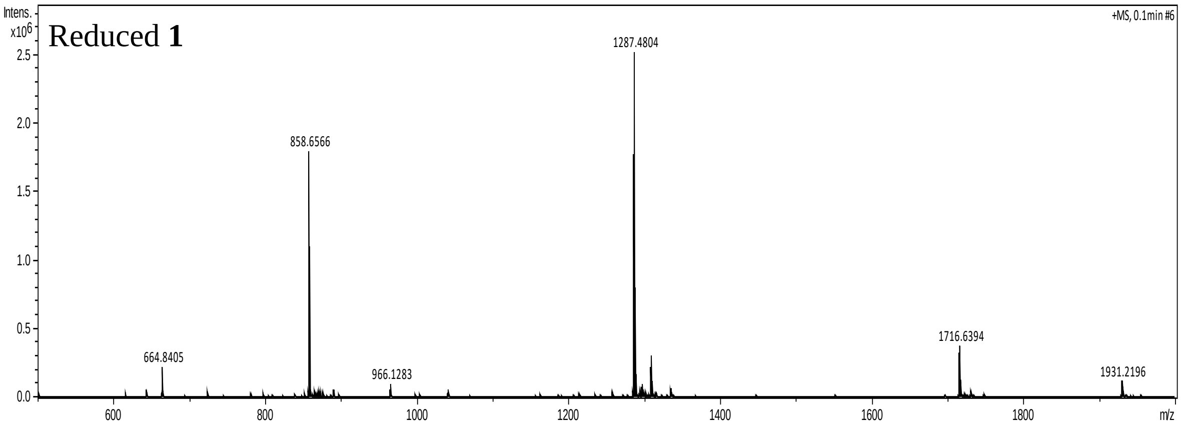

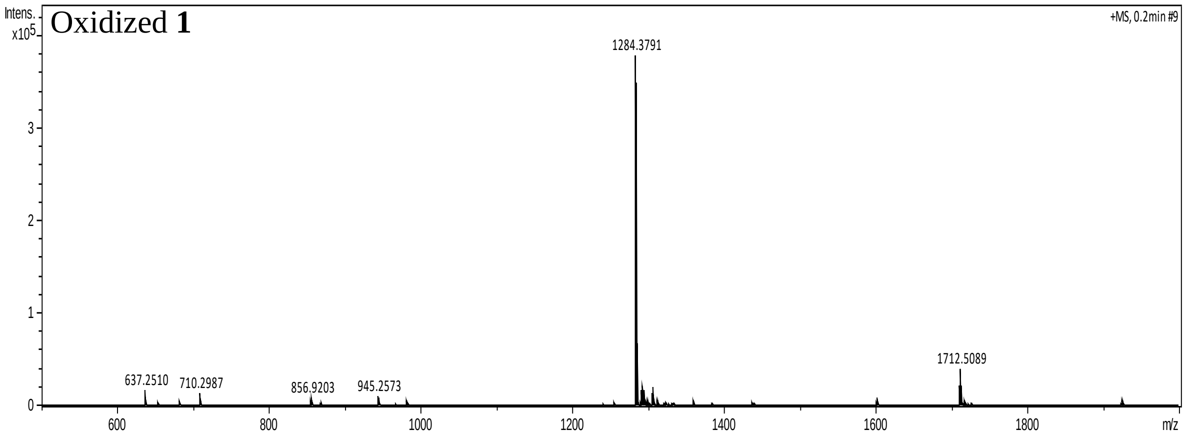

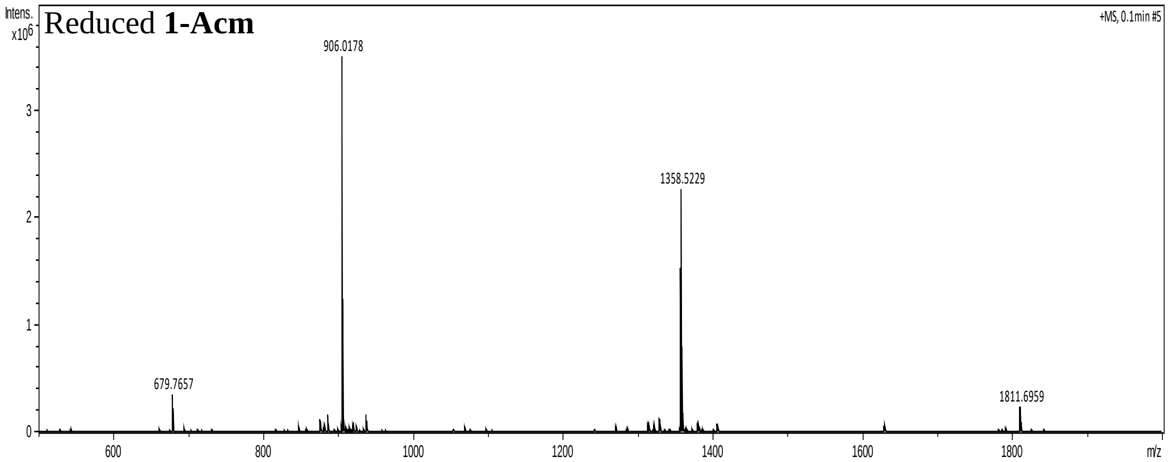

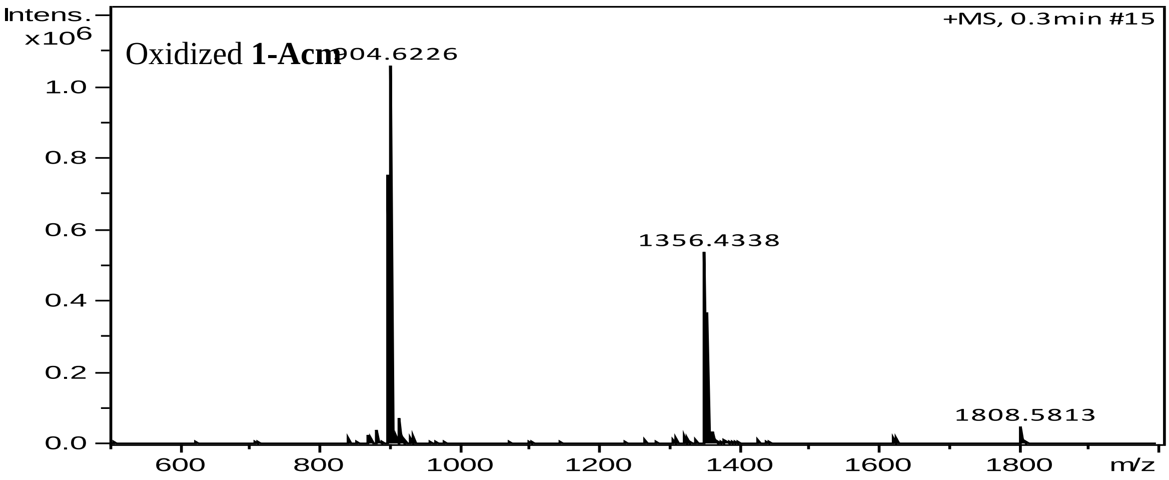
Intens.
x10^6
Oxidized 1-Acm
904.6226
1356.4338
1808.5813
+MS, 0.3min #15
1.0
0.8
0.6
0.4
0.2
0.0
600
800
1000
1200
1400
1600
1800
m/z

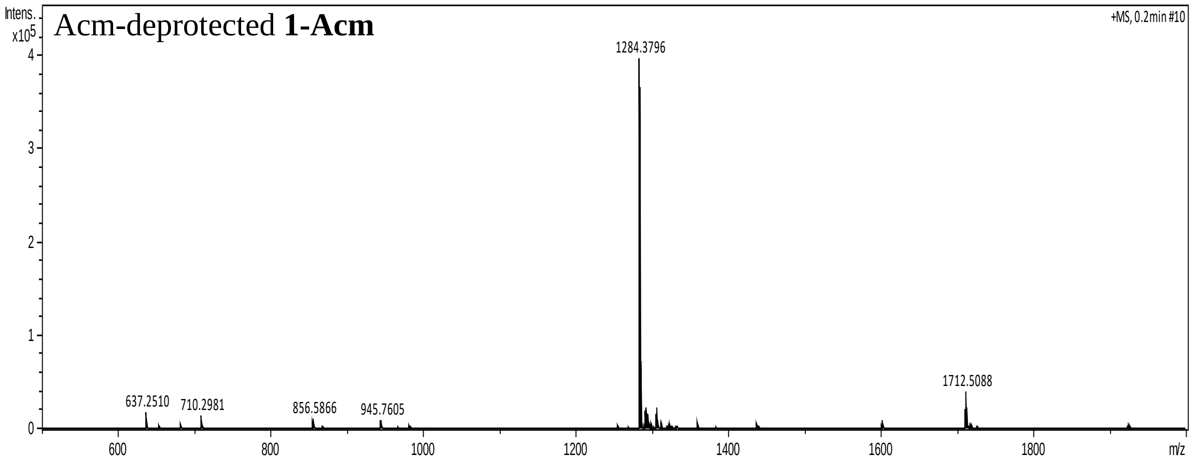
Intens.
x10^5
Acm-deprotected 1-Acm
+MS, 0.2min #10
1284.3796
1712.5088
637.2510
710.2981
856.5866
945.7605
4
3
2
1
0
600
800
1000
1200
1400
1600
1800
m/z

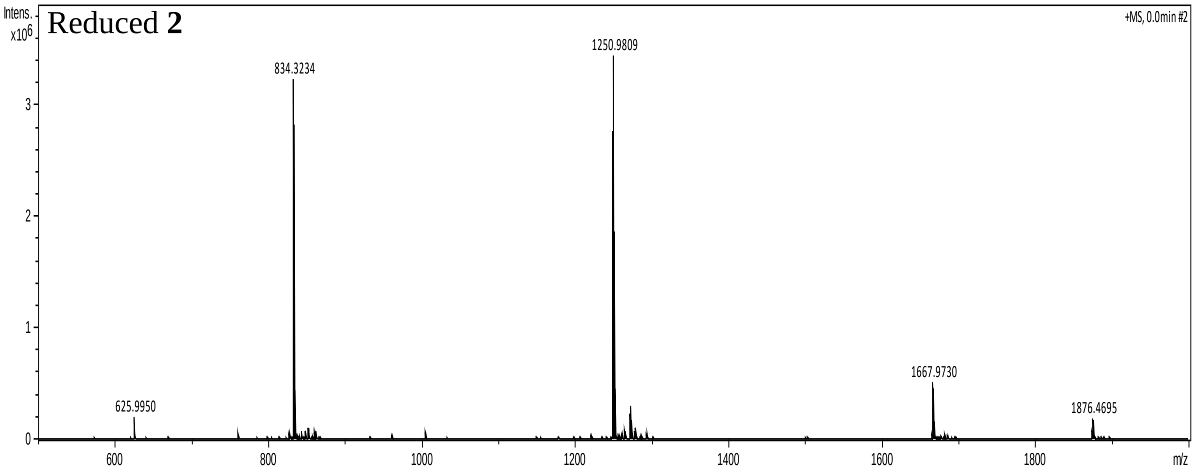
Intens.
x10^6
Reduced 2
+MS, 0.0min #2
1250.9809
834.3234
1667.9730
1876.4695
625.9950
3
2
1
0
600
800
1000
1200
1400
1600
1800
m/z

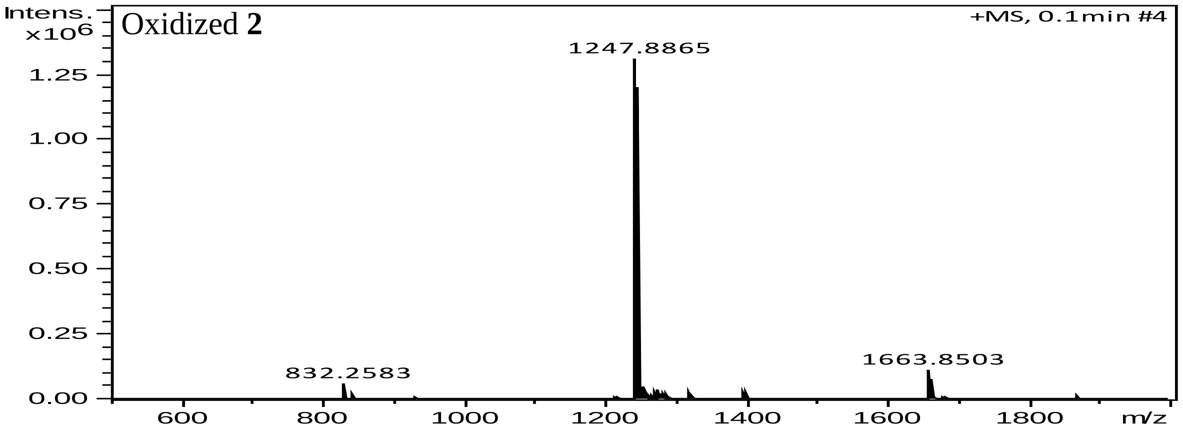
Oxidized 2
+MS, 0.1min #4
Intens. x10^6
1247.8865
832.2583
1663.8503
m/z

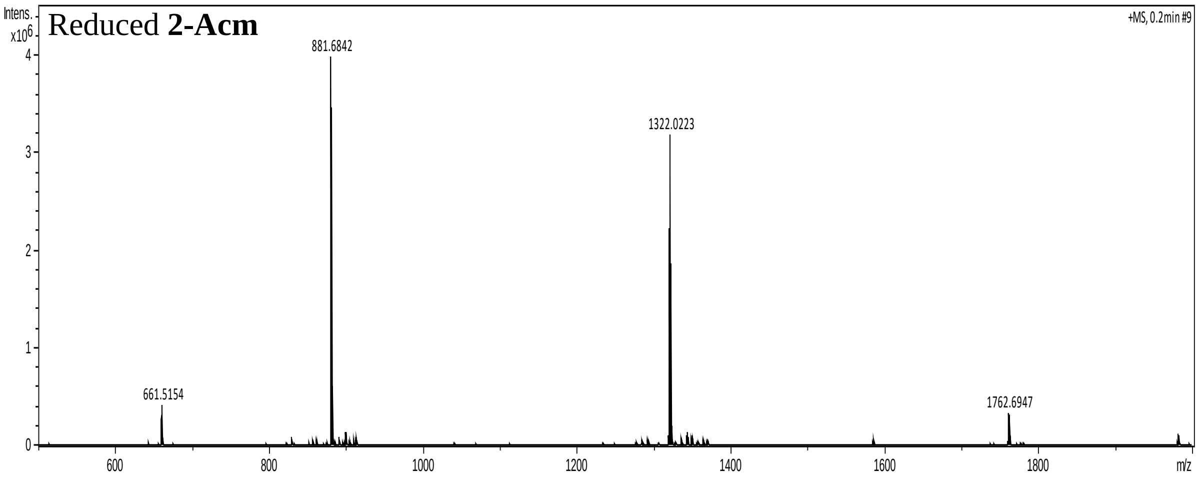
Reduced 2-Acm
+MS, 0.2min #9
Intens. x10^6
881.6842
1322.0223
661.5154
1762.6947
m/z

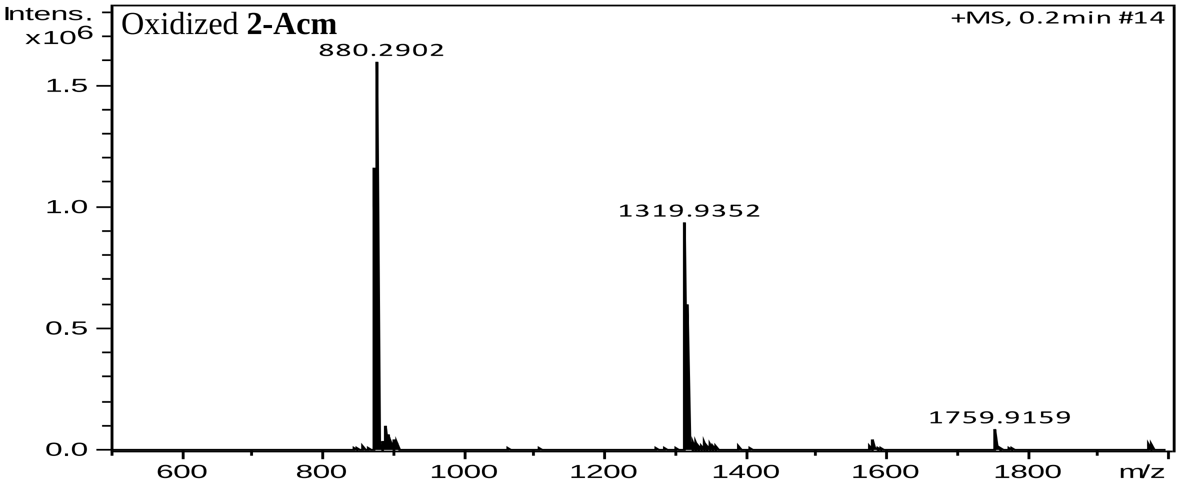
Oxidized 2-Acm
+MS, 0.2min #14
Intens. x10^6
880.2902
1319.9352
1759.9159
m/z

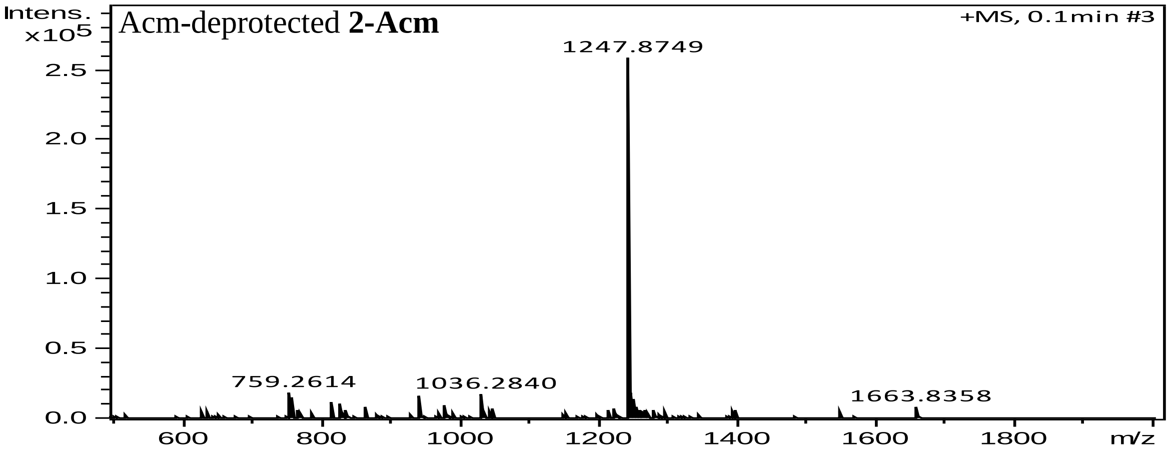
Intens.
x10^5
Acm-deprotected 2-Acm
+MS, 0.1min #3
1247.8749
759.2614
1036.2840
1663.8358
m/z

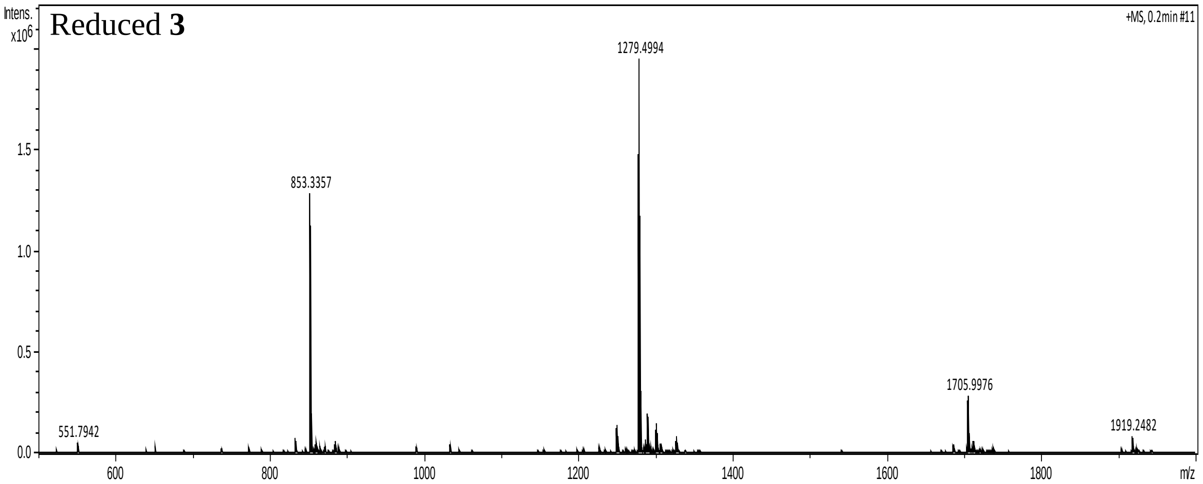
Intens.
x10^6
Reduced 3
+MS, 0.2min #11
1279.4994
853.3357
1705.9976
1919.2482
551.7942
m/z

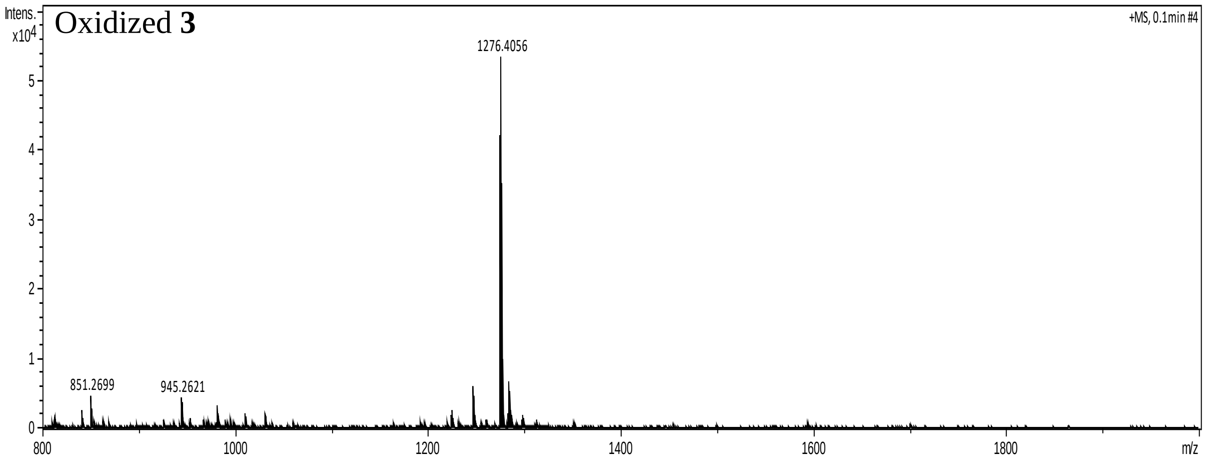
Intens.
x10^4
Oxidized 3
+MS, 0.1min #4
1276.4056
851.2699
945.2621
m/z

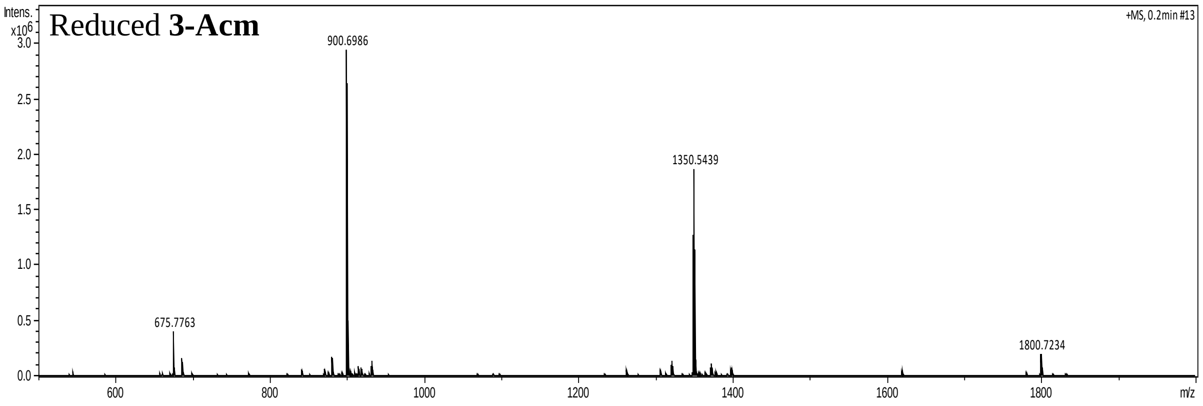
Reduced **3-Acm**
Intens.
x10^6
+MS, 0.2min #13
900.6986
1350.5439
675.7763
1800.7234
m/z

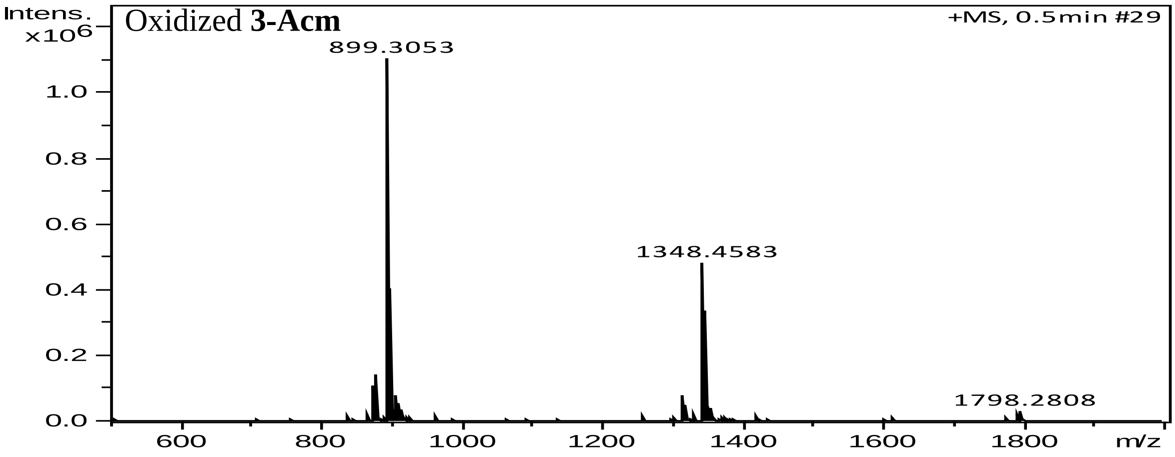
Oxidized **3-Acm**
Intens.
x10^6
+MS, 0.5min #29
899.3053
1348.4583
1798.2808
m/z

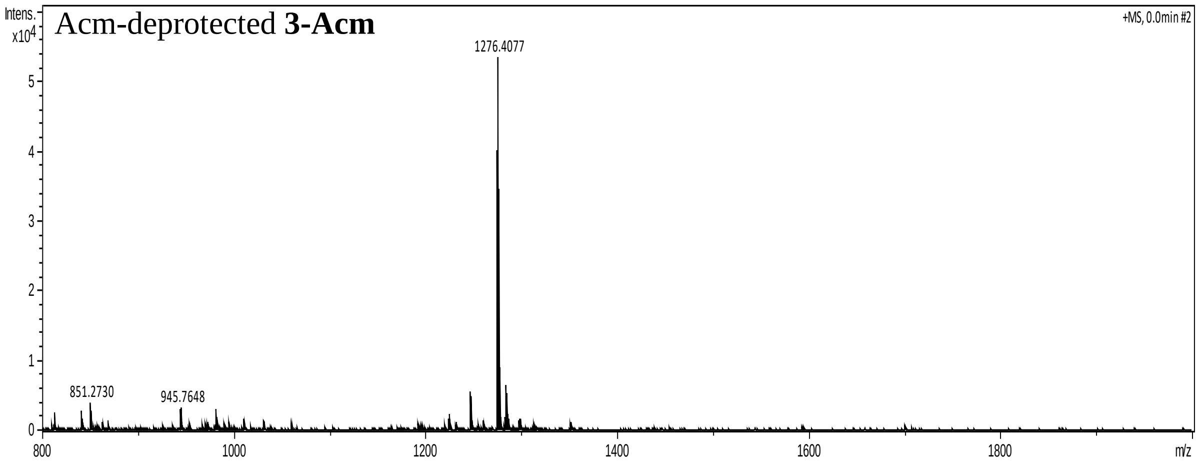
Acm-deprotected **3-Acm**
Intens.
x10^4
+MS, 0.0min #2
1276.4077
851.2730
945.7648
m/z

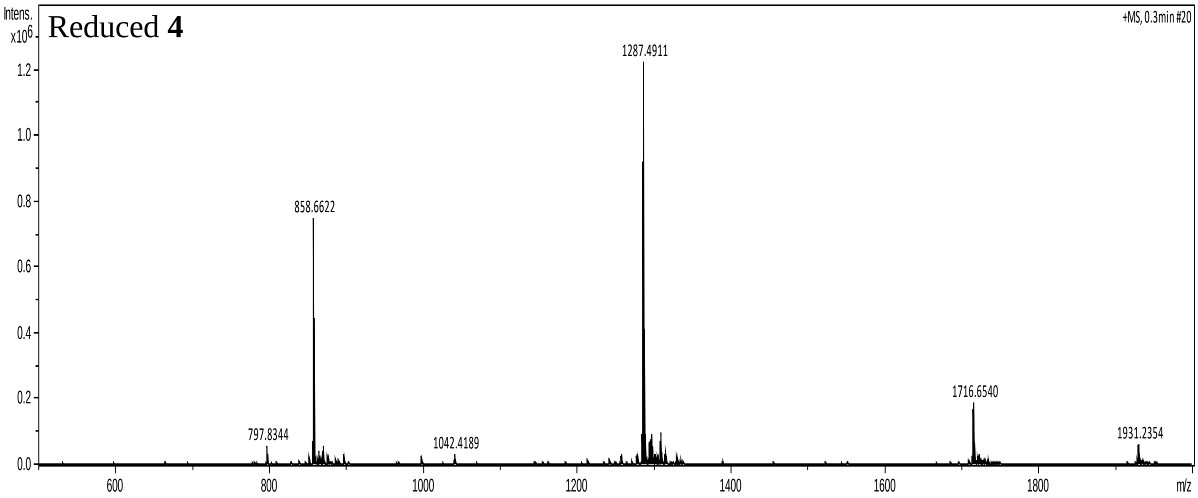
Reduced **4**
Intens.
x10^6
+MS, 0.3min #20
1287.4911
858.6622
797.8344
1042.4189
1716.6540
1931.2354
m/z

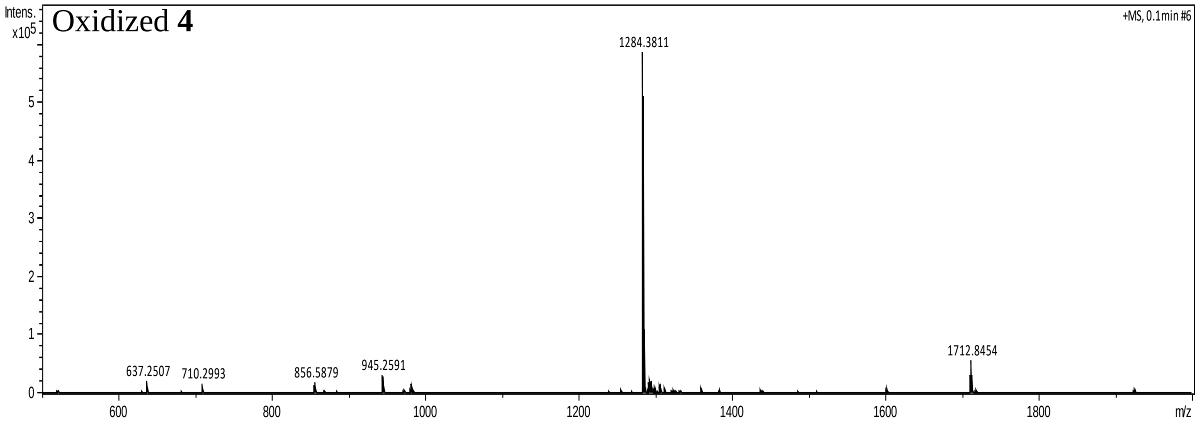
Oxidized **4**
Intens.
x10^5
+MS, 0.1min #6
1284.3811
637.2507
710.2993
856.5879
945.2591
1712.8454
m/z

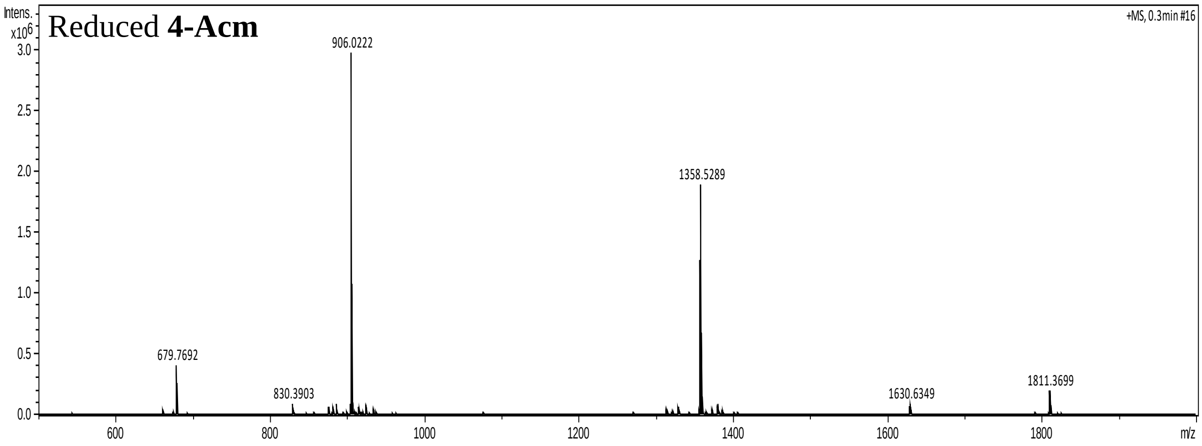
Reduced **4-Acm**
Intens.
x10^6
+MS, 0.3min #16
906.0222
1358.5289
679.7692
830.3903
1630.6349
1811.3699
m/z

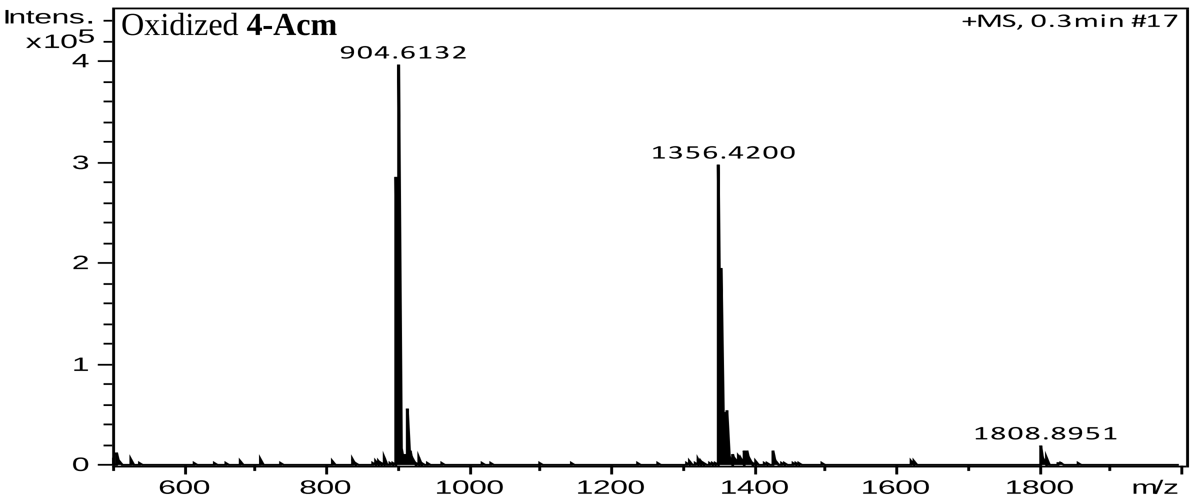
Oxidized 4-Acm
+MS, 0.3min #17
Intens. x10^5
904.6132
1356.4200
1808.8951
m/z

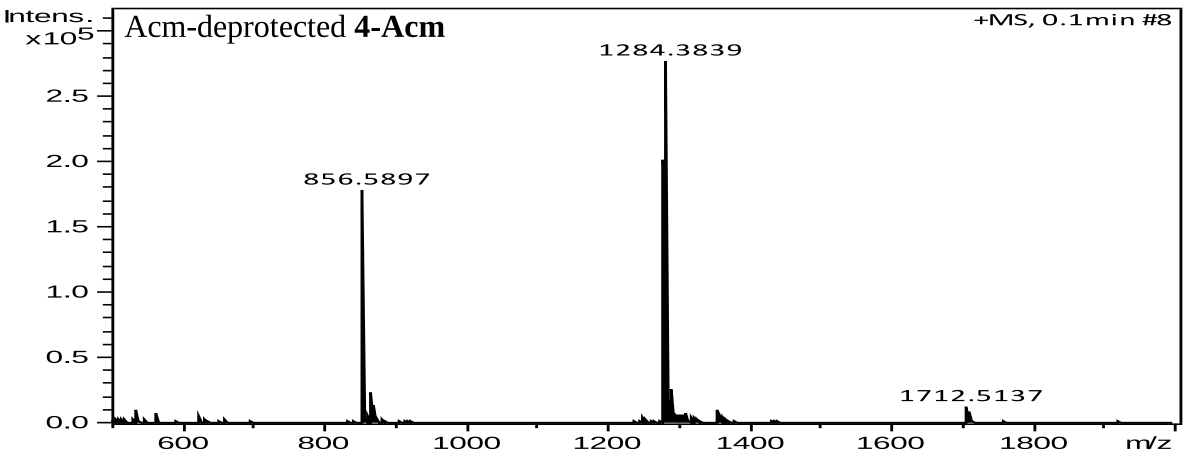
Acm-deprotected 4-Acm
+MS, 0.1min #8
Intens. x10^5
1284.3839
856.5897
1712.5137
m/z

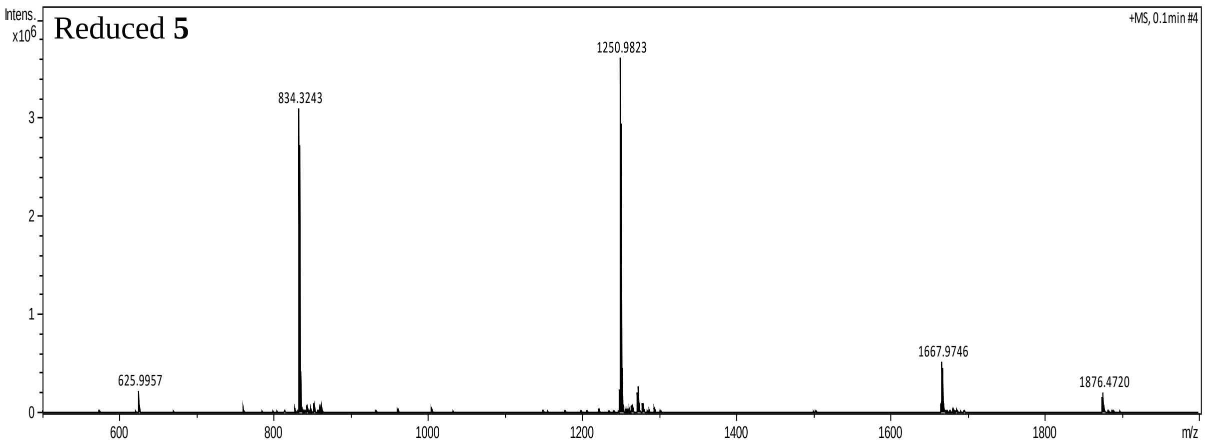
Reduced 5
+MS, 0.1min #4
Intens. x10^6
1250.9823
834.3243
625.9957
1667.9746
1876.4720
m/z

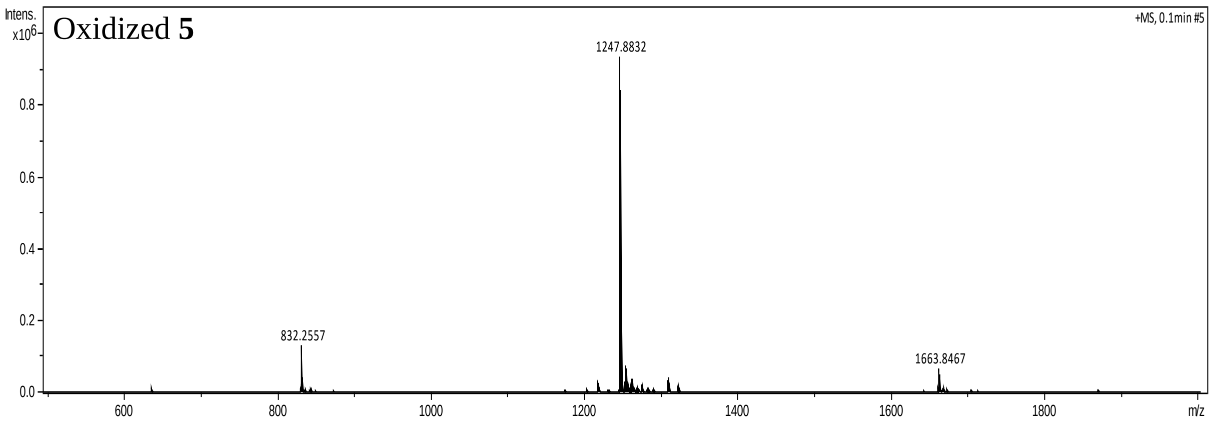
Intens.
x10^6
Oxidized 5
+MS, 0.1min #5
1247.8832
832.2557
1663.8467
0.0
0.2
0.4
0.6
0.8
600
800
1000
1200
1400
1600
1800
m/z

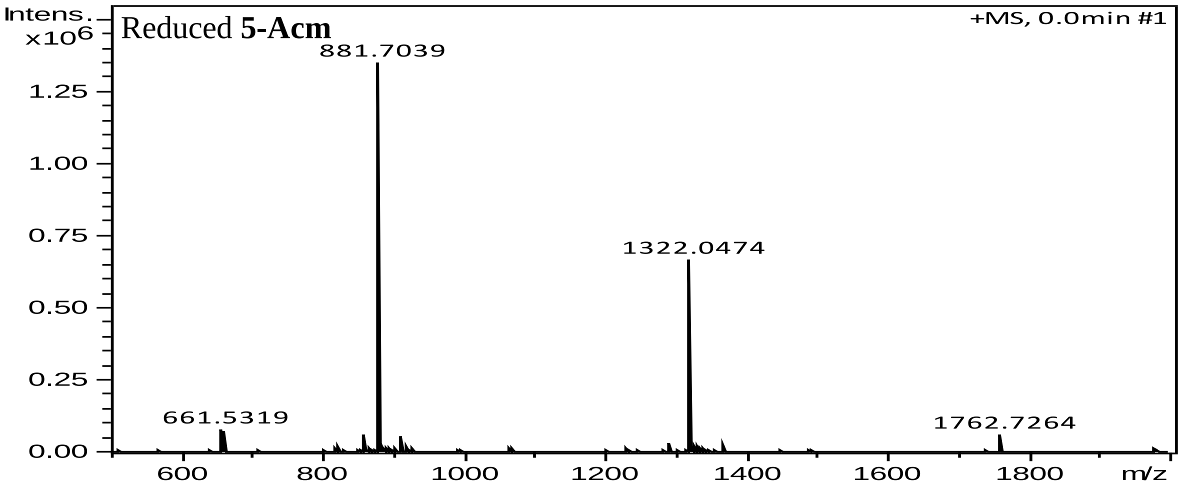
Intens.
x10^6
Reduced 5-Acm
+MS, 0.0min #1
881.7039
1322.0474
661.5319
1762.7264
0.00
0.25
0.50
0.75
1.00
1.25
600
800
1000
1200
1400
1600
1800
m/z

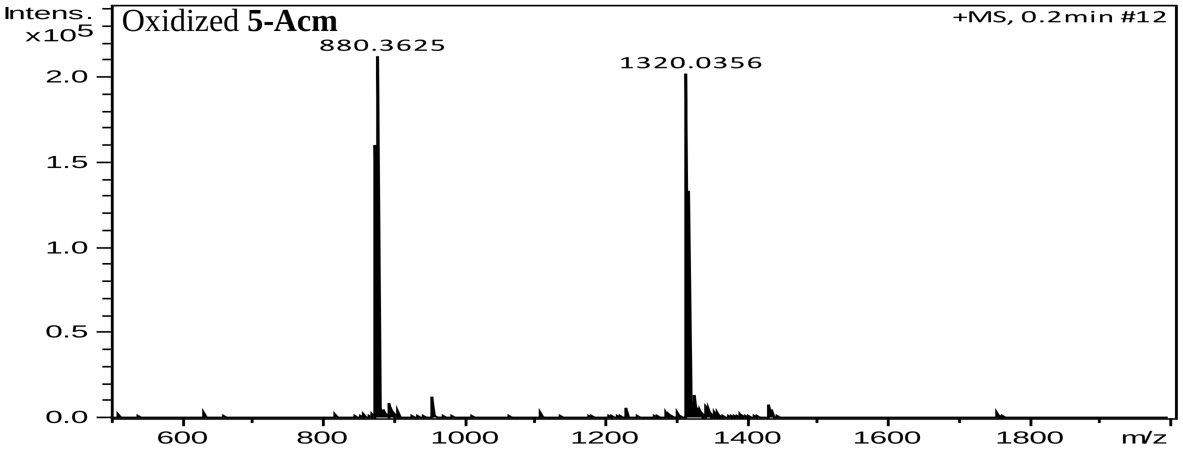
Intens.
x10^5
Oxidized 5-Acm
+MS, 0.2min #12
880.3625
1320.0356
0.0
0.5
1.0
1.5
2.0
600
800
1000
1200
1400
1600
1800
m/z

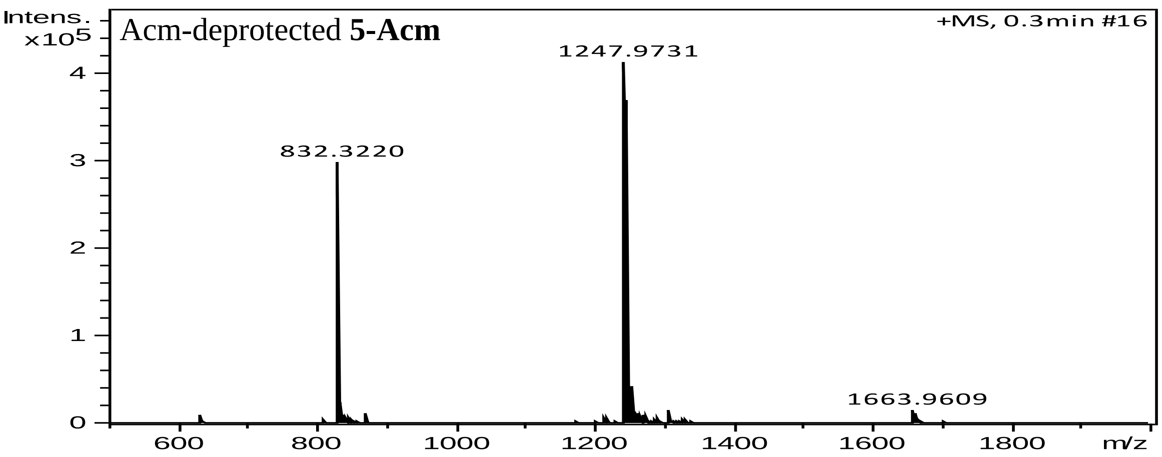
Intens.
x10^5
Acm-deprotected 5-Acm
+MS, 0.3min #16
1247.9731
832.3220
1663.9609
4
3
2
1
0
600
800
1000
1200
1400
1600
1800
m/z

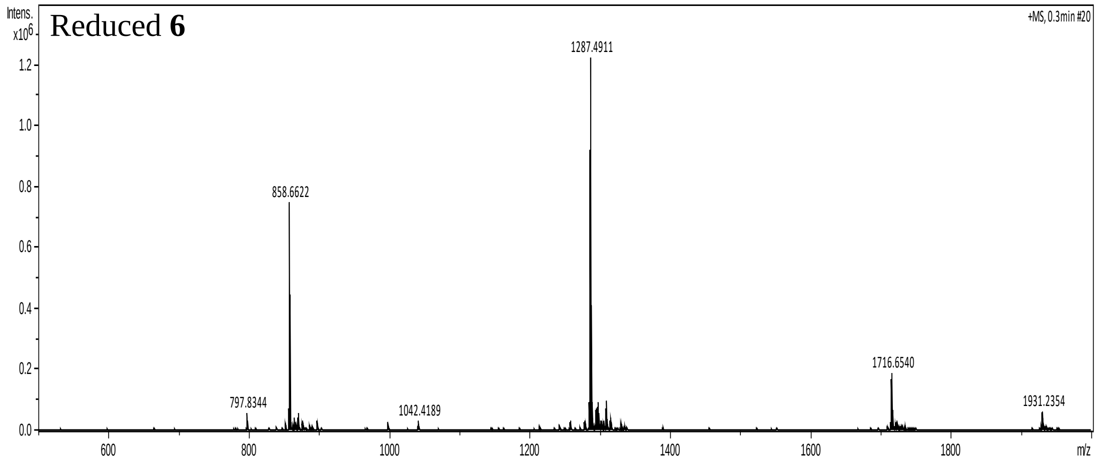
Intens.
x10^6
Reduced 6
+MS, 0.3min #20
1287.4911
858.6622
1716.6540
797.8344
1042.4189
1931.2354
1.2
1.0
0.8
0.6
0.4
0.2
0.0
600
800
1000
1200
1400
1600
1800
m/z

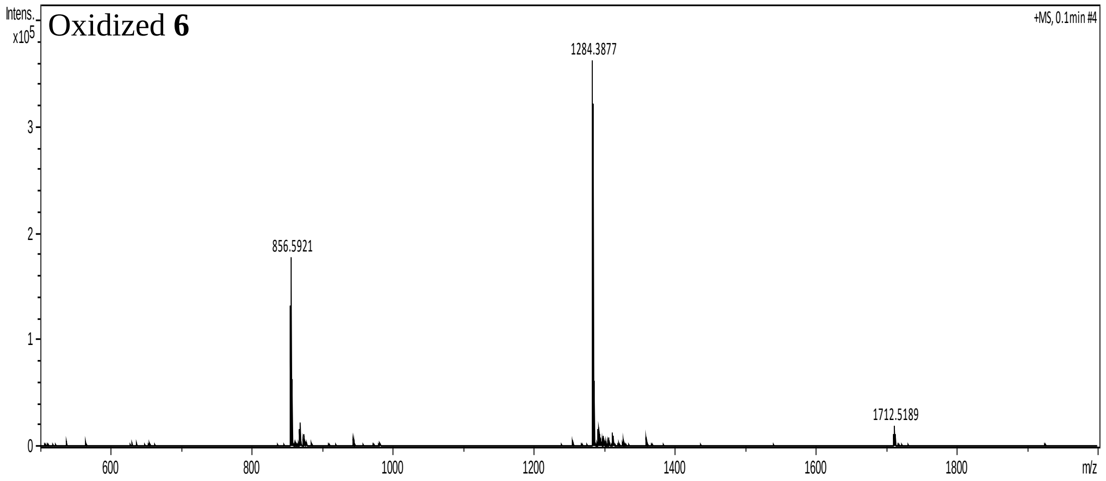
Intens.
x10^5
Oxidized 6
+MS, 0.1min #4
1284.3877
856.5921
1712.5189
3
2
1
0
600
800
1000
1200
1400
1600
1800
m/z

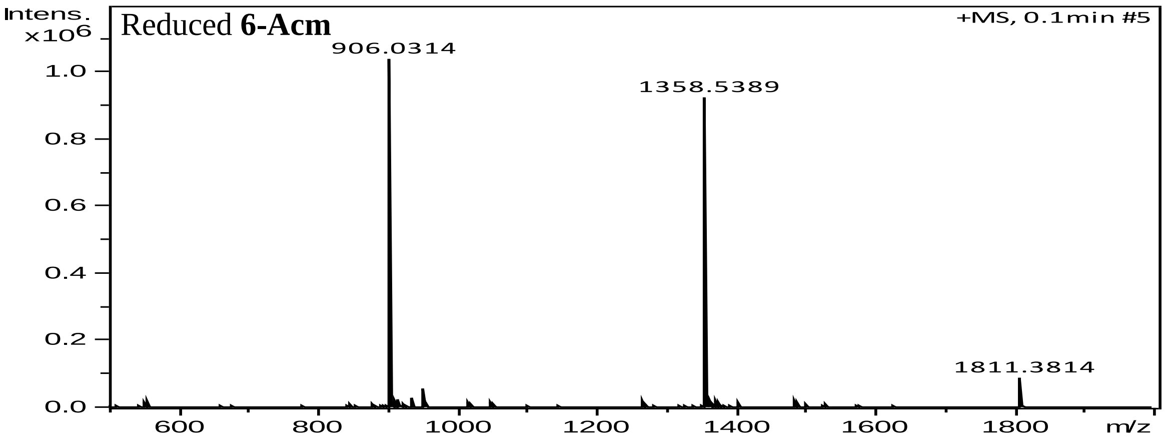
Intens.
x10^6
Reduced 6-Acm
+MS, 0.1min #5
906.0314
1358.5389
1811.3814
1.0
0.8
0.6
0.4
0.2
0.0
600
800
1000
1200
1400
1600
1800
m/z

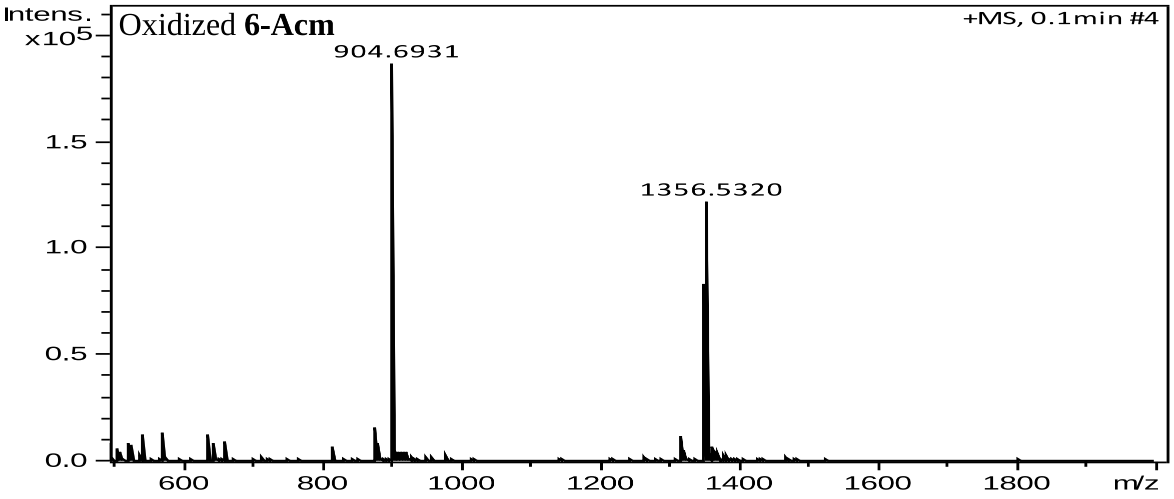
Intens.
x10^5
Oxidized 6-Acm
+MS, 0.1min #4
904.6931
1356.5320
1.5
1.0
0.5
0.0
600
800
1000
1200
1400
1600
1800
m/z

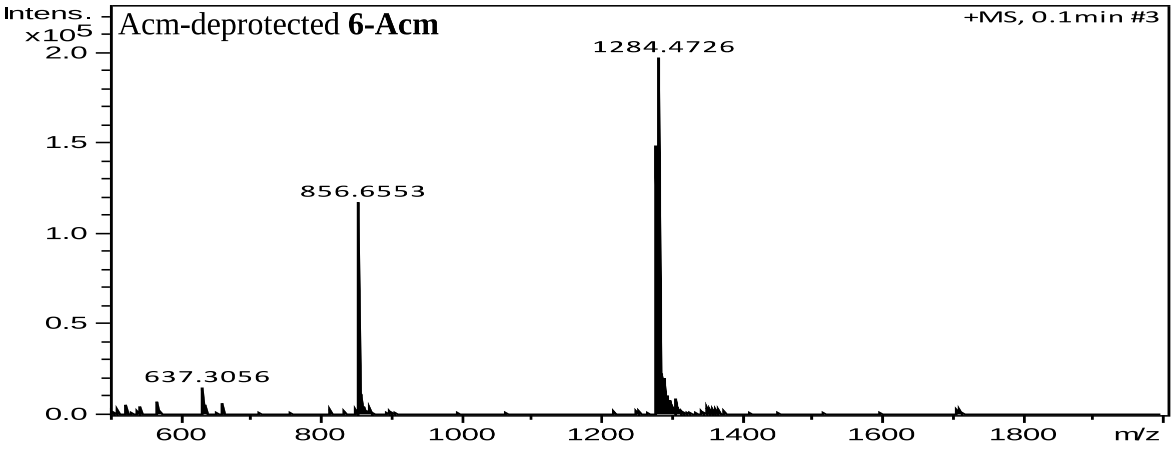
Intens.
x10^5
Acm-deprotected 6-Acm
+MS, 0.1min #3
1284.4726
856.6553
637.3056
2.0
1.5
1.0
0.5
0.0
600
800
1000
1200
1400
1600
1800
m/z

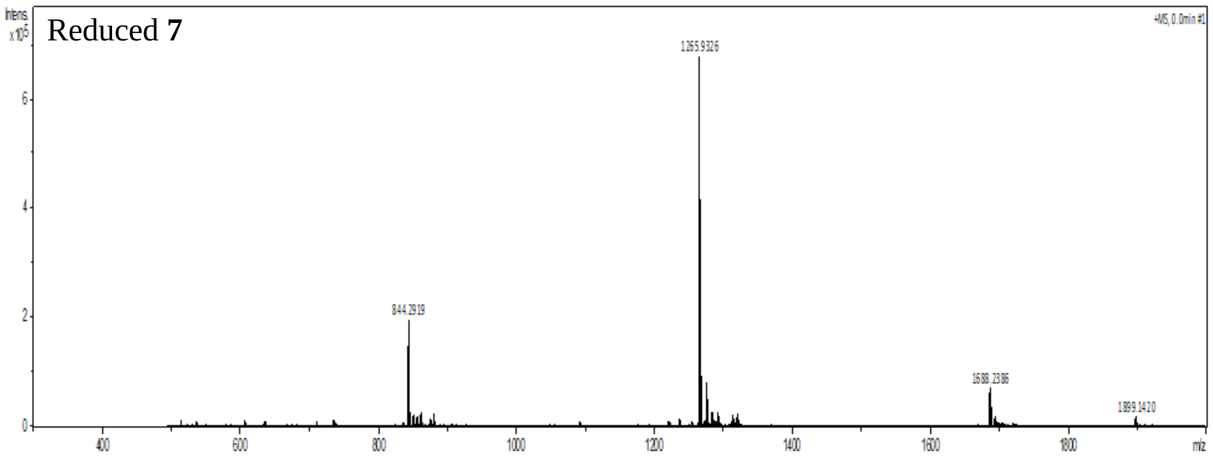
Reduced 7
Intens.
x10^5
+MS, 0.0min #1
1265.9326
844.2919
1688.2386
1899.1420
m/z

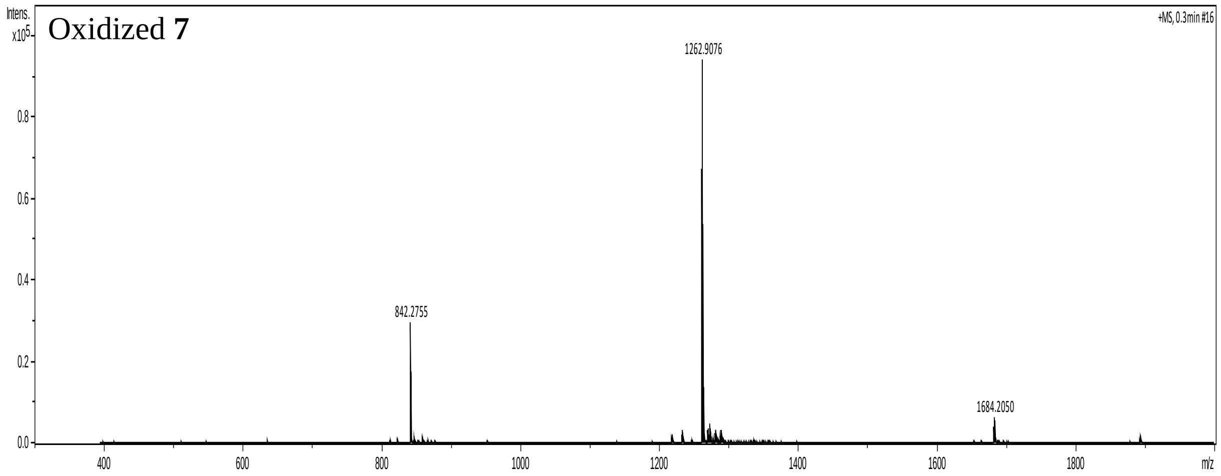
Oxidized 7
Intens.
x10^5
+MS, 0.3min #16
1262.9076
842.2755
1684.2050
m/z

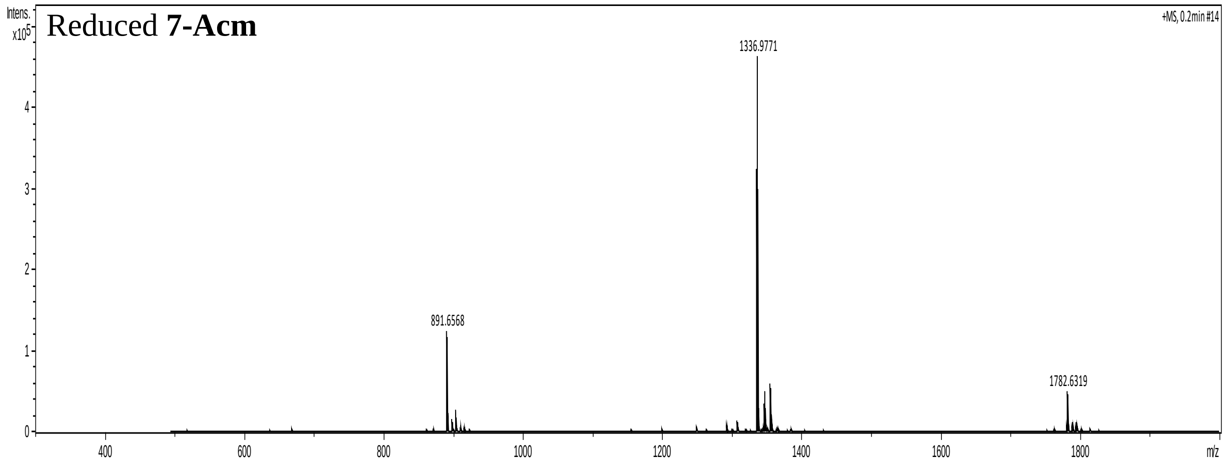
Reduced 7-Acm
Intens.
x10^5
+MS, 0.2min #14
1336.9771
891.6568
1782.6319
m/z

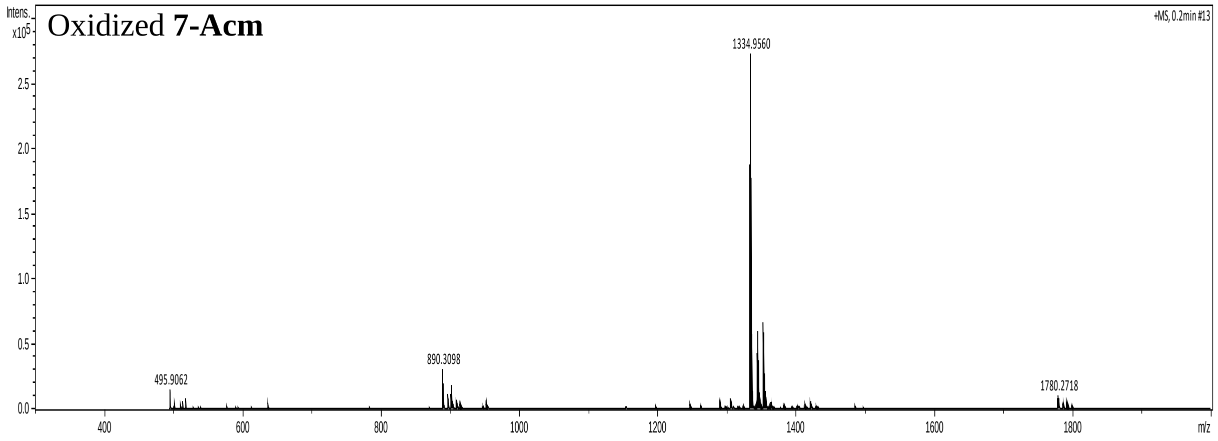
Oxidized 7-Acm
Intens.
x10^5
+MS, 0.2min #13
1334.9560
890.3098
495.9062
1780.2718
2.5
2.0
1.5
1.0
0.5
0.0
400
600
800
1000
1200
1400
1600
1800
m/z

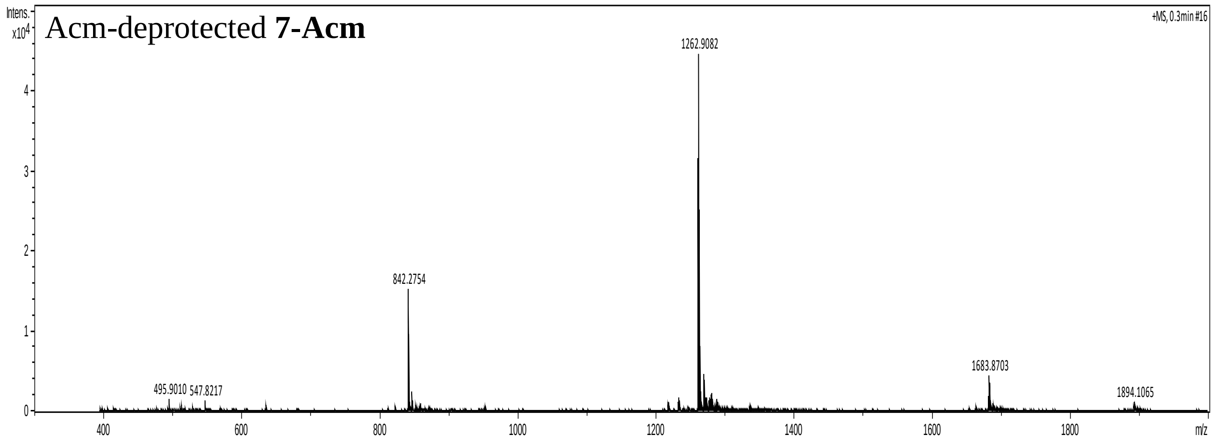
Acm-deprotected 7-Acm
Intens.
x10^4
+MS, 0.3min #16
1262.9082
842.2754
1683.8703
495.9010
547.8217
1894.1065
4
3
2
1
0
400
600
800
1000
1200
1400
1600
1800
m/z

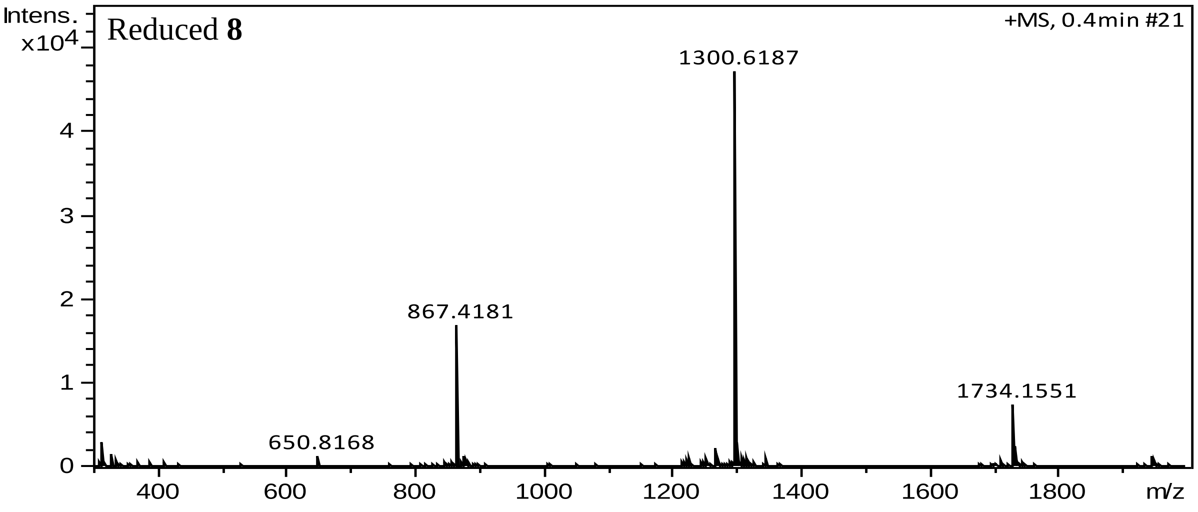
Reduced 8
Intens.
x10^4
+MS, 0.4min #21
1300.6187
867.4181
1734.1551
650.8168
4
3
2
1
0
400
600
800
1000
1200
1400
1600
1800
m/z

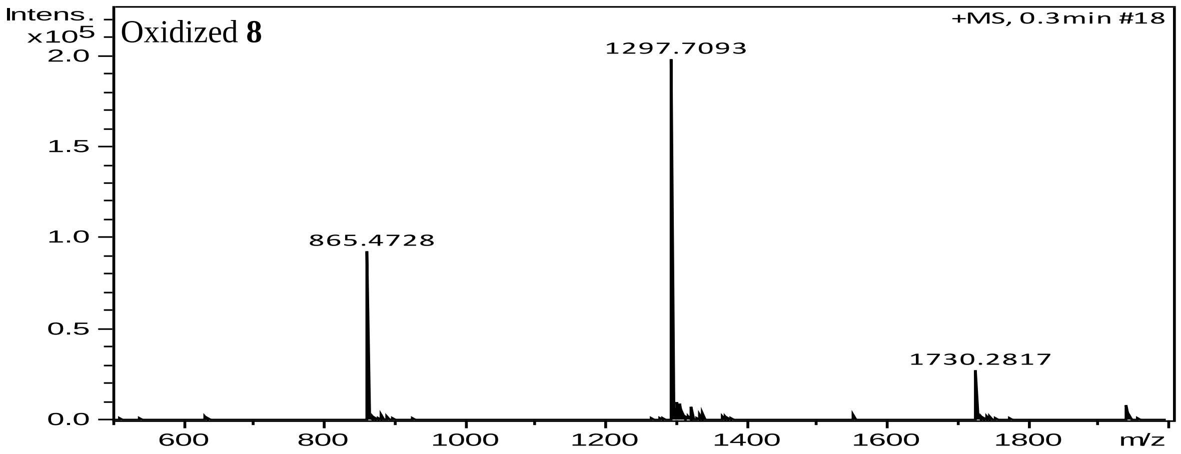
Oxidized 8
+MS, 0.3min #18
Intens. x10^5
865.4728
1297.7093
1730.2817
m/z

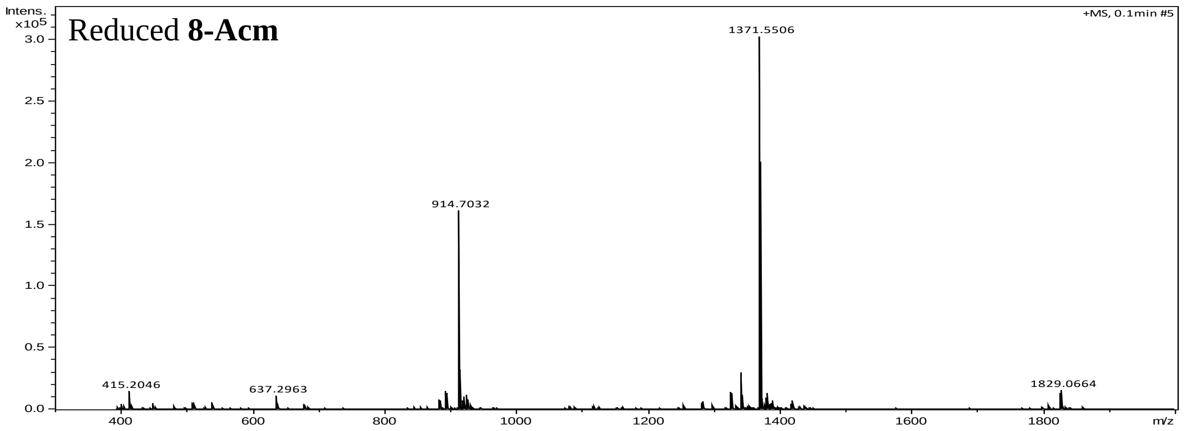
Reduced 8-Acm
+MS, 0.1min #5
Intens. x10^5
415.2046
637.2963
914.7032
1371.5506
1829.0664
m/z

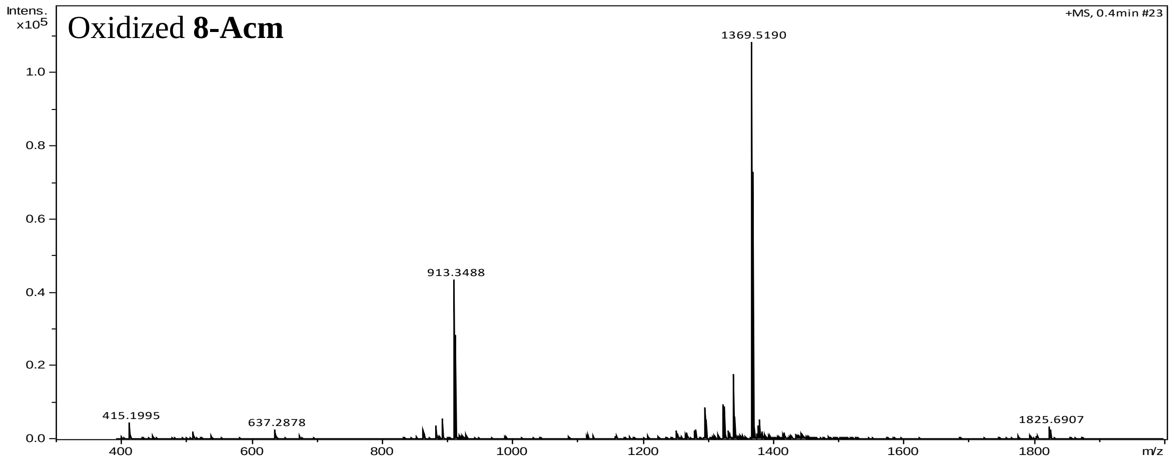
Oxidized 8-Acm
+MS, 0.4min #23
Intens. x10^5
415.1995
637.2878
913.3488
1369.5190
1825.6907
m/z

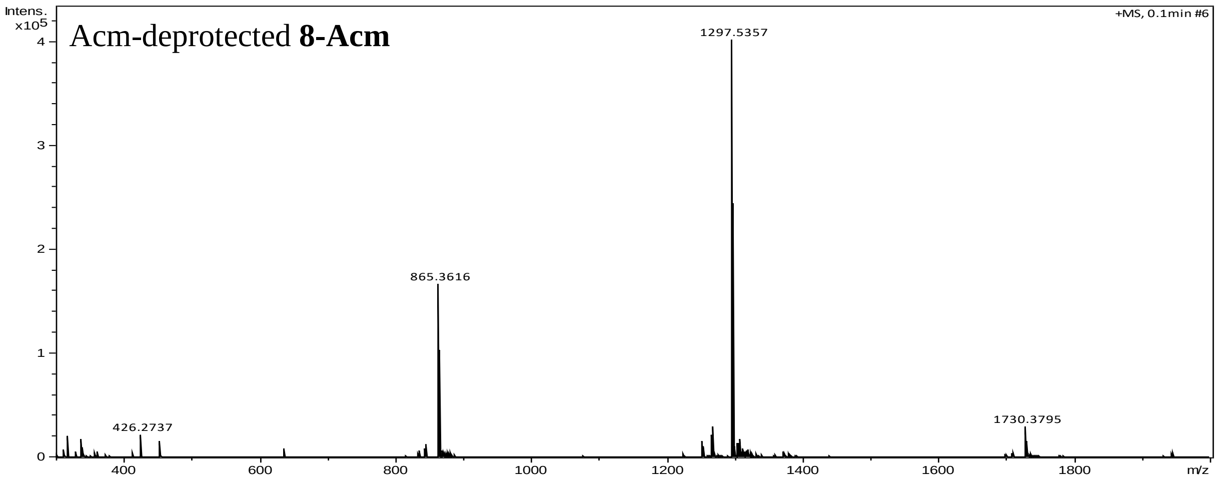
Intens.
x10^5
Acm-deprotected 8-Acm
+MS, 0.1min #6
1297.5357
865.3616
426.2737
1730.3795
m/z

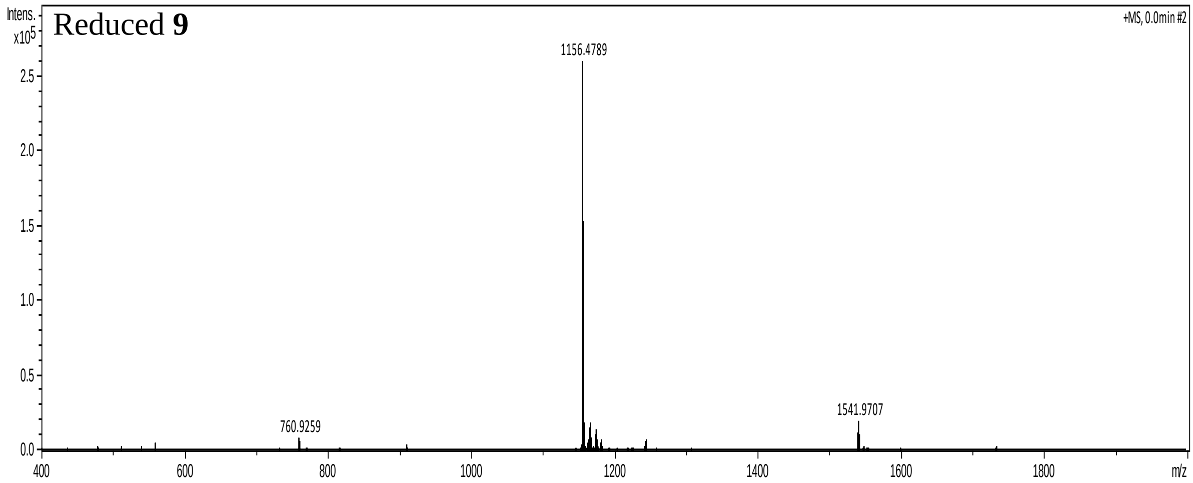
Intens.
x10^5
Reduced 9
+MS, 0.0min #2
1156.4789
760.9259
1541.9707
m/z

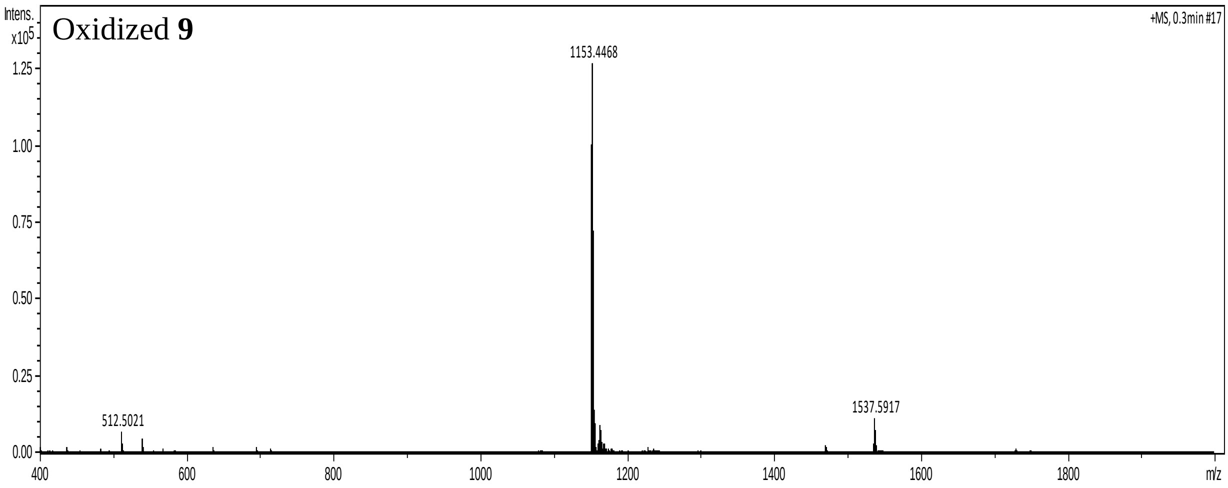
Intens.
x10^5
Oxidized 9
+MS, 0.3min #17
1153.4468
512.5021
1537.5917
m/z

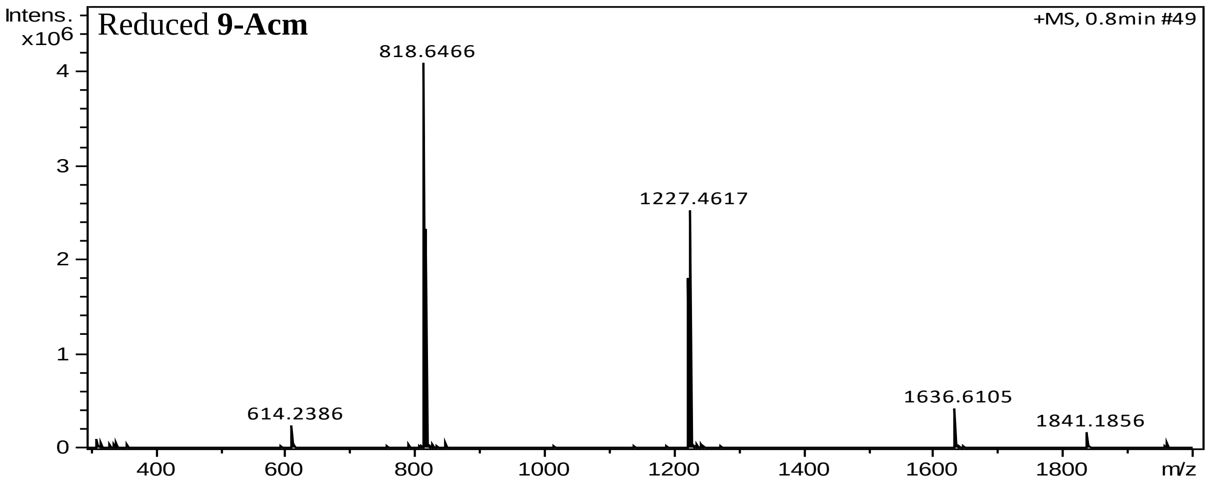
Intens.
x10^6
Reduced 9-Acm
+MS, 0.8min #49
818.6466
1227.4617
614.2386
1636.6105
1841.1856
m/z

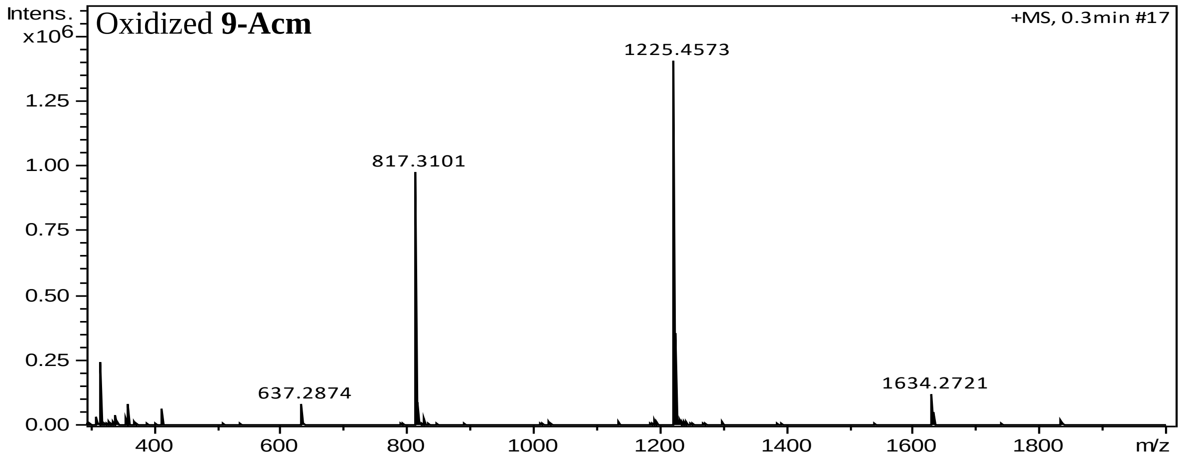
Intens.
x10^6
Oxidized 9-Acm
+MS, 0.3min #17
1225.4573
817.3101
637.2874
1634.2721
m/z

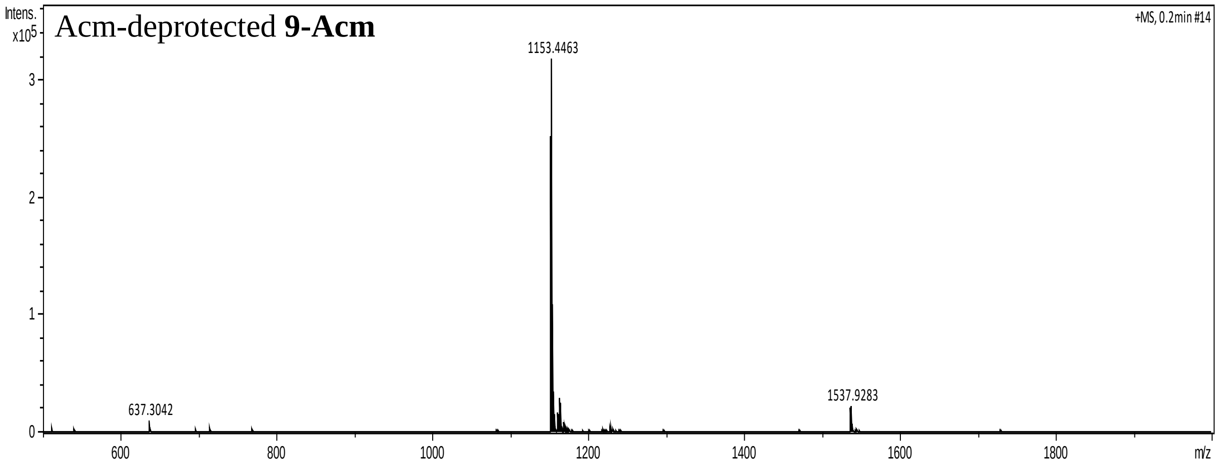
Intens.
x10^5
Acm-deprotected 9-Acm
+MS, 0.2min #14
1153.4463
637.3042
1537.9283
m/z

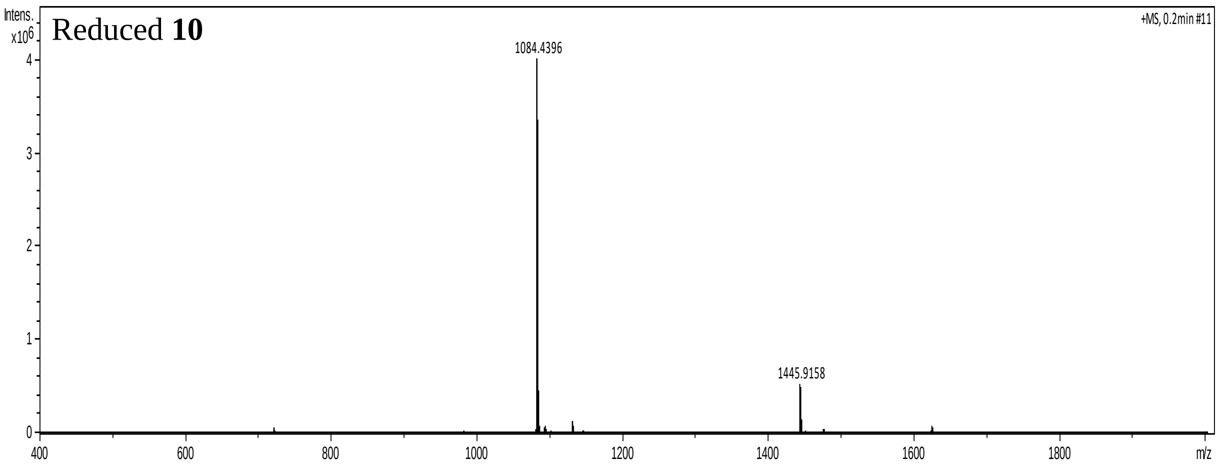
Intens.
x10^6
Reduced 10
+MS, 0.2min #11
1084.4396
1445.9158
0
1
2
3
4
400
600
800
1000
1200
1400
1600
1800
m/z

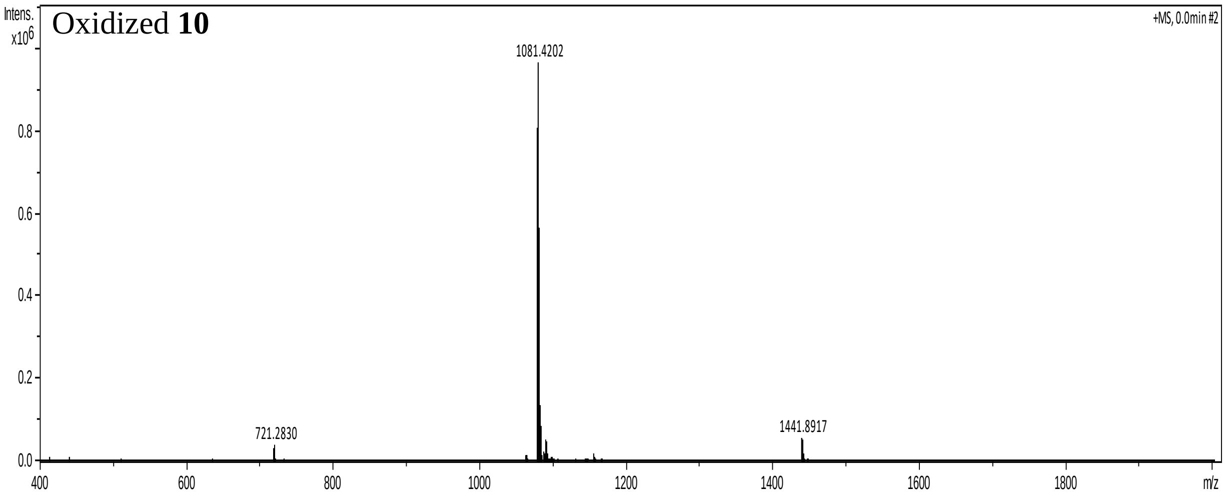
Intens.
x10^6
Oxidized 10
+MS, 0.0min #2
1081.4202
721.2830
1441.8917
0.0
0.2
0.4
0.6
0.8
400
600
800
1000
1200
1400
1600
1800
m/z

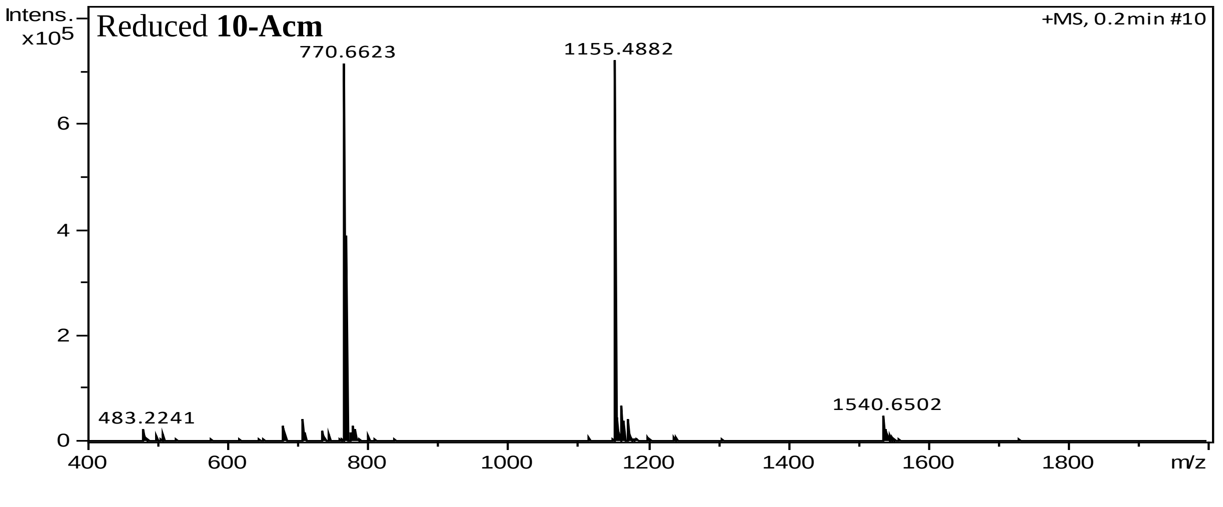
Intens.
x10^5
Reduced 10-Acm
+MS, 0.2min #10
770.6623
1155.4882
483.2241
1540.6502
0
2
4
6
400
600
800
1000
1200
1400
1600
1800
m/z

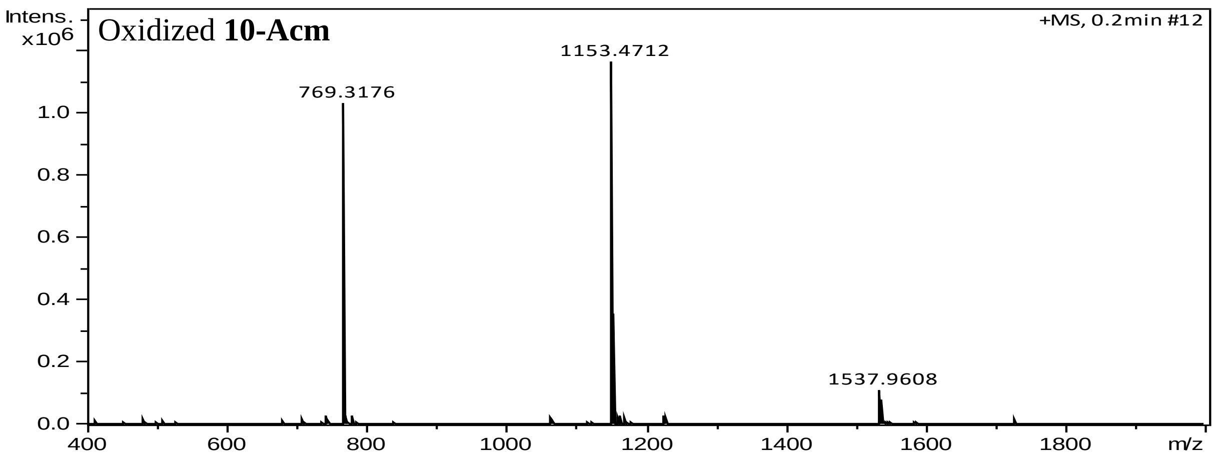
Oxidized 10-Acm
Intens.
x10^6
+MS, 0.2min #12
769.3176
1153.4712
1537.9608
m/z

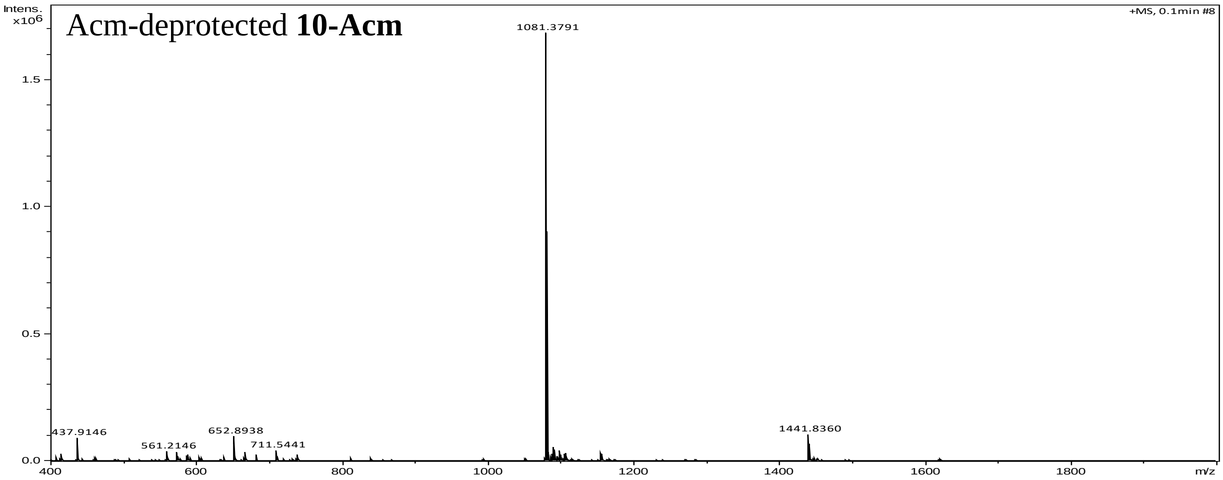
Acm-deprotected 10-Acm
Intens.
x10^6
+MS, 0.1min #8
1081.3791
437.9146
561.2146
652.8938
711.5441
1441.8360
m/z

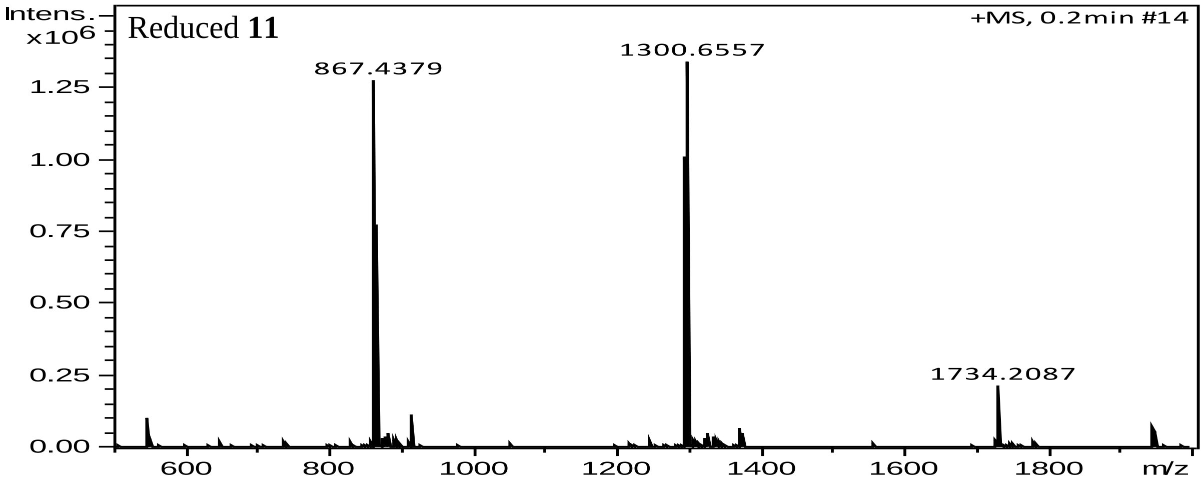
Reduced 11
Intens.
x10^6
+MS, 0.2min #14
867.4379
1300.6557
1734.2087
m/z

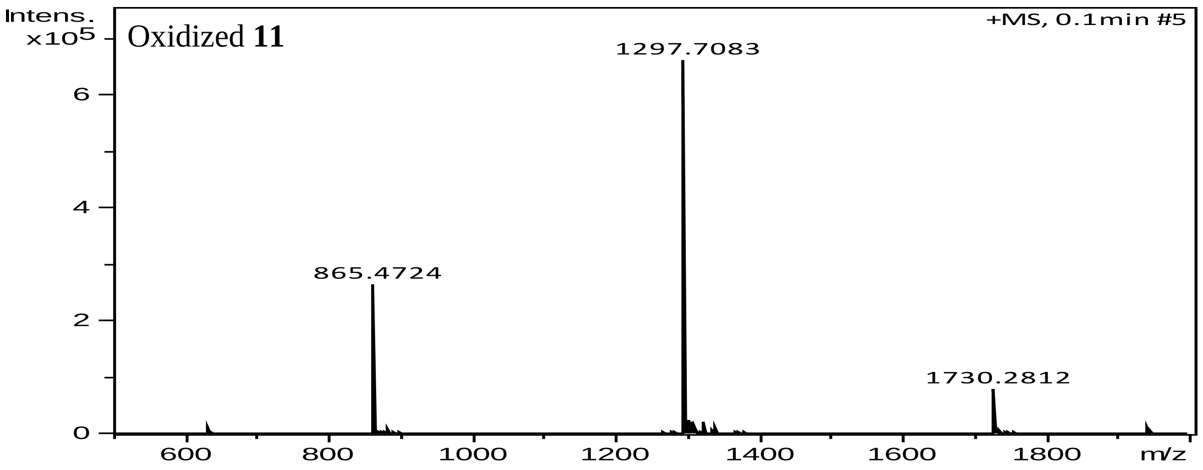
Intens.
x10^5
Oxidized 11
+MS, 0.1min #5
1297.7083
865.4724
1730.2812
0
2
4
6
600
800
1000
1200
1400
1600
1800
m/z

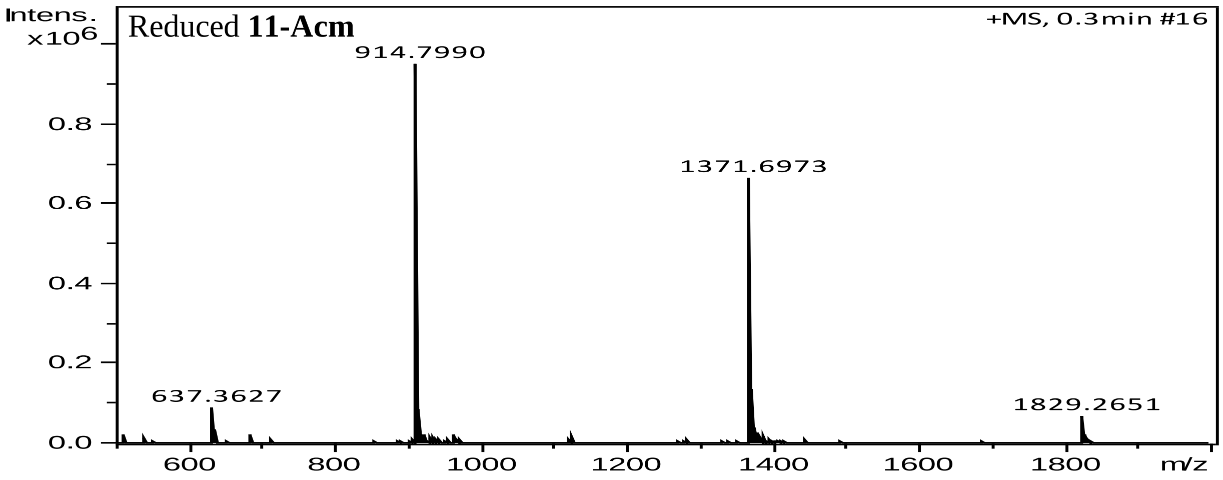
Intens.
x10^6
Reduced 11-Acm
+MS, 0.3min #16
914.7990
1371.6973
637.3627
1829.2651
0.0
0.2
0.4
0.6
0.8
600
800
1000
1200
1400
1600
1800
m/z

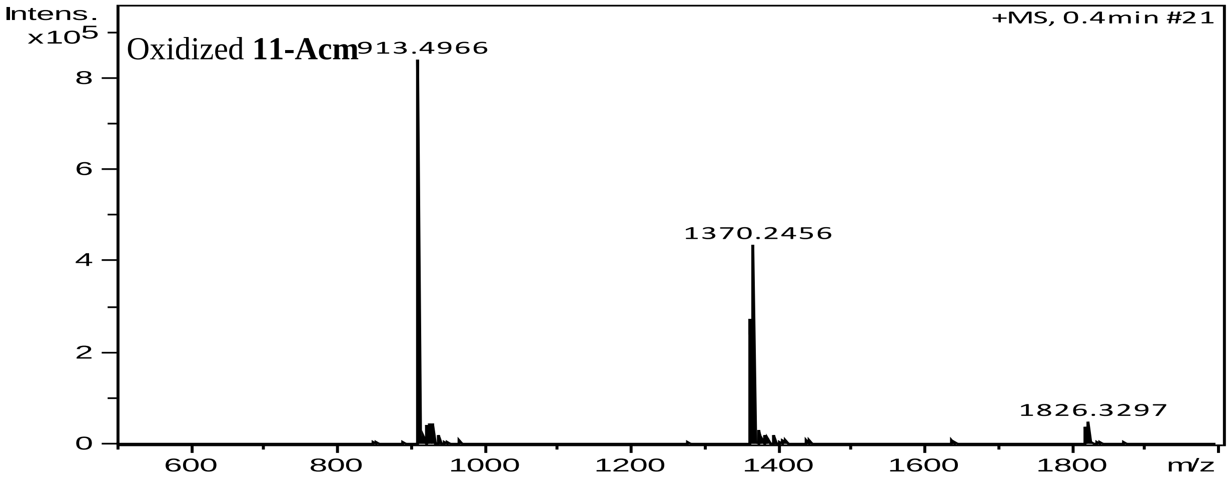
Intens.
x10^5
Oxidized 11-Acm
+MS, 0.4min #21
913.4966
1370.2456
1826.3297
0
2
4
6
8
600
800
1000
1200
1400
1600
1800
m/z

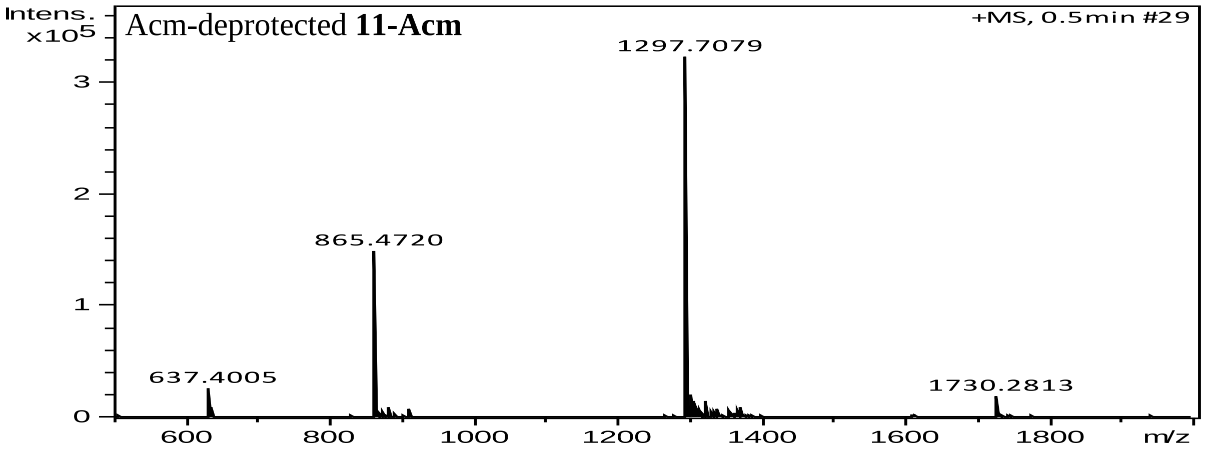
Intens.
x10^5
Acm-deprotected 11-Acm
+MS, 0.5min #29
1297.7079
865.4720
637.4005
1730.2813
m/z

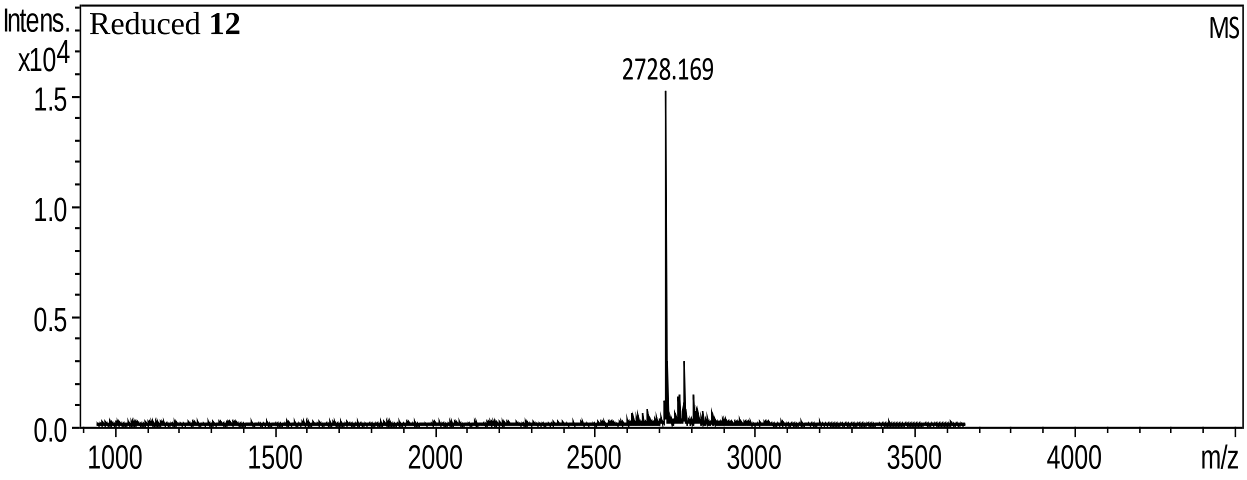
Intens.
x10^4
Reduced 12
MS
2728.169
m/z

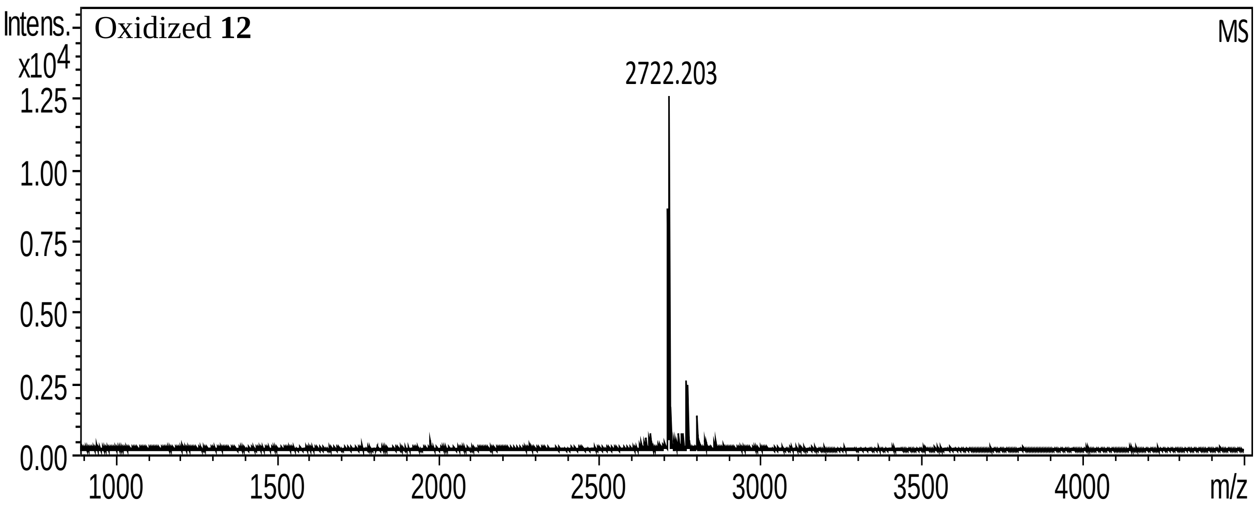
Intens.
x10^4
Oxidized 12
MS
2722.203
m/z

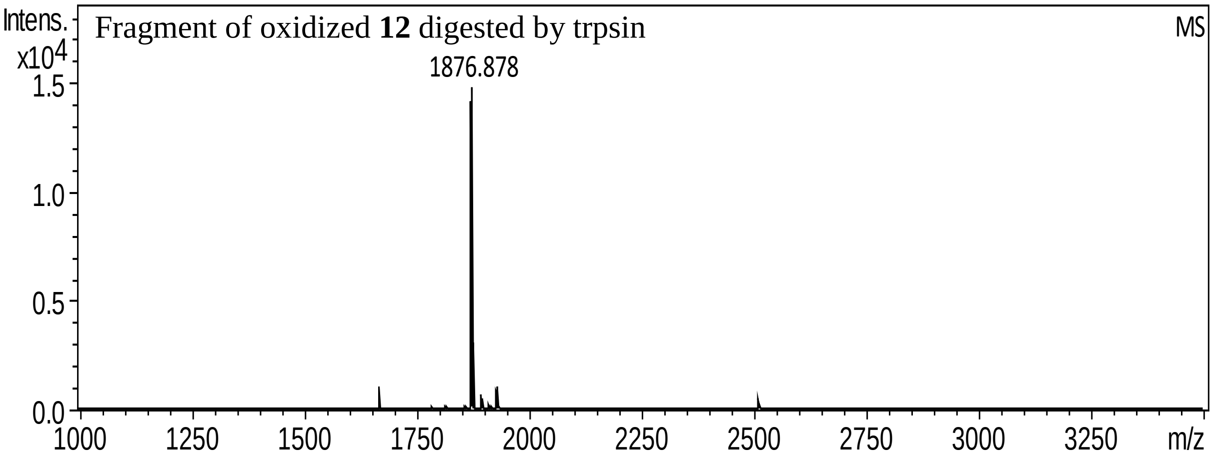
Intens.
x10^4
Fragment of oxidized 12 digested by trpsin
MS
1876.878
1.5
1.0
0.5
0.0
1000
1250
1500
1750
2000
2250
2500
2750
3000
3250
m/z

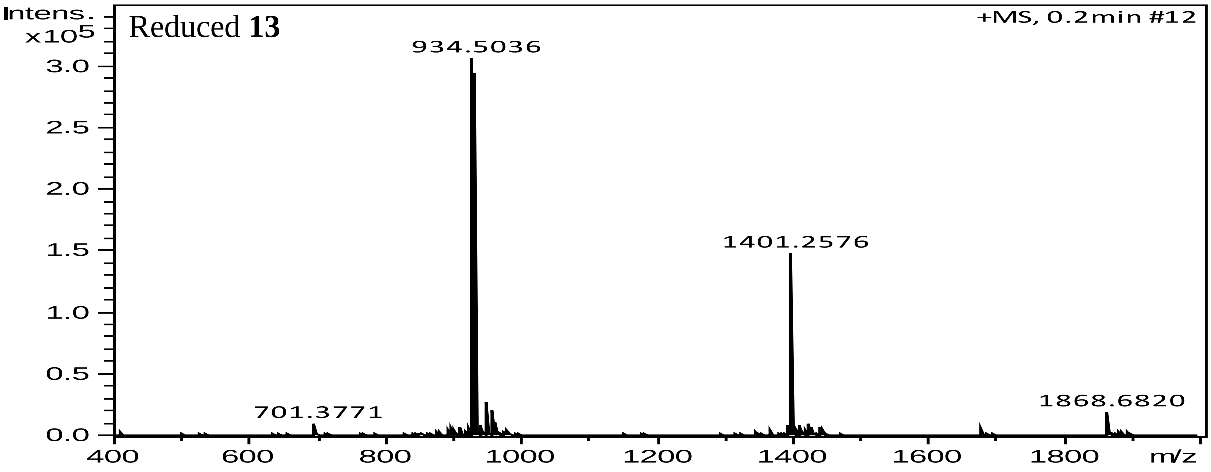
Intens.
x10^5
Reduced 13
+MS, 0.2min #12
934.5036
1401.2576
701.3771
1868.6820
3.0
2.5
2.0
1.5
1.0
0.5
0.0
400
600
800
1000
1200
1400
1600
1800
m/z

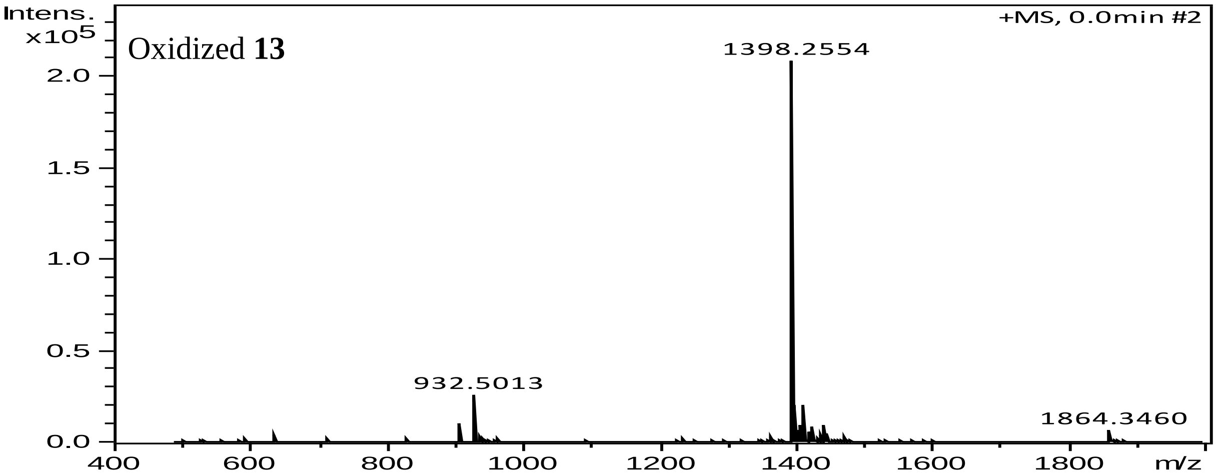
Intens.
x10^5
Oxidized 13
+MS, 0.0min #2
1398.2554
932.5013
1864.3460
2.0
1.5
1.0
0.5
0.0
400
600
800
1000
1200
1400
1600
1800
m/z

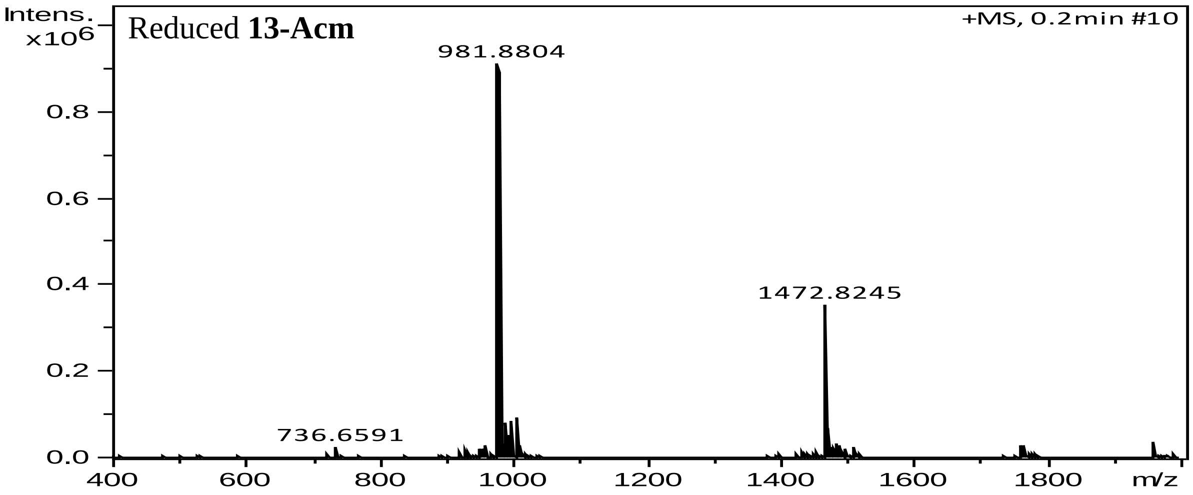
Intens.
x10^6
Reduced 13-Acm
+MS, 0.2min #10
981.8804
1472.8245
736.6591
0.8
0.6
0.4
0.2
0.0
400
600
800
1000
1200
1400
1600
1800
m/z

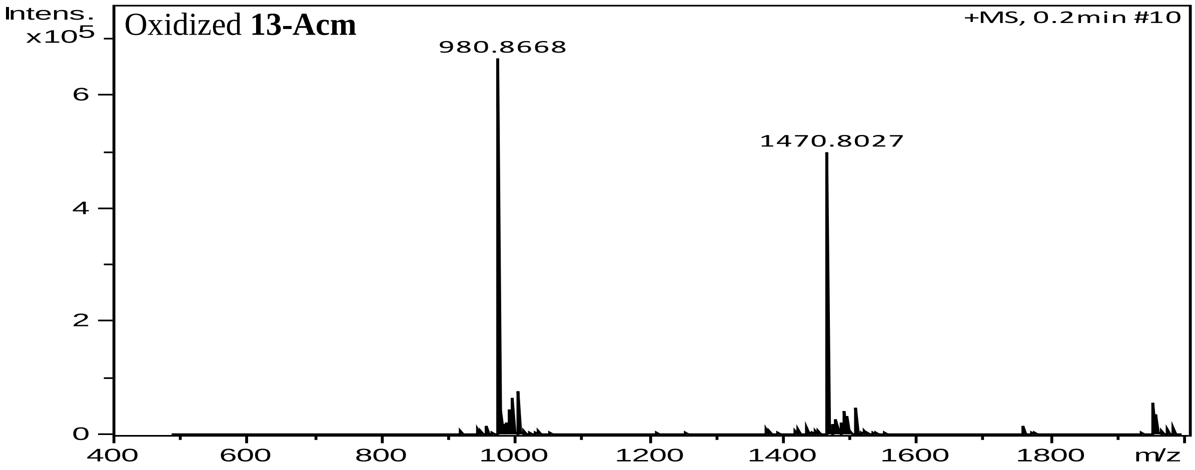
Intens.
x10^5
Oxidized 13-Acm
+MS, 0.2min #10
980.8668
1470.8027
6
4
2
0
400
600
800
1000
1200
1400
1600
1800
m/z

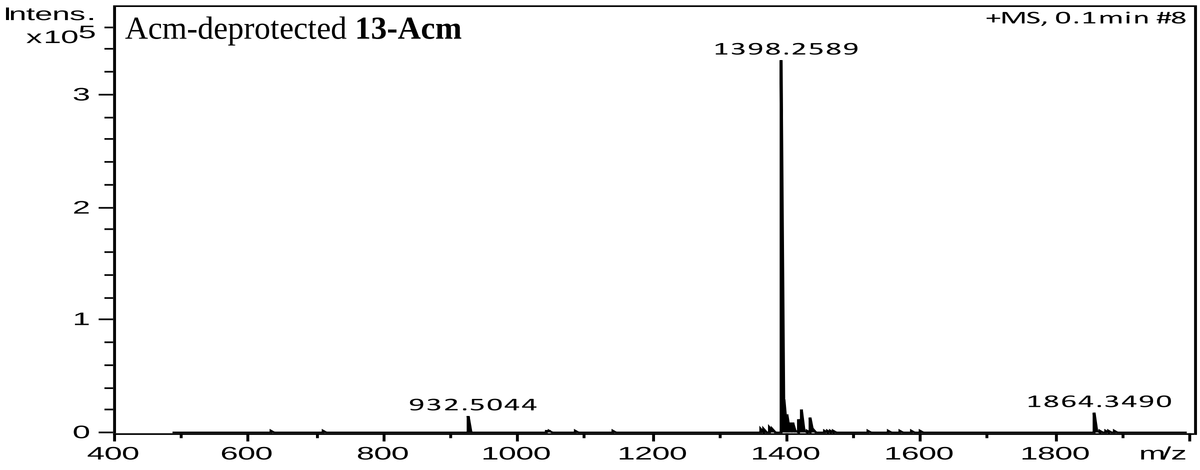
Intens.
x10^5
Acm-deprotected 13-Acm
+MS, 0.1min #8
1398.2589
932.5044
1864.3490
3
2
1
0
400
600
800
1000
1200
1400
1600
1800
m/z

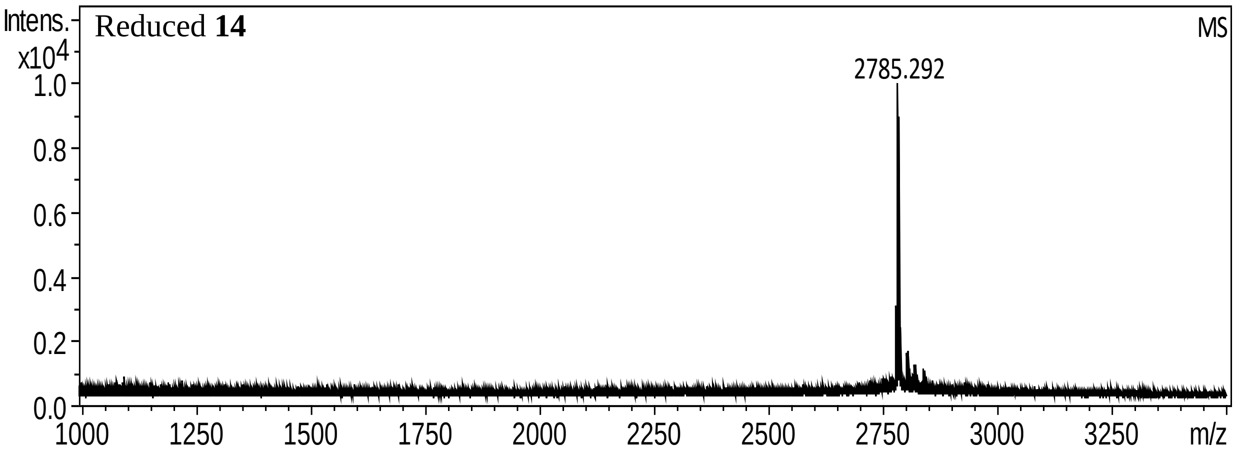
Intens.
x10^4
Reduced 14
MS
2785.292
1.0
0.8
0.6
0.4
0.2
0.0
1000
1250
1500
1750
2000
2250
2500
2750
3000
3250
m/z

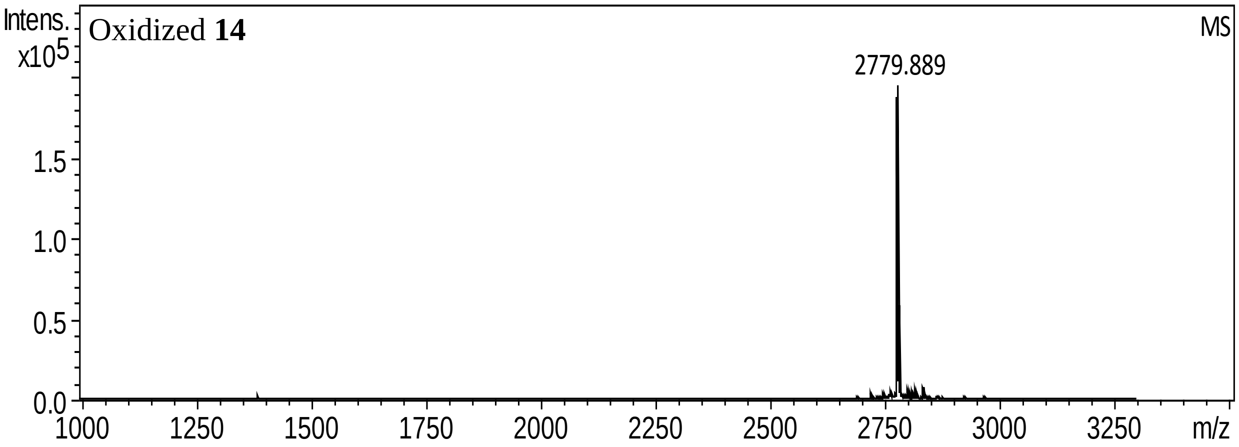
Intens.
x10^5
Oxidized 14
MS
2779.889
1.5
1.0
0.5
0.0
1000
1250
1500
1750
2000
2250
2500
2750
3000
3250
m/z

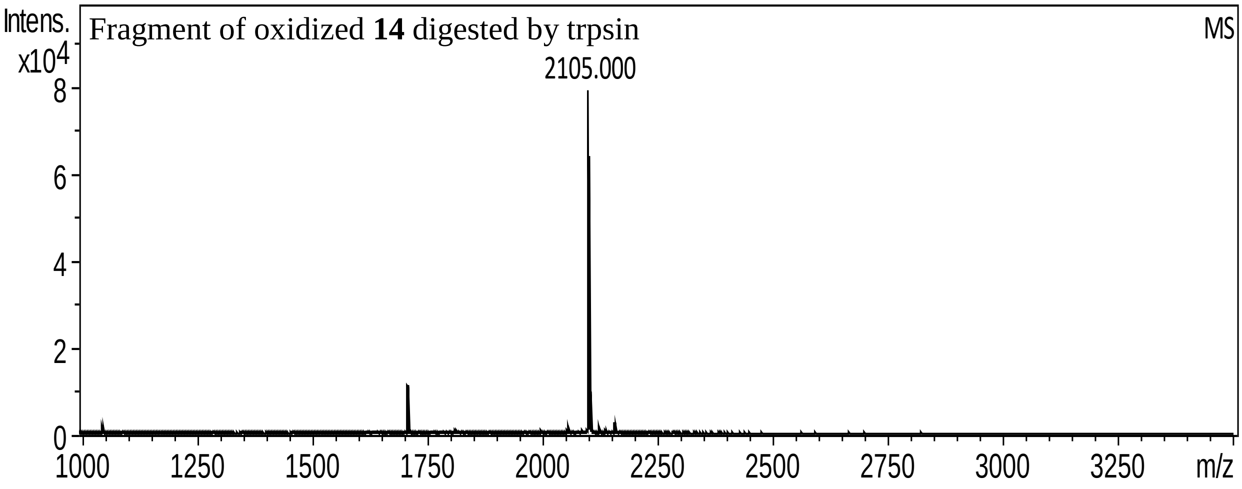
Intens.
x10^4
Fragment of oxidized 14 digested by trpsin
MS
2105.000
8
6
4
2
0
1000
1250
1500
1750
2000
2250
2500
2750
3000
3250
m/z

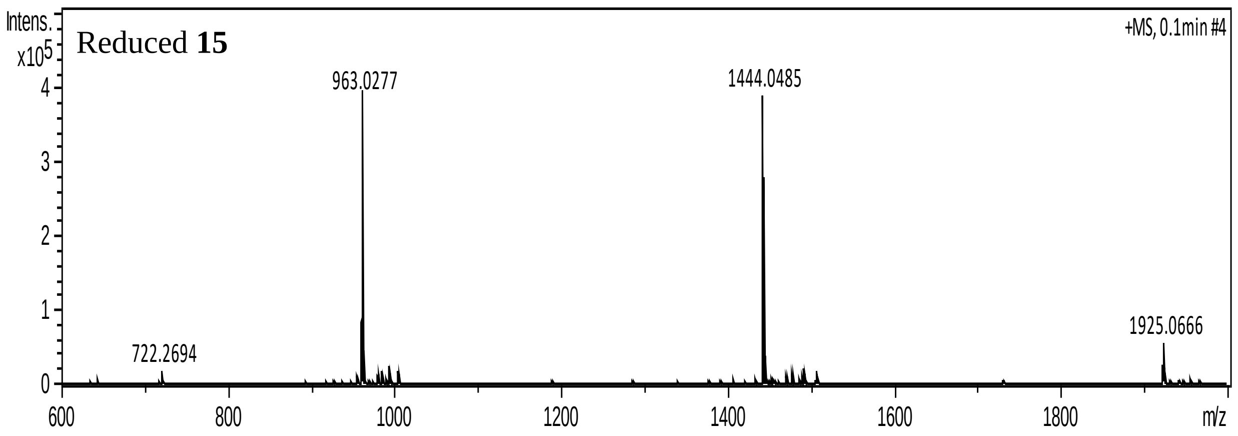
Intens.
x10^5
Reduced 15
+MS, 0.1min #4
963.0277
1444.0485
1925.0666
722.2694
4
3
2
1
0
600
800
1000
1200
1400
1600
1800
m/z

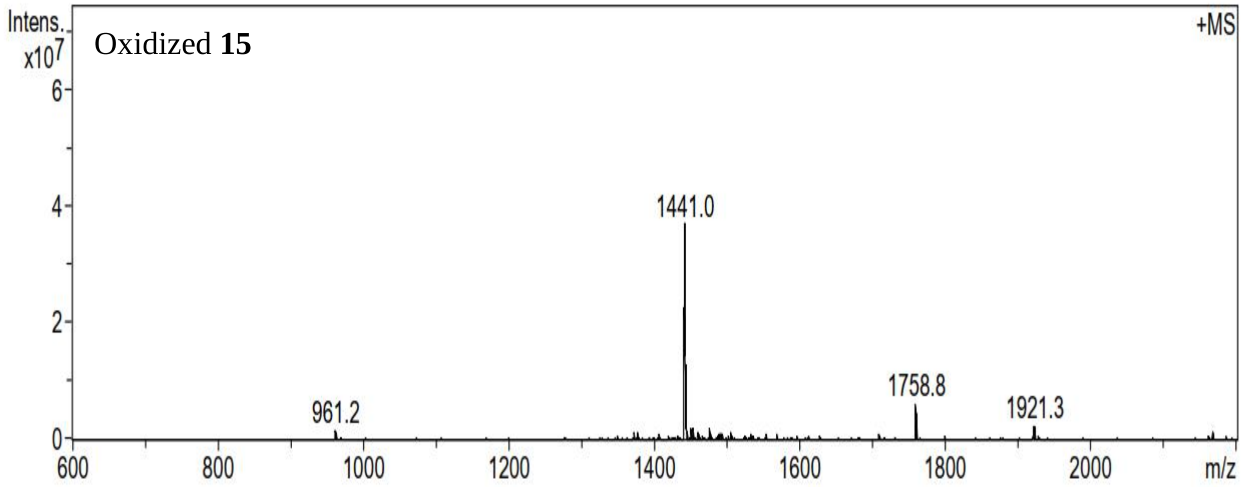
Intens.
x10^7
Oxidized 15
+MS
1441.0
1758.8
1921.3
961.2
6
4
2
0
600
800
1000
1200
1400
1600
1800
2000
m/z

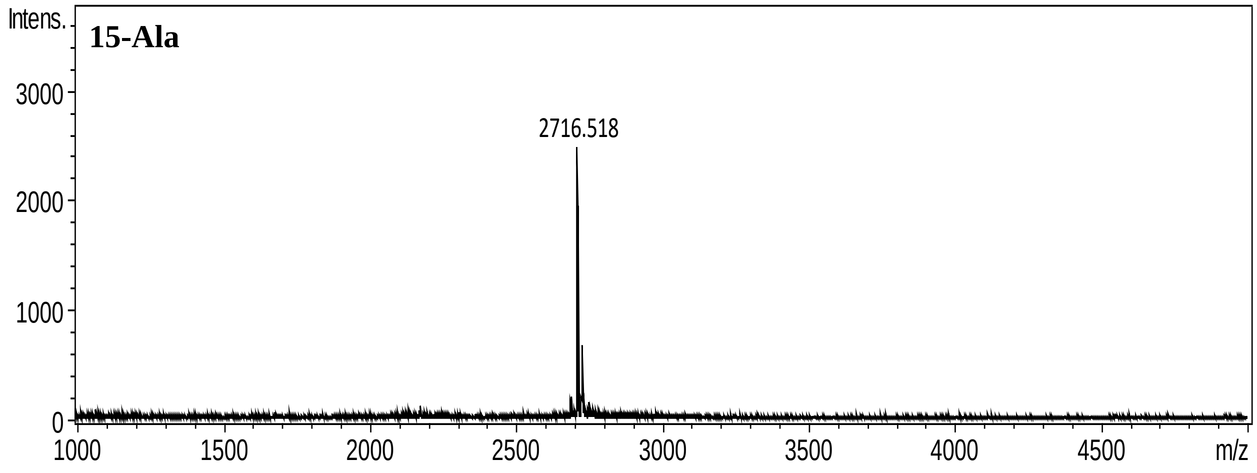
Intens.
15-Ala
2716.518
3000
2000
1000
0
1000
1500
2000
2500
3000
3500
4000
4500
m/z

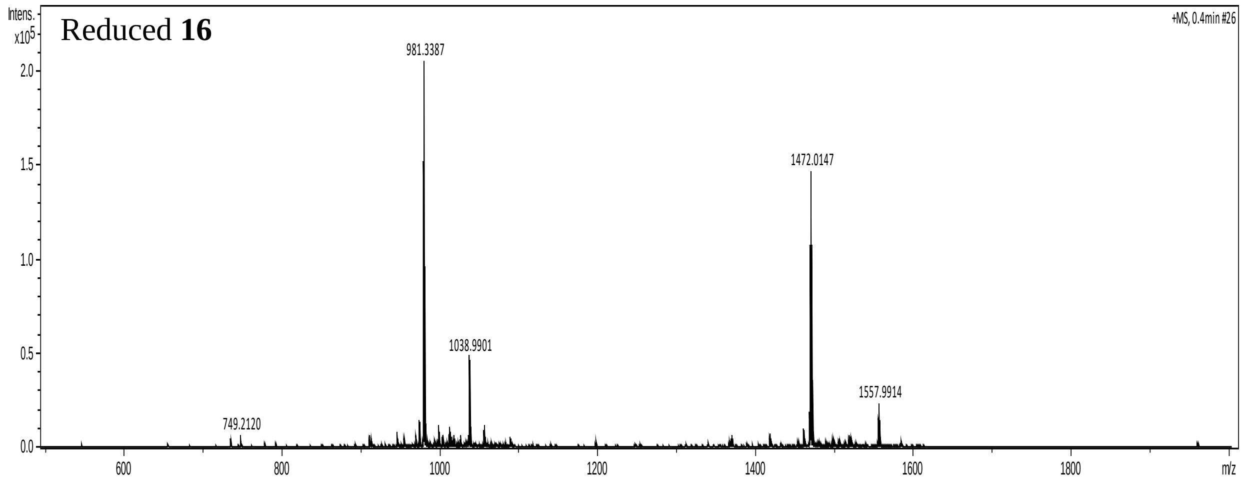
Reduced 16
Intens. x10^5
+MS, 0.4min #26
981.3387
1472.0147
1038.9901
1557.9914
749.2120
m/z

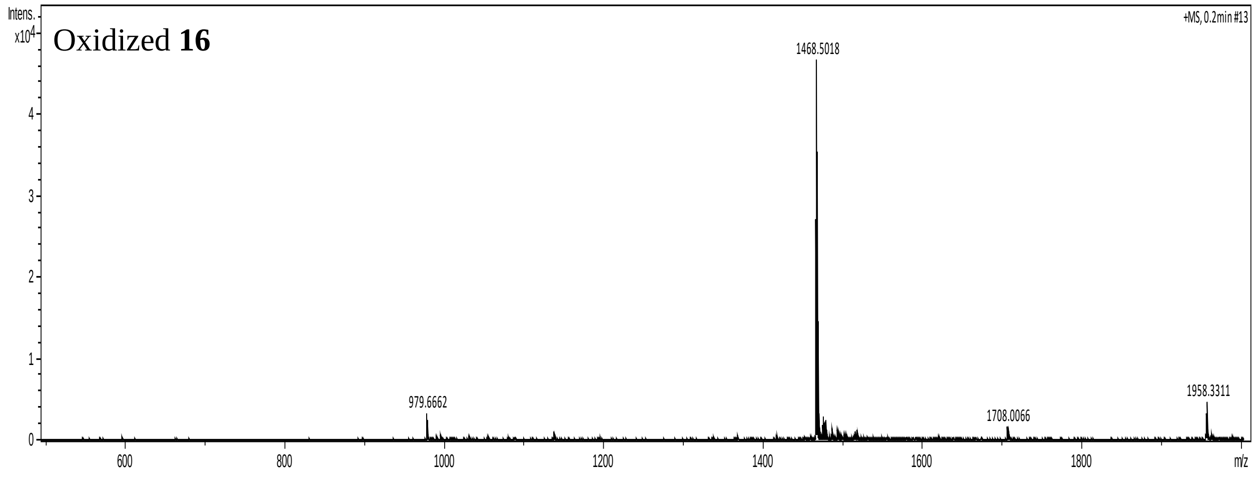
Oxidized 16
Intens. x10^4
+MS, 0.2min #13
1468.5018
979.6662
1708.0066
1958.3311
m/z

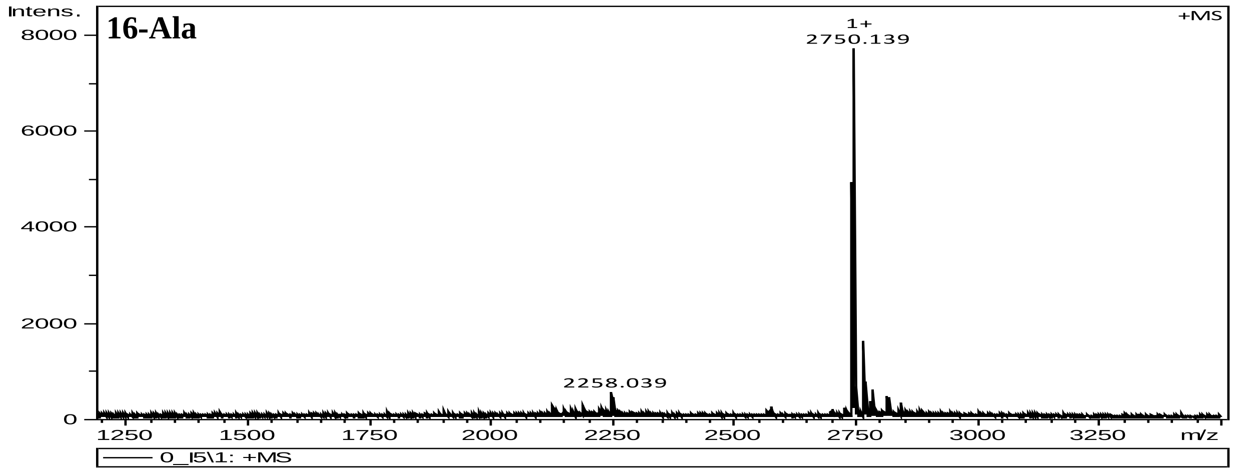
16-Ala
Intens.
+MS
1+
2750.139
2258.039
m/z
0_I5\1: +MS

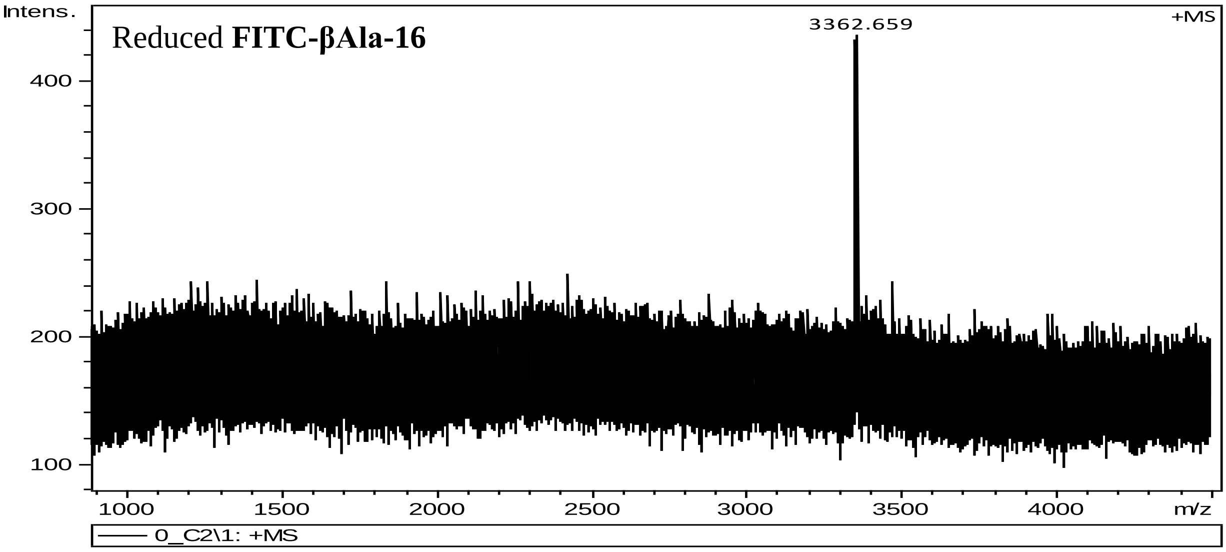
Intens.
Reduced **FITC-βAla-16**
3362.659
+MS
400
300
200
100
1000
1500
2000
2500
3000
3500
4000
m/z
0_C2\1: +MS

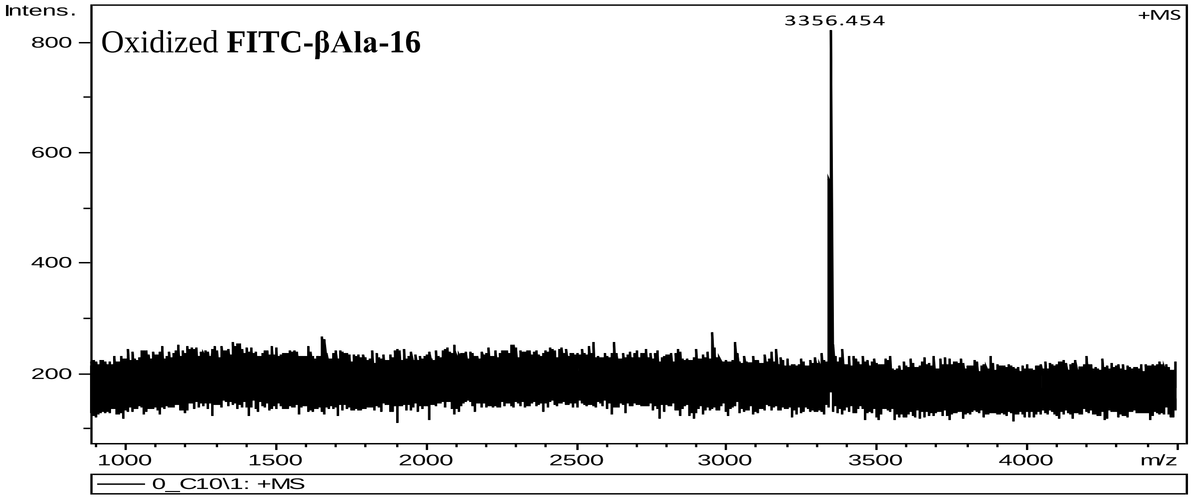
Intens.
Oxidized **FITC-βAla-16**
3356.454
+MS
800
600
400
200
1000
1500
2000
2500
3000
3500
4000
m/z
0_C10\1: +MS

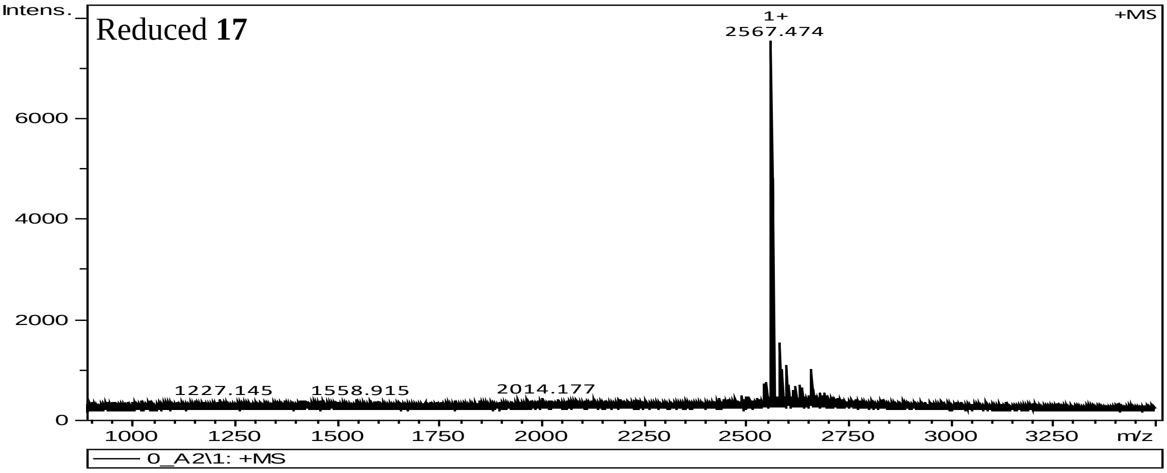
Intens.
Reduced **17**
1+
2567.474
+MS
6000
4000
2000
0
1227.145
1558.915
2014.177
1000
1250
1500
1750
2000
2250
2500
2750
3000
3250
m/z
0_A2\1: +MS

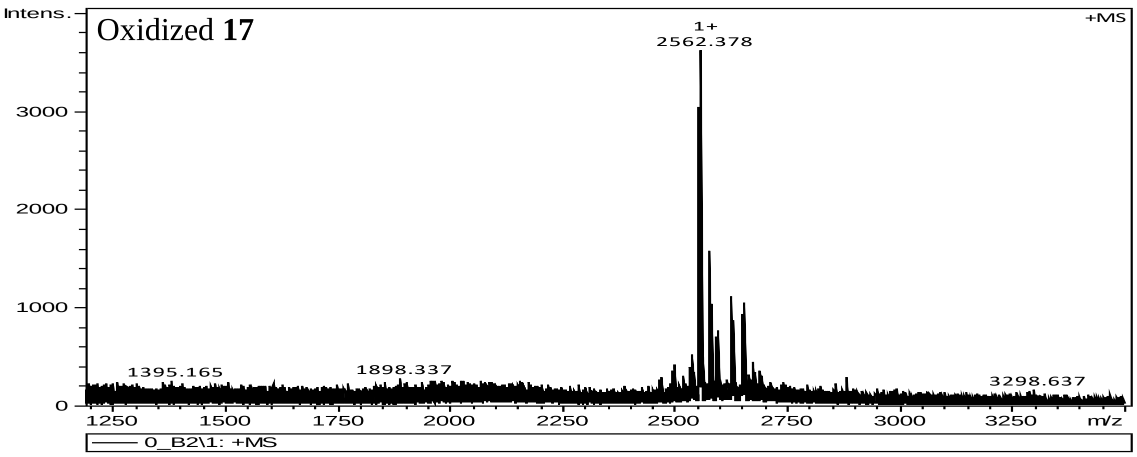
Oxidized 17
Intens.
+MS
1+
2562.378
1395.165
1898.337
3298.637
m/z
0_B2\1: +MS

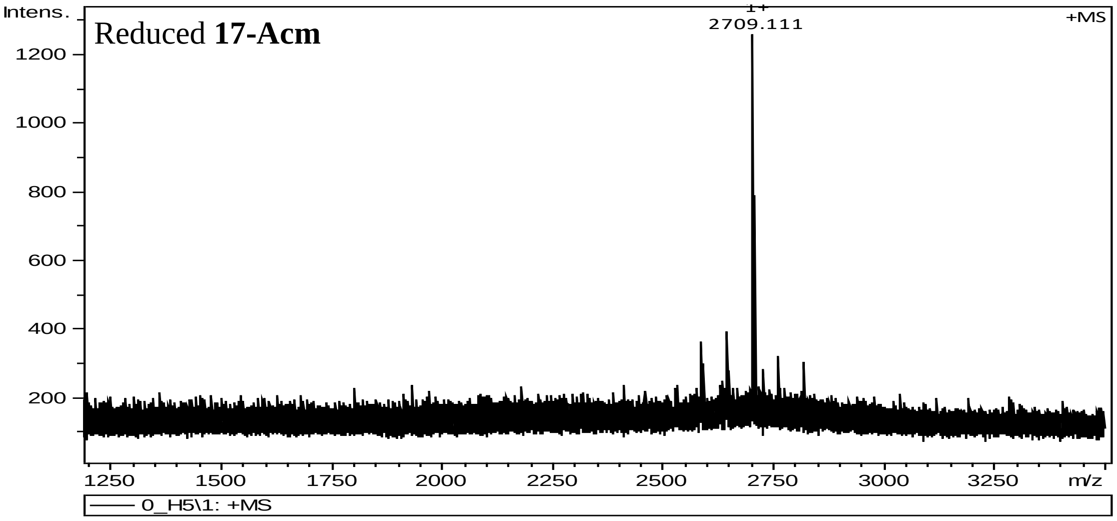
Reduced 17-Acm
Intens.
+MS
1+
2709.111
m/z
0_H5\1: +MS

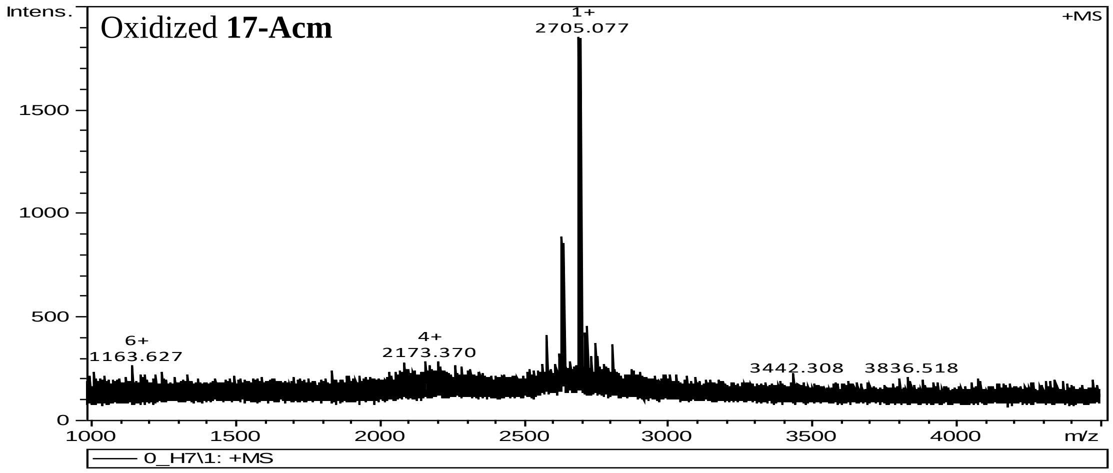
Oxidized 17-Acm
Intens.
+MS
1+
2705.077
6+
1163.627
4+
2173.370
3442.308
3836.518
m/z
0_H7\1: +MS

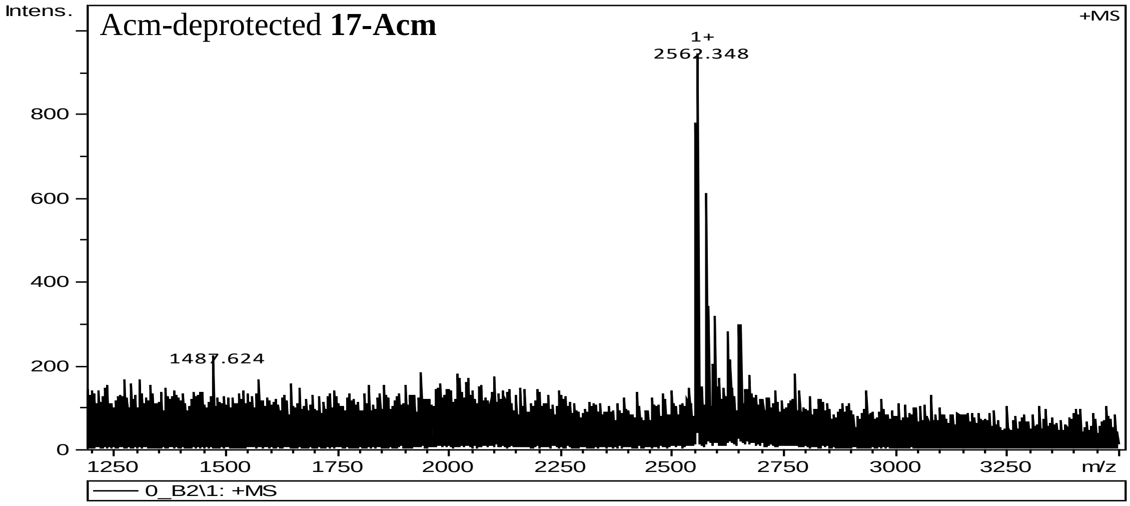

Figure S27. Mass spectra of peptides.

References

1 Lu, S. M. *et al.* Directed disulfide pairing and folding of peptides for the de novo development of multicyclic peptide libraries. *J. Am. Chem. Soc.* **142**, 16285-16291 (2020).

2 Zha, M. R., Lin, P., Yao, H. W., Zhao, Y. B. & Wu, C. L. A phage display-based strategy for the de novo creation of disulfide-constrained and isomer-free bicyclic peptide affinity reagents. *Chem. Commun.* **54**, 4029-4032 (2018).

3 Zheng, X. *et al.* Condensation of 2-((alkylthio)(aryl)methylene)malononitrile with 1,2-aminothiol as a novel bioorthogonal reaction for site-specific protein modification and peptide cyclization. *J. Am. Chem. Soc.* **142**, 5097-5103 (2020).

4 Rebollo, I. R. & Heinis, C. Phage selection of bicyclic peptides. *Methods* **60**, 46-54 (2013).

5 Zheng, X. J., Liu, W. D., Liu, Z. Y., Zhao, Y. B. & Wu, C. L. Biocompatible and rapid cyclization of peptides with 2,4-difluoro-6-hydroxy-1,3,5-benzenetricarbonitrile for the development of cyclic peptide libraries. *Bioconjugate Chem.* **31**, 2085-2091 (2020).

6 Chen, Y. Q. *et al.* The interplay of disulfide bonds, alpha-helicity, and hydrophobic interactions leads to ultrahigh proteolytic stability of peptides. *Biomacromolecules* **16**, 2347-2355 (2015).

7 Delaglio, F. *et al.* NMRPipe - a multidimensional spectral processing system based on UNIX pipes. *J. Biomol. NMR* **6**, 277-293 (1995).

8 Lee, W., Tonelli, M. & Markley, J. L. NMRFAM-SPARKY: enhanced software for biomolecular NMR spectroscopy. *Bioinformatics* **31**, 1325-1327 (2015).

9 Shen, Y. & Bax, A. Protein backbone and sidechain torsion angles predicted from NMR chemical shifts using artificial neural networks. *J. Biomol. NMR* **56**, 227-241 (2013).

10 Rieping, W. *et al.* ARIA2: Automated NOE assignment and data integration in NMR structure calculation. *Bioinformatics* **23**, 381-382 (2007).

11 Brunger, A. T. *et al.* Crystallography & NMR system: A new software suite for macromolecular structure determination. *Acta Crystallogr. D Biol. Crystallogr.* **54**, 905-921 (1998).

12 Laskowski, R. A., Rullmann, J. A. C., MacArthur, M. W., Kaptein, R. & Thornton, J. M. AQUA and PROCHECK-NMR: Programs for checking the quality of protein structures solved by NMR. *J. Biomol. NMR* **8**, 477-486 (1996).
